# C-1 Substituted isoquinolines potentiate the antimycobacterial activity of rifampicin and ethambutol

**DOI:** 10.3389/frabi.2023.1095013

**Published:** 2023-05-09

**Authors:** Liam T. Martin, Eleanor D. Lamming, Arundhati Maitra, Parisa N. Mortazavi, Rebecca Roddan, John M. Ward, Sanjib Bhakta, Helen C. Hailes

**Affiliations:** ^1^Department of Chemistry, University College London, London, United Kingdom; ^2^Institute of Structural and Molecular Biology, Department of Biological Sciences, Birkbeck, University of London, London, United Kingdom; ^3^Department of Biochemical Engineering, The Advanced Centre for Biochemical Engineering, University College London, London, United Kingdom

**Keywords:** tuberculosis, mycobacteria, antimicrobial resistance, isoquinolines, synergism, efflux pump inhibition, antibiotic

## Abstract

**Introduction:**

The emergence of extensively drug-resistant strains of *Mycobacterium tuberculosis* threatens decades of progress in the treatment of a disease which remains one of the leading infectious causes of death worldwide. The development of novel antimycobacterial compounds is therefore essential to reinforce the existing antitubercular drug discovery pipeline. There is also interest in new compounds which can synergize with existing antitubercular drugs and can be deployed as part of a combination therapy. This strategy could serve to delay the emergence of resistance to first-line anti-tuberculosis drugs and increase their efficacy against resistant strains of tuberculosis. Previous research has established that several C-1 substituted tetrahydroisoquinolines have antimycobacterial activity. Here we sought to expand our understanding of their antimycobacterial structure activity relationships and their potential to act as adjunct therapies alongside existing antitubercular drugs.

**Methods:**

Three chemical series were synthesised and assayed for their antimycobacterial potency, mammalian cell toxicity, inhibition of whole-cell efflux and synergism with isoniazid, rifampicin, and ethambutol.

**Results:**

Several compounds were found to inhibit the growth of mycobacteria. Potent inhibitors of whole-cell efflux were also identified, as well as compounds which exhibited synergism with rifampicin and ethambutol.

**Conclusions:**

Structure-activity relationships were identified for antimycobacterial potency, improved selectivity, whole cell efflux inhibition and synergism. Potent whole-cell efflux inhibitors and synergistic compounds were identified, suggesting potential development as adjuncts to existing anti-tuberculosis chemotherapy.

## Introduction

Tuberculosis (TB) is one of the leading infectious causes of death worldwide, and the emergence of extensively drug-resistant (XDR) strains of *Mycobacterium tuberculosis* (*Mtb*) threatens decades of progress in the treatment of this disease. In 2021, the World Health Organization (WHO: http://www.who.int) estimated that 1.6 million people died of tuberculosis, and 10.6 million new cases were contracted (World Health Organisation, 2022). Of those who developed active TB in 2021, an estimated 450,000 have rifampicin-resistant or multidrug-resistant TB (RR- or MDR-TB) (World Health Organisation, 2022). Consequently, there is an urgent need for the identification of new drug candidates to strengthen the antitubercular drug development pipeline.

In recent years there has been a growing interest in antimycobacterial isoquinolines and tetrahydroisoquinolines (THIQs) (Iwasa et al., 2001; Magnet et al., 2010; Muthusaravanan et al., 2010; Matviiuk et al., 2014; Rotthier et al., 2016; Singh et al., 2017; Singh et al., 2019; Lu et al., 2020). In particular, Singh et al. (2017, 2019) have conducted structure-activity relationship (SAR) studies on a potent C-5-substituted isoquinoline which was initially identified through a high-throughput screening effort with the More Medicines for Tuberculosis (MM4TB) consortium (Magnet et al., 2010). This work led to the identification of several benzylurea derivatives which were potent inhibitors of both *Mtb* growth and the drug target inosine-5’-monophosphate dehydrogenase (IMPDH) (Singh et al., 2017; Singh et al., 2019). Lu et al. have also recently reported SAR studies of a series of *N-*5,8-trisubstituted THIQs with potent activity against *Mtb*, some of which show promising selectivity for toxicity to *Mtb* H37Rv over the VERO cell line (Lu et al., 2020). A prior SAR study from our research group found that C-1 substituted THIQs bearing a C-5-halogen and a C-8 hydroxy or methoxy group demonstrated good antimycobacterial potency, with some limited selectivity for toxicity to mycobacteria over mammalian cells (Guzman et al., 2015).

Efflux pumps are membrane-bound proteins which transport toxic substances across the bacterial cell wall and out of the cell. These proteins contribute to drug resistance by decreasing the critical concentration of drug inside the cell to a sub-inhibitory level (Machado et al., 2017; Du et al., 2018). This represents a particular problem as it increases the likelihood of resistance-conferring genetic mutations emerging (Machado et al., 2012). Combination therapy using efflux pump inhibitors (EPIs) alongside existing anti-TB drugs therefore represents a potential strategy to improve the efficacy of anti-TB drugs and limit the emergence of drug resistant TB (Rodrigues et al., 2020). Well-established EPIs include verapamil (Gupta et al., 2013; Adams et al., 2014), chlorpromazine (Rodrigues et al., 2008; Grimsey et al., 2020) and the plant alkaloid reserpine (Schmitz et al., 1998; Markham, 1999), while a range of novel efflux inhibitors of both natural (Nakamura de Vasconcelos et al., 2018; Solnier et al., 2020; Tran et al., 2020; Laws et al., 2022) and synthetic (Abate et al., 2016; Lentz et al., 2016; Scalacci et al., 2017; Lentz et al., 2018; Sen et al., 2018; Laws et al., 2022) origin have been identified in recent years. It has been reported that the use of verapamil in combination with rifampicin or bedaquiline resulted in reductions in their *in vitro* minimum inhibitory concentration (MIC) against *Mtb* of 4-fold or 8-fold, respectively (Gupta et al., 2014; Singh et al., 2014). Studies performed in *in vivo* mouse models have also demonstrated the efficacy of verapamil in combination with isoniazid, rifampicin and pyrazinamide (Gupta et al., 2013). Furthermore, co-administration of verapamil and bedaquiline has been shown to allow for lower dosing and while reducing the emergence of drug-resistance in mice (Gupta et al., 2014). These studies highlight the potential of EPIs to increase the efficacy of the existing anti-TB arsenal, but there are currently no inhibitors of mycobacterial efflux pumps in clinical use.

In this work, we aimed to further probe the antimycobacterial potential of the THIQ scaffold through the synthesis and SAR analysis of three groups (I-III) of analogues. The high-throughput spot-culture growth inhibition (HT-SPOTi) assay (Danquah et al., 2016) was used to determine the MIC of all compounds against the *Mtb* model organism *M. aurum* (Gupta and Bhakta, 2012), with a selection of compounds additionally screened against *M. bovis* BCG (Altaf et al., 2010). Compounds were also assayed for mammalian cell cytotoxicity against murine RAW 264.7 cells with some compounds also screened against the human THP-1 cell line. This allowed for a determination of the selectivity of these compounds for toxicity to mycobacteria over mammalian cells. To provide preliminary insights into the synergistic properties of some of the compounds, including brominated analogues which had previously been reported as having potentially interesting activities, selected compounds were evaluated for their ability to inhibit whole-cell efflux mechanisms of *M. aurum* using an ethidium bromide accumulation assay (Rodrigues et al., 2008; Paixão et al., 2009) and for their ability to synergize with isoniazid, rifampicin and ethambutol using a HT-SPOTi checkerboard method (Guzman et al., 2013).

Structure-activity relationships for the antimycobacterial activity of this compound class were established. THIQs which were able to inhibit multidrug efflux in *M. aurum* were also identified, with initial insights made into the structural features associated with efflux inhibition. In addition, four THIQs were found to synergize with either rifampicin or ethambutol at low concentrations, presenting the opportunity to further develop these compounds as adjunct therapies to the existing first-line antituberculosis drug regimen.

## Materials and methods

### Bacterial strains, eukaryotic cell lines and culture media

The bacterial species used in this study were *Mycobacterium aurum* (ATCC 23366) and *M. bovis* BCG Pasteur (ATCC 35734). The mammalian cell lines used for cytotoxicity assays were the murine macrophage cell line RAW 264.7 (ATCC-71) and human leukemia monocytic cell line THP-1 (ATCC TIB-202). Mycobacteria were cultured in Middlebrook 7H9 broth (BD Difco™) or Middlebrook 7H10 agar media (BD Difco™) supplemented with albumin/dextrose/catalase (ADC, Remel™) or oleic acid/albumin/dextrose/catalase (OADC, BD Difco™) enrichments, respectively. Mammalian cell lines were cultured in Roswell Park Memorial Institute (RPMI) 1640 media with L-glutamine (Gibco^®^) supplemented with fetal bovine serum (FBS, Sigma Aldrich, Non-US origin, sterile filtered, heat-inactivated).

### Synthetic methods

The procedures employed for chemical synthesis are reported in the **Supplementary Information** together with the relevant characterization data.

### High-throughput spot culture growth inhibition assay

All compounds were dissolved in dimethyl sulfoxide (DMSO) to a concentration of 50 mg/ml. 2 µl aliquots from a 2-fold serial dilution of this stock were transferred into a 96-well microtiter plate. Isoniazid was included as a positive control and DMSO as a negative control. Middlebrook 7H10 agar medium containing 0.5% glycerol and 10% (v/v) OADC (200 µl) was dispensed into each well and allowed to solidify. A mid-logarithmic phase culture of *M. aurum* or *M. bovis* BCG was diluted in Middlebrook 7H9 broth to give a cell density of 1 x 10^6^ cells/ml (Gupta and Bhakta, 2012) and then dispensed (2 µl, ~ 2000 cells) into each well aseptically. The plates were then incubated at 35 °C (for *M. aurum*) and 37 °C (for *M. bovis* BCG). The results were observed after 5 days for *M. aurum* or 14 days for *M. bovis* BCG. MICs were visually determined by observing the lowest concentration of each compound at which no bacterial growth could be observed. These assays were performed in biological triplicate.

### Mammalian resazurin microtiter assay

All compounds were dissolved in DMSO to a concentration of 50 mg/ml. Aliquots (2 µl) from a 2-fold serial dilution of this stock were transferred into a 96-well microtiter plate, with a DMSO-only well included as a negative control. Cultures of RAW 264.7 or THP-1 cells (5 ml), were diluted to a cell density of 5 x 10^5^ cells/ml in RPMI and aliquots (100 µl) were then transferred into each well of the 96-well plate. One row of the 96-well plate was left without cells, as a sterility control. The cells were then incubated without shaking (37 °C, 5% CO_2_) for 48 hours. The spent media was removed by pipetting and the media was replaced with complete RPMI (170 µl) and resazurin solution (0.01%, 30 µl). For non-adherent THP-1 cells, the cells were pelleted by centrifugation of the 96-well plates (270 x g, 2 min) before aspiration of the media. The plates for both cell lines were then incubated for a further 24 h (37 °C, 5% CO_2_). Growth inhibitory concentrations (GICs) were visually determined by observing the lowest concentration of each compound at which the resazurin dye was not reduced from blue to pink. Visual determinations were confirmed by fluorescence readings (Excitation wavelength: 560 nm, Emission wavelength: 590 nm) for each plate using a BioTek^®^ Synergy 2™ Multi-Detection Plate Reader. These assays were performed in biological triplicate.

### Assaying whole-cell drug efflux pump inhibition

The assay was modified from a previously published protocol (Rodrigues et al., 2008). Early log phase cells of *M. aurum* (OD_600_ ∼ 0.8) were harvested by centrifugation and resuspended in 1 x PBS to an OD_600_ of 0.4. The test samples contained 4−6 × 10^7^ CFU/ml in PBS, glucose (0.4%), ethidium bromide (0.5 mg/l), and the compounds of interest at ¼ × MIC. Blank samples contained all of the components mentioned above, except the bacterial suspension, which was replaced with 1 × PBS. Verapamil, a known efflux pump inhibitor, was used as the positive control at a concentration of 125 µg/ml. The experiment was performed in a fluorimeter (FLUOstar OPTIMA, BMG Labtech) programmed with the following parameters: wavelengths of 544 nm for excitation and 590 nm for detection of fluorescence, gain 2200, a temperature of 37°C, and a cycle of measurement every minute for a total period of 60 min. The accumulation or efflux of ethidium bromide was monitored on a real-time basis. The ability of compounds to inhibit ethidium bromide efflux was quantified by determining the relative final fluorescence (RFF) of each compound. This was calculated according to **Formula 1**:


$$RFF=\frac{{RF}_{\text{treated}}–{RF}_{\text{control}}}{{RF}_{\text{control}}}$$

RF_treated_ is the relative fluorescence (RF) at the last time point of the EtBr accumulation assay for the experimental sample, while RF_control_ is the RF of the untreated control sample at the last time point of the assay (Machado et al., 2011; Coelho et al., 2015).

### Synergy/antagonism assay

The synergistic effect of THIQs were examined in combination with isoniazid, ethambutol and rifampicin against *M. aurum*. The assay was conducted in a 96-well microtiter plate using a SPOTi checkerboard distribution as reported previously (Guzman et al., 2013). The compounds were serially diluted in DMSO across a range of concentrations spanning both above and below the MIC of each compound/drug. Each row of the 96-well plate contained different concentrations of the THIQ derivatives, and each column contained different concentrations of the first-line anti-TB drug. The checkerboard was constructed by adding 1 µl of each of the stock concentrations to the corresponding well and then dispensing 200 µl of warm Middlebrook 7H10 agar medium. The plates were then spotted with *M. aurum*. The plates were incubated in sealed bags at 35° C for 5 days and Fractional Inhibitory Concentration Indices A FICI value were calculated using **Formula 2**:


$$FICI=\frac{*{MIC}_{A}\;in\;presence\;of\;B}{{MIC}_{A}}+\frac{{}^{\circ}{MIC}_{B}\;in\;presence\;of\;A}{{MIC}_{B}}$$

An FICI value ≤ 0.5 indicates synergism; a value between 0.5 and 4.0 indicates no interaction; and a value higher than 4.0 indicates antagonism (Odds, 2003).

## Results

### Synthesis

An initial group of C-1 substituted THIQs (Group I) was prepared *via* a phosphate-mediated, biomimetic Pictet-Spengler reaction between dopamine hydrochloride and a range of aldehydes (**Table 1**) (Pesnot et al., 2011). Both the major (6,7-, **1-10a**) and minor (7,8-, **1-10b**) regioisomers were isolated by preparative HPLC (Guzman et al., 2015). The C-1 alkyl chain length was systematically explored, as previous work had highlighted a longer alkyl chain at this position as having promising activities. C-1 cyclopentyl, 2-methyl benzyl and 3,4-methylenedioxybenzyl moieties were also explored, the latter of which had been present in several active analogues identified previously (Guzman et al., 2015). Moreover, a hydroxyl group at C-8 had also been associated with higher activities in some cases, so both 6,7- and 7,8-regioisomers were generated (Guzman et al., 2015). All compounds were prepared using commercially available aldehydes with the exception of **9a** and **9b**, for which (*m*-tolyl)acetaldehyde was prepared from the commercially available alcohol by a Parikh-Doering oxidation (Parikh and Doering, 1967), and **10a** and **10b**, for which 3,4-(methylenedioxy)phenylacetaldehyde was prepared as previously described (Guzman et al., 2015). Regioisomers **11a** and **11b** were prepared from a Pictet-Spengler reaction between *m*-tyramine and hexanal. THIQs **4a.HCl ** and **11a.HCl** were prepared as the hydrochloride salts of their respective free-bases. The enantio-enriched THIQs (1*S*)-**4a.HCl ** and (1*S*)-**11a.HCl** were synthesized using norcoclaurine synthase (*Tf*NCSΔ33) in a biocatalytic Pictet-Spengler reaction between hexanal and dopamine or *m*-tyramine, respectively (**Table 1**) and were included in Group I to explore C-1 stereochemistry (Pesnot et al., 2012; Ruff et al., 2012; Lichman et al., 2015).

**Table 1:** Synthesis of Group I THIQs.

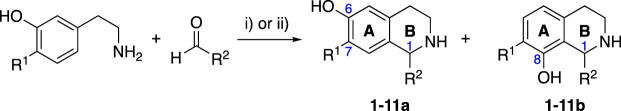				
THIQ			Yield	
	R^1^	R^2^	a	b
**1**	OH	CH_2_CH_3_	77%	4%
**2**	OH	CH_2_CH_2_CH_3_	45%	3%
**3**	OH	CH_2_(CH_2_)_2_CH_3_	83%	5%
**4**	OH	CH_2_(CH_2_)_3_CH_3_	75%	9%
(1*S*)-**4a.HCl**	OH	CH_2_(CH_2_)_3_CH_3_	18% (78% ee)	–
**5**	OH	CH_2_(CH_2_)_4_CH_3_	86%	10%
**6**	OH	CH_2_(CH_2_)_5_CH_3_	85%	11%
**7**	OH	CH_2_(CH_2_)_6_CH_3_	77%	8%
**8**	OH	cyclopentyl	32%	4%
**9**	OH	CH_2_(3-MeC_6_H_5_)	69%	3%
**10**	OH	CH_2_(3,4-OCH_2_OC_6_H_3_)	49%	3%
**11**	H	CH_2_(CH_2_)_3_CH_3_	81%	12%
(1*S*)-**11a.HCl**	H	CH_2_(CH_2_)_3_CH_3_	11% (79% ee)	–

A-ring and B-ring designation indicated on product structures. For racemic **1a‑11a** and **1b-11b**: i) phosphate buffer (0.1 M, pH 6)/MeCN (1:1), 50 °C, 18 h. For (1*S*)-**4a.HCl** and (1*S*)-**11a.HCl**: ii) *Tf*NCSΔ33, HEPES/MeCN (9:1, pH 7.5), 37 °C, 18 h.

**Scheme 1:**
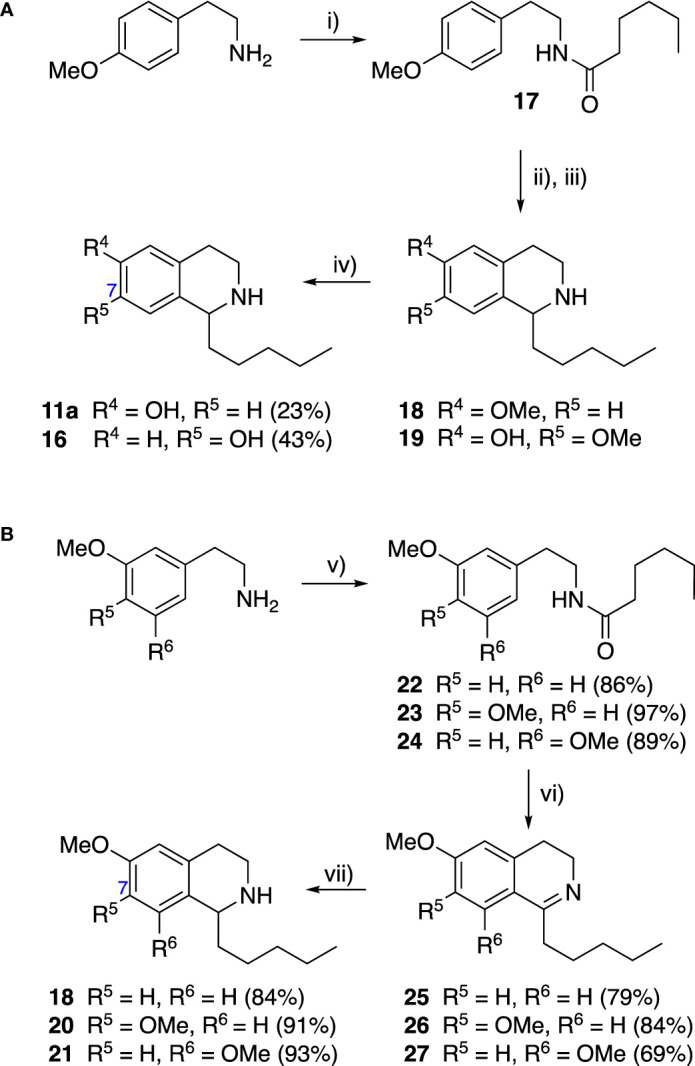
**(A)** Synthesis of 7-hydroxy-substituted THIQ **16**. *Reaction conditions*: i) hexanoyl chloride, Et_3_N, CH_2_Cl_2_, (90%), ii) P_2_O_5_, POCl_3_, xylene, 20 h, iii) NaBH_4_, MeOH (37% over two steps, 1:1.6 ratio of regioisomers **18** and **19**), iv) BBr_3_, CH_2_Cl_2_, 78°C, rt, 24 h. **(B)** Synthesis of 6-methoxy-, 7-methoxy-, and 8-methoxy-substituted THIQs **18**, **21** and **22**. *Reaction conditions*: v) B(OCH_2_CF_3_), TAME, reflux, 18 h, vi) POCl_3_, xylene, reflux, 18 h, vii) NaBH_4_, MeOH, rt, 18 h.

Group II was designed primarily to further the exploration of A-ring substituents, including hydroxy substitutions unexplored in Group I; C-5 bromo substitution and A-ring methoxy substituents. A C-1 pentyl chain was retained for all compounds as the most potent and selective compounds in Group I featured this substituent (see below). The initial phenethylamines required for the synthesis of this series were either acquired commercially or prepared by reduction of the appropriate phenylacetonitrile (see **supplementary information**). Brominated phenethylamines were prepared by bromination and subsequent demethylation of the appropriate methoxy phenethylamines (**Scheme S1**). THIQs **12**-**15** were then prepared *via* a phosphate-mediated Pictet-Spengler reaction (**Table 2**) (Pesnot et al., 2011). THIQ **16** with a C-7 hydroxyl group was also prepared to compare to **11a** which features a C-6 hydroxyl group. The synthesis of **16** (**Scheme 1A**) began with an initial amide coupling reaction between 4-methoxyphenyethylamine and hexanoic acid to give **17**. This was followed by Bischler-Napieralski cyclisation (Heravi et al., 2014) and subsequent imine reduction to afford **18** and **19** as a mixture of regioisomers. These compounds were then demethylated and separated by preparative HPLC to isolate **16** (and **11a**; **Scheme 1A**). Methoxy-substituted THIQs at C-6; C-6 and C-7; or C-6 and C-8 (**18**, **20** and **21**) were synthesized in a similar manner (**Scheme 1B**) to compare to their hydroxy-substituted counterparts **4a**, **11a** and **12**. First, amides **22**-**24** were prepared by a Sheppard amidation (Sabatini et al., 2017), before Bischler-Napieralski cyclisation to afford dihydroisoquinolines **25**-**27**, which then underwent imine reduction to give the desired THIQs **18, 20** and **21**.

**Table 2:** Synthesis of Group II THIQs.

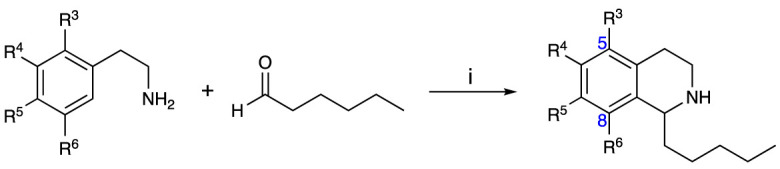					
THIQ	R^3^	R^4^	R^5^	R^6^	Yield
**12**	H	OH	H	OH	57%
**13**	Br	H	H	OH	14%
**14**	Br	OH	H	OH	7%
**15**	Br	H	OH	OH	72%

i) Phosphate buffer (0.1 M, pH 6)/MeCN (1:1), 50 °C, 18 h.

**Scheme 2:**
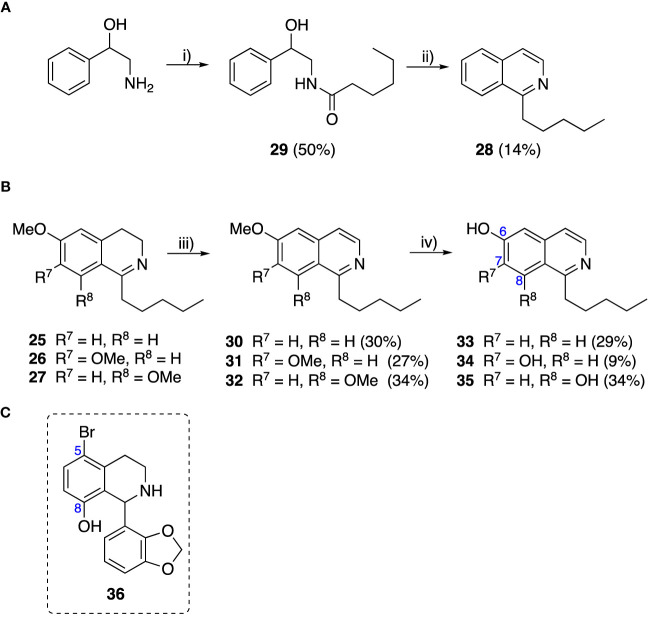
**(A)** Synthesis of A-ring unsubstituted isoquinoline. *Reaction conditions*: i) B(OCH_2_CF_3_)_3_, TAME, reflux, 18 h (50%), ii) POCl_3_, xylene, reflux, 18 h. **(B)** Synthesis of A-ring substituted isoquinolines. *Reaction conditions*: iii) Pd/C, 150°C, iv) BBr_3_, CH_2_Cl_2_, 18 h. **(C)** Structure of **36**.

To explore the influence of B-ring aromaticity, Group III was prepared. This group contained fully aromatic isoquinolines rather than THIQs. The A-ring-unsubstituted isoquinoline **28** was prepared by first synthesizing β-hydroxy amide **29** *via* a Sheppard amidation (Sabatini et al., 2017) between 2-amino-1-phenylethanol and hexanoic acid (**Scheme 2A**). This was followed by a Pictet-Gams cyclisation (Wang, 2010) to directly afford the fully aromatic isoquinoline. A-ring substituted isoquinolines were prepared by oxidation of previously prepared dihydroisoquinolines **25**-**27** with Pd/C under air to afford the methoxy-substituted compounds **30**-**32** (**Scheme 2B**) (Awuah and Capretta, 2010). These were then demethylated to afford their hydroxy-substituted counterparts **33**-**35**. Compound **36** (**Scheme 2C**) was synthesized as previously reported, for use in synergism studies (Guzman et al., 2015).

### Potency and selectivity

Generally, for the 6,7-dihydroxy-substituted THIQs, **1a**-**7a**, potency against *M. aurum* was found to increase with increasing chain length (**Table 3**). Toxicity to RAW 264.7 cells was high for THIQs **1a**, **2a**, **4a**, **6a** and **7a**. However, **3a** and **5a** displayed lower cytotoxicity, with GIC values of ≥ 62.5 µg/ml, indicating a non-linear relationship between carbon chain length and cytotoxicity. The same trends were not observed for the 7,8-dihydroxy-substituted THIQs, **1b**-**7b**, all of which had MIC values of ≥ 125 µg/ml, with no clear relationship between chain length and activity (**Table 3**). All of these compounds had high cytotoxicity against the RAW 264.7 cell line with GIC values of 15.6 µg/ml, suggesting no relationship between chain length and cytotoxicity. Of the alkyl chain-bearing THIQs, the C-1 pentyl THIQs, **4a** and **4b**, appeared to confer the best balance of antimycobacterial potency and mammalian cell cytotoxicity. For the THIQs with cyclic C-1 substituents, it was observed that the 7,8-hydroxy-substituted examples had greater potency against *M. aurum* than their 6,7-substituted counterparts (**Table 3**). However, these compounds were also more toxic to RAW 264.7 cells and did not show improved selectivity. Only the 3,4-methylenedioxybenzyl moiety (**10a** & **10b**) was found to confer comparable potency and selectivity to the C-1 pentyl group (**4a** & **4b**). The 6- and 8-mono-hydroxy-substituted THIQs (**11a** & **11b**) were observed to have similar activity to **4a** and **4b** (**Table 3**). Both **4a.HCl** and **11a.HCl** were found to have comparable activity to their respective free-bases, **4a** and **11a**, however the hydrochloride salts were found to exhibit reduced cytotoxicity against the RAW 264.7 cell line. Many THIQs and isoquinolines in Group II and all of Group III were therefore formulated as their HCl salts to reduce cytotoxicity and to improve the solubility of the more lipophilic compounds. Interestingly, (1*S*)-**4a.HCI** and (1*S*)-**11a.HCI**, were found to have low potency and cytotoxicity. When compared to the equivalent racemic THIQs, these results suggest that the (1*R*)-THIQs may be more potent and cytotoxic than the corresponding (1*S*)-THIQs. All compounds in this series displayed selectivity for toxicity to the mammalian RAW 264.7 cells over *M. aurum*; an issue which would require optimization to overcome.

**Table 3:** Antimycobacterial and cytotoxicity data for Group I.

		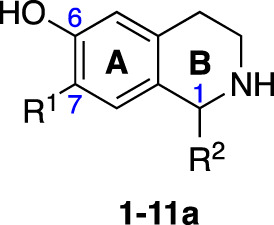			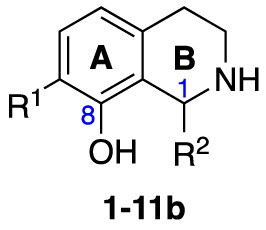		
THIQ	R^2^	MIC_99_	GIC_90_	SI	MIC_99_	GIC_90_	SI
**1**	CH_2_CH_3_	> 500 (> 2587)	15.6 (81)	< 0.03	250 (1294)	15.6 (81)	0.06
**2**	CH_2_CH_2_CH_3_	> 500 (> 2412)	31.3 (151)	< 0.06	250 (1206)	15.6 (75)	0.06
**3**	CH_2_(CH_2_)_2_CH_3_	> 500 (> 2259)	125 (565)	< 0.25	250 (1130)	15.6 (70)	0.06
**4**	CH_2_(CH_2_)_3_CH_3_	500 (2125)	31.3 (133)	0.06	125 (531)	15.6 (66)	0.125
**4.HCl**	CH_2_(CH_2_)_3_CH_3_	500 (1840)	125 (460)	0.25	–	–	–
(1*S*)-**4.HCl**	CH_2_(CH_2_)_3_CH_3_	> 500 (1955)	250 (977)	0.5	–	–	–
**5**	CH_2_(CH_2_)_4_CH_3_	250 (1003)	62.5 (251)	0.25	250 (1003)	15.6 (63)	0.062
**6**	CH_2_(CH_2_)_5_CH_3_	250 (949)	7.8 (30)	0.03	500 (1898)	15.6 (59)	0.031
**7**	CH_2_(CH_2_)_6_CH_3_	125 (451)	7.8 (28)	0.06	250 (901)	15.6 (56)	0.062
**8**	cyclopentyl	> 500 (> 2143)	62.5 (268)	< 0.125	250 (1072)	7.8 (33)	0.031
**9**	CH_2_(3-MeC_6_H_5_)	500 (1856)	31.3 (116)	0.06	250 (928)	15.6 (58)	0.062
**10**	CH_2_(3,4-OCH_2_OC_6_H_3_)	500 (1670)	62.5 (209)	0.125	250 (835)	31.3 (105)	0.125
**11**	CH_2_(CH_2_)_3_CH_3_	500 (2280)	62.5 (285)	0.125	250 (1140)	31.3 (143)	0.125
**11.HCl**	CH_2_(CH_2_)_3_CH_3_	500 (1955)	125 (489)	0.25	–	–	–
(1*S*)-**11.HCl**	CH_2_(CH_2_)_3_CH_3_	> 500 (> 1840)	> 500 (> 1840)	<1	–	–	–
**INH**	**-**	0.98 (7.1)	> 500 (> 3646)	> 510	–	–	–

Antimycobacterial MIC values were obtained by HT-SPOTi assay against *M. aurum*. GIC values were determined by resazurin-based mammalian cell cytotoxicity assay. MIC and GIC values are reported in µg/ml, with molarity given in brackets in µM. SI values were calculated by dividing GIC by MIC. INH = isoniazid. All THIQs were screened as the free amine unless otherwise indicated. **R^1^** = OH for all compounds, with the exception of **11**, **11.HCl** and (1*S*)-**11.HCl**, for which **R^1^** = H.

In THIQ Group II (**Table 2** and **Scheme 1**), it was observed that the transition away from catechol-bearing THIQs generally resulted in increased potency and selectivity (**Table 4**). Dimethoxy-substituted THIQ **21** was the most promising compound in this series, with the highest potency observed against both *M. aurum* and *M. bovis* BCG. The high cytotoxicity of this compound against RAW 264.7 resulted in SI values of 1 or 2 (*M. aurum* or *M. bovis* BCG, respectively). These SI values are in line with those observed for the most selective THIQs reported in our previous work (Guzman et al., 2015). The two dimethoxy-substituted THIQs, **20** and **21**, were also among the most selective compounds in the series, suggesting that A-ring methoxy-substitution may improve selectivity.

**Table 4:** Antimycobacterial and cytotoxicity activity for Group II.

THIQ		MIC_99_		GIC_90_	SI	
		*M. aurum*	*M. bovis* BCG	RAW 264.7	*M. aurum*	*M. bovis* BCG
**12**	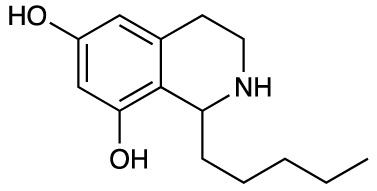	250 (920)	250 (920)	125 (460)	0.5	0.5
**13**	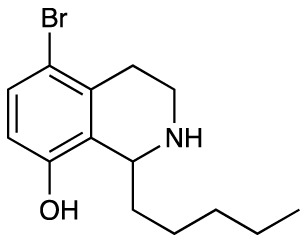	62.5 (210)	–	15.6 (52)	0.25	–
**14**	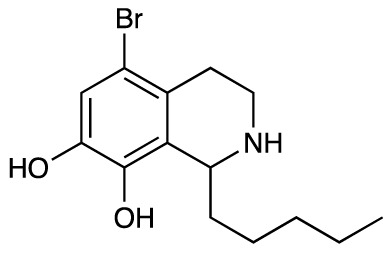	250 (796)	–	31.3 (100)	0.125	–
**15**	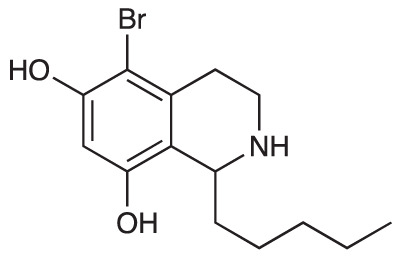	125 (398)	–	31.3 (100)	0.25	–
**16**	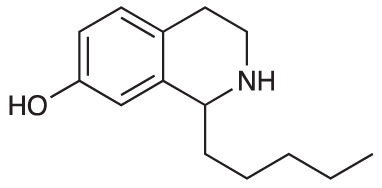	500 (2280)	–	125 (570)	0.25	–
**18**	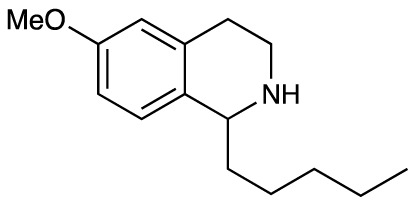	125 (463)	31.25 (134)	31.25 (134)	0.25	1
**20**	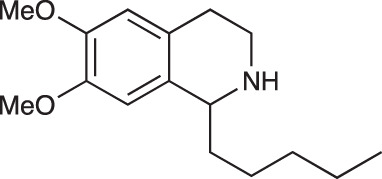	125 (417)	62.5 (208)	125 (417)	1	2
**21**	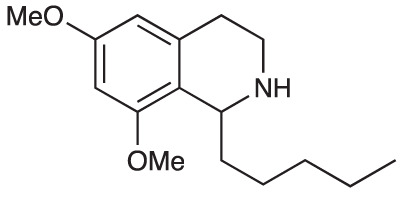	31.25 (104)	15.6 (52)	31.25 (104)	1	2
**INH**	**-**	0.98 (7.1)	0.10 (0.7)	> 500 (> 3646)	> 510	> 5000

Antimycobacterial MIC values were obtained by HT-SPOTi assay. GIC values were determined by resazurin-based mammalian cell cytotoxicity assay. MIC and GIC values are reported in µg/ml, with molarity given in brackets in µM. SI values were calculated by dividing GIC by MIC. INH = isoniazid. Compounds **13**-**16** were screened as the free base. All other THIQs were screened as the hydrochloride salt.

The isoquinolines in Group III (**Schemes 2A, B**) were found to have similar activity to their THIQ counterparts (**Table 5**), suggesting that B-ring aromaticity had a limited influence on the activity of these compounds. A notable exception to this trend is **33**, which was observed to be significantly more antimycobacterial and selective than the corresponding THIQ, **11a**. This compound is also notable for having an SI of 2 for both *M. aurum* and *M. bovis* BCG over RAW 264.7 cells and is the only compound in this study with any selectivity for toxicity to *M. aurum* over the RAW 264.7 cell line. Compound **32** was the most potent compound in this series against both mycobacterial species. It was not found to be selective for toxicity to *M. aurum* but had an SI of 2 for *M. bovis* BCG over RAW 264.7.

**Table 5:** Antimycobacterial and cytotoxicity data for Group III isoquinolines.

Isoquinoline		MIC_99_		GIC_90_	SI	
		*M. aurum*	*M. bovis* BCG	RAW 264.7	*M. aurum*	*M. bovis* BCG
**28**	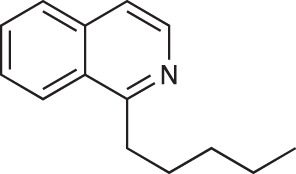	> 250 (> 1063)	250 (1063)	500 (2127)	< 2	2
**30**	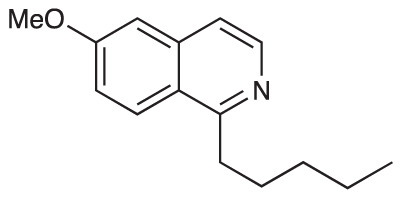	> 250 (943)	125 (471)	250 (943)	< 1	2
**31**	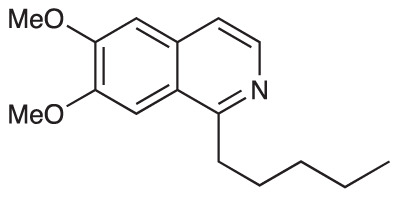	500 (1694)	125 (424)	250 (847)	0.5	2
**32**	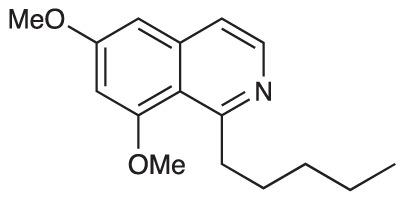	31.25 (106)	15.6 (53)	31.25 (106)	1	2
**33**	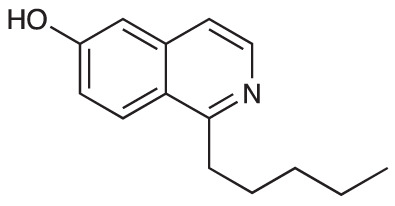	62.5 (249)	62.5 (249)	125 (498)	2	2
**34**	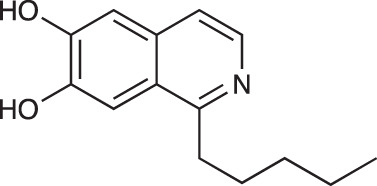	> 500 (> 1872)	> 500 (> 1872)	250 (936)	< 0.5	< 2
**35**	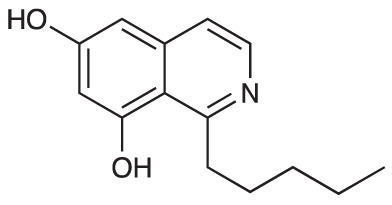	> 500 (> 1872)	62.5 (233)	62.5 (233)	< 0.125	1
**INH**	**-**	0.98 (7.1)	0.10 (0.7)	> 500 (> 3646)	> 510	> 5000

Antimycobacterial MIC values were obtained by HT-SPOTi assay. GIC values were determined by resazurin-based mammalian cell cytotoxicity assay. MIC and GIC values are reported in µg/ml, with molarity given in brackets in µM. SI values were calculated by dividing GIC by MIC. INH = isoniazid. All isoquinolines were screened as the hydrochloride salt.

The compounds in Group III were also screened for cytotoxicity against the human THP-1 cell line, alongside selected compounds from Groups I and II (**Table S1**). In general, these compounds showed similar cytotoxicity against this cell line, however, some displayed lower cytotoxicity, resulting in increased SI values. Notably, Compound **11a** had an SI value of 2 for activity against *M. bovis* BCG over THP-1 cells, while **21** and **30** had SI values of 4.

### Efflux pump inhibition

The 6,7-dihydroxy-THIQs (**2a**, **5a** & **6a**) displayed little to no inhibition of ethidium bromide efflux (**Table 6**). However, all 7,8-dihydroxy-THIQs (**2b**-**5b**), with the exception of **6b,** displayed efflux inhibition. **5b** resulted in the greatest accumulation of ethidium bromide of the THIQs tested, with an RFF of 1.42, demonstrating some potency as an efflux inhibitor, but still significantly less than the RFF of 2.09 observed for the positive control Verapamil. The activity of the 7,8-dihydroxy substituted THIQs alongside the inactivity of the 6,7-dihydroxy-subsituted analogues, suggested that either a C-8 hydroxy substituent was a requirement for efflux pump inhibition, or that a C-6 hydroxy substituent abolishes this activity. The activity of the 5-bromo-substituted THIQ **13** suggested that a bromide atom at this position may confer efflux pump inhibition, while the low activity of **36** demonstrated that the C-1 substituent is also an important factor in efflux pump inhibition. Interestingly, no correlation was observed between efflux pump activity and compound MIC.

**Table 6:** Efflux pump inhibition by selected THIQs.

THIQ		RFF
**2a**	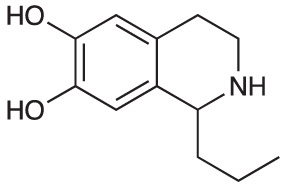	-0.07
**2b**	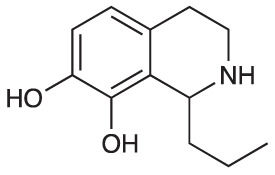	1.16
**3b**	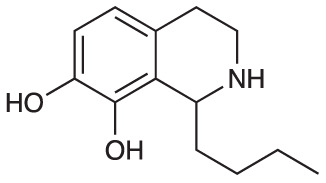	1.13
**4b**	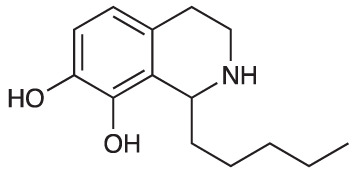	1.26
**5a**	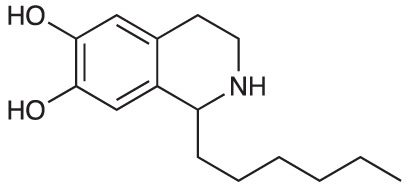	0.39
**5b**	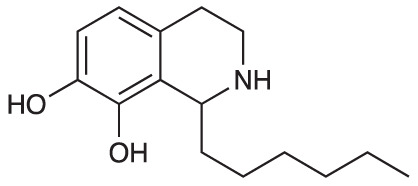	1.42
**6a**	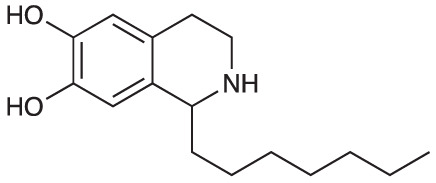	0.46
**6b**	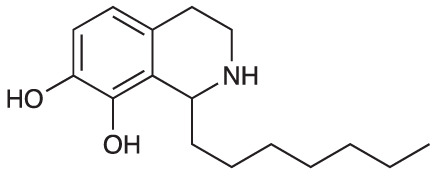	-0.03
**13**	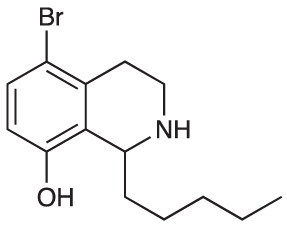	1.06
**36**	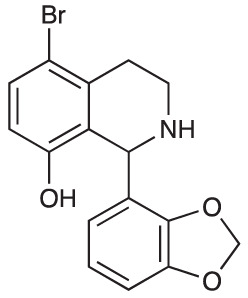	0.32
**Verapamil**	–	2.09

Relative final fluorescence (RFF) calculated according to formula 1.

### Synergism

The most notable trend from the synergism studies (**Table 7**) is that the 5-bromo-substituted THIQs, **15** and **36**, were both able to affect a 2-fold increase in the potency of rifampicin at a concentration of 0.1 µg/ml, with FICIs of 0.5, indicating synergism. Compound **7b** was also found to have the same activity alongside rifampicin. None of the compounds assayed were found to have synergistic activity with isoniazid, and only compound **5b** was found to have any synergistic activity with ethambutol, causing a 2-fold increase in potency, at a concentration of 0.1 µg/ml.

**Table 7:** Synergism Data.

THIQ	*MIC_A_*	°INH (*THIQ)	FICI	°RIF (*THIQ)	FICI	°EMB (*THIQ)	FICI
**4b**	125	1.875 (62.5)	2.50	0.312 (15.75)	0.63	0.15 (>125)	1.50
**5b**	250	1.875 (31.25)	2.13	0.625 (250)	1.00	0.15 (0.1)	0.50
**7b**	250	1.875 (125)	2.50	0.312 (0.1)	0.50	0.312 (250)	1.00
**9b**	250	0.938 (250)	1.00	0.312 (125)	1.00	0.312 (250)	1.00
**10b**	250	1.875 (62.5)	2.25	0.625 (250)	1.00	0.312 (250)	1.00
**13**	62.5	0.468 (62.5)	1.50	0.312 (0.97)	0.52	0.15 (>15.6)	0.75
**15**	125	0.468 (125)	1.50	0.312 (0.1)	0.50	0.15 (>62.5)	1.00
**36**	125	0.938 (125)	1.00	0.312 (0.1)	0.50	0.312 (125)	1.00

For each first-line antituberculosis drug, the lowest MIC value measured in the checkerboard assay is given, with the minimum THIQ concentration at which this was achieved given below in brackets. MIC_B_ values for: isoniazid = 0.98 µg/mL, rifampicin = 0.49 µg/mL, ethambutol = 0.24 µg/mL (from this study). All THIQs were assayed as the free base unless otherwise indicated. MIC values determined against *M. aurum* are given in μg/ml.

## Discussion

Structure activity relationships were established for C-1 substituted THIQs and isoquinolines. An *n*-pentyl chain was found to be the optimal length of C-1 alkyl substituent, while the 3,4-methylenedioxyphenyl group was also found to perform well in this position. A comparison of the (1*S*)-THIQs, (1*S*)-**4a.HCl** and (1*S*)-**11a.HCl**, with their racemic counterparts suggested that (1*R*)-THIQs may be more potent and cytotoxic than the corresponding (1*S*)-THIQs. This suggests that the (1*R*)-THIQs may confer no advantage in selectivity, as increased cytotoxicity would negate any gains in potency. However, only two pair-wise matches were compared in this study, and it may be yet possible to leverage the stereochemistry of this scaffold to improve the selectivity of other THIQs with further research.

It was also observed that the replacement of A-ring hydroxy groups with methoxy groups typically increased selectivity. The presence of a C-8 methoxy substituent was typically found to confer greater antimycobacterial potency to compounds in this study. Notably, the 6,8-dimethoxy-substituted THIQ **21** was found to be a potent inhibitor of *M. bovis* BCG growth (MIC = 15.6 µg/ml), while also having an SI of 4 over the human THP-1 cell line. This suggests that the careful selection of A-ring substituents can be an effective approach to engineering the activity of this compound class. A-ring substitution was found to have a much greater influence on the activity of these compounds than B-ring aromaticity, for which no clear impact on compound activity could be determined.

For those compounds which were screened against both *M. aurum* and *M. bovis* BCG, it was notable that greater potency was typically observed against the latter. While both are valid model organisms for *Mtb*, *M. bovis* BCG is the more closely related of the two (Garnier et al., 2003; Altaf et al., 2010; Gupta and Bhakta, 2012; Phelan et al., 2015), and so it may be expected that the antitubercular activity of these compounds more closely reflects that observed against *M. bovis* BCG. Small differences in cytotoxicity were also observed when screening against THP-1 over RAW 264.7, however these were minor and both mammalian cell lines generally gave similar results.

Overall, despite the identification of key structure activity relationships, no compound in this study was sufficiently potent or selective to take forward for hit-to-lead optimisation (Katsuno et al., 2015). In order to conduct further optimization of the most promising compounds in from this study it would be prudent to engage in further exploration of A and B-ring substituents. Potent antitubercular isoquinolines and THIQs with large substituents in the C-5 and C-8 positions have been reported in the literature and it may be possible to take inspiration from these studies to build upon the most promising C-5 and C-8 substituted compounds reported in this work (Magnet et al., 2010; Singh et al., 2017; Singh et al., 2019; Lu et al., 2020). *N*-substituted THIQs could also be explored. This would increase the lipophilicity of the THIQs in this series, potentially improving their permeability through the mycobacterial membrane. Several potent antitubercular *N*-substituted THIQs have also been reported previously (Guzman et al., 2010; Matviiuk et al., 2014; Rotthier et al., 2016).

An area in which these compounds may be developed to add value in the fight against DR-TB is as EPIs for use in combination with existing antitubercular drugs. Several compounds, at their sub-MICs, were found to be inhibitors of whole-cell efflux of ethidium bromide, and some early structure activity relationships have been identified. As only hydroxyl and bromo substituents have so far been explored in these assays, there is still space to expand on these to identify more potent efflux inhibitors with improved drug-like properties.

Four THIQs (**5b**, **7b**, **15** and **36**) were found to synergize with either rifampicin or ethambutol and it was observed that a 5-bromo substituent appeared important in conferring synergism with rifampicin, though other substituents in this position are yet to be explored. These compounds therefore merit further study and optimization for their synergistic activity. That synergism was observed at a concentration significantly below their GIC values suggests that these compounds may have a broad therapeutic window when applied as an adjunct therapy. Further studies will therefore be required to unpick the relationship between efflux inhibition and synergism in these cases, and to confirm the method through which synergism was occurring.

## Data availability statement

The original contributions presented in the study are included in the article/**Supplementary Material**. Further inquiries can be directed to the corresponding authors.

## Author contributions

LM and EL synthesized compounds. LM, AM, and PM performed assays and evaluated biological data. RR and JW purified enzymes for biocatalytic reactions. HCH and SB contributed conceptualization and supervision. LM wrote the first draft of the article. All authors contributed to designing the article. All authors contributed to the article and approved the submitted version.
