# Supplementary material for: C-1 Substituted isoquinolines potentiate the antimycobacterial activity of rifampicin and ethambutol
Source: Front Antibiot. 2023 May 9;2:1095013. doi: 10.3389/frabi.2023.1095013 (PMC11731654; doi:10.3389/frabi.2023.1095013)
Supplement: Supplementary file 1 [file DataSheet_1.docx]

**C-1 Substituted Isoquinolines Potentiate the Antimycobacterial Activity of Rifampicin and Ethambutol – Supplementary Information**

Liam T. Martin, Eleanor D. Lamming, Arundhati Maitra, Parisa N. Mortazavi, Rebecca Roddan, John M. Ward, Sanjib Bhakta^*^, Helen C. Hailes^*^ (^*^equal contribution)

**Supplementary Figures**

| **** |
| --- |
| **Scheme S1.** The synthesis of substituted phenethylamines**.** i) BH_3_.THF, THF ii) BBr_3_, CH_2_Cl_2_ iii) Br_2_, AcOH iv) BBr_3_, CH_2_Cl_2._ |

| **Table S1. Antimycobacterial and THP-1 Cytotoxicity Data for Group III and Selected THIQs.** Antimycobacterial MIC values were obtained by HT-SPOTi assay. GIC values were determined by resazurin-based mammalian cell cytotoxicity assay. MIC and GIC values are reported in µg/ml, with molarity given in brackets in µM. SI values were calculated by dividing GIC by MIC. INH = isoniazid. All compounds were screened as the hydrochloride salt. | | | | | |
| --- | --- | --- | --- | --- | --- |
|  | **MIC_99_** | | **GIC_90_** | **SI** | |
| **R =** | ***M. aurum*** | ***M. bovis***  **BCG** | **THP-1** | ***M. aurum*** | ***M. bovis* BCG** |
| **11a** | 500  (1500) | 125  (375) | 250  (977) | 0.5 | 2 |
| **12** | 250  (920) | 250  (920) | 125  (460) | 0.5 | 0.5 |
| **18** | 125  (463) | 31.25  (134) | 31.25  (134) | 0.25 | 1 |
| **20** | 125  (417) | 62.5  (208) | 125  (417) | 1 | 2 |
| **21** | 31.25  (104) | 15.6  (52) | 62.5  (208) | 2 | 4 |
| **28** | > 250  (> 1063) | 250  (1063) | > 500  (2127) | - | > 2 |
| **30** | > 250  (943) | 125  (471) | 500  (1886) | < 2 | 4 |
| **31** | 500  (1694) | 125  (424) | 250  (847) | 0.5 | 2 |
| **32** | 31.25  (106) | 15.6  (53) | 31.25  (106) | 1 | 2 |
| **33** | 62.5  (249) | 62.5  (249) | 125  (498) | 2 | 2 |
| **34** | > 500  (> 1872) | > 500  (> 1872) | > 500  (> 1872) | - | - |
| **35** | > 500  (> 1872) | 62.5  (233) | 62.5  (233) | < 0.125 | 1 |
|  |  |  |  |  |  |

**Chiral HPLC Traces**

| 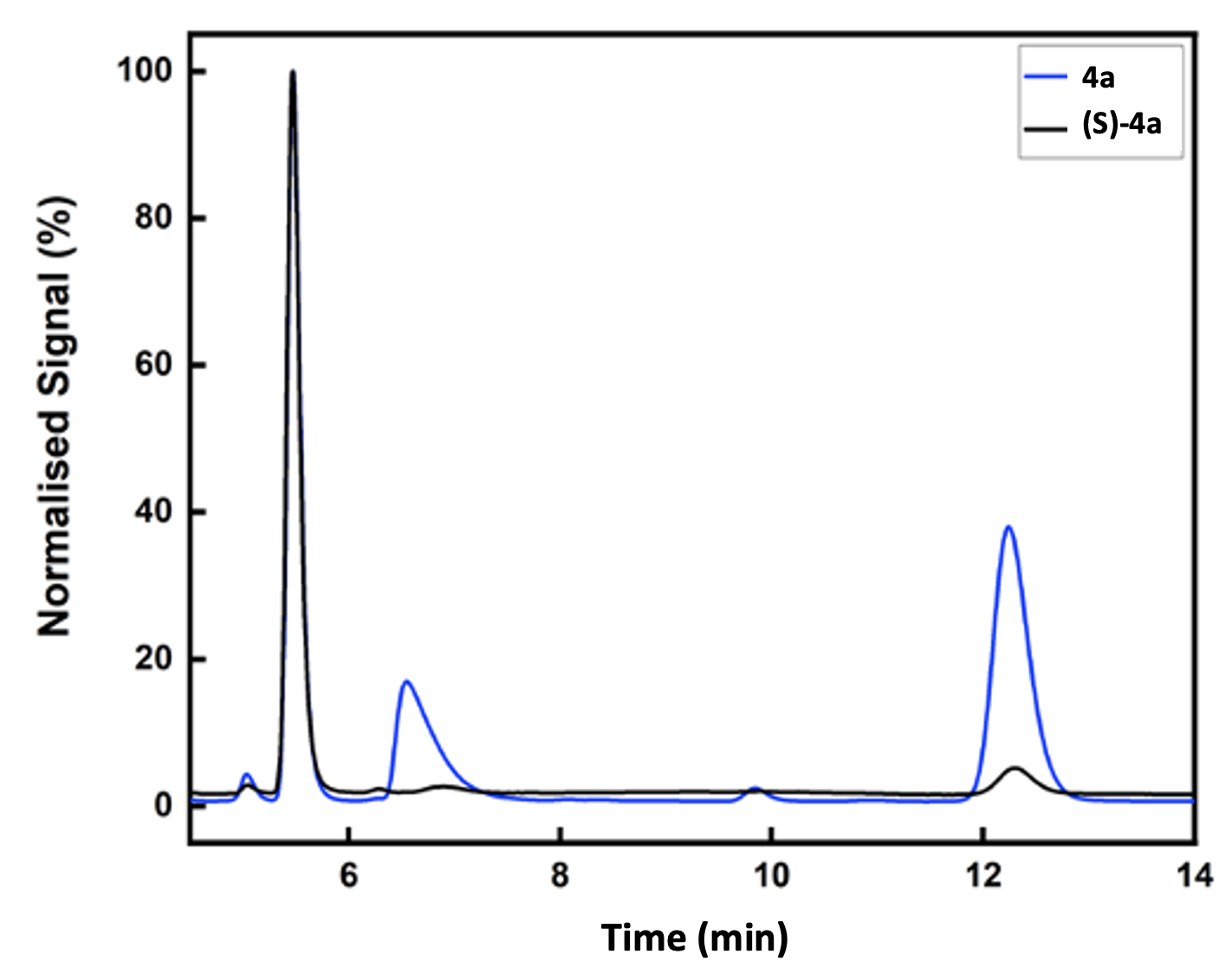 |
| --- |
| **Figure S1.** Chiral HPLC traces for the racemic THIQ **4a** (**blue**) and enantio-enriched (*S*)-THIQ **4a** (**black**), ee = 78%. |

| 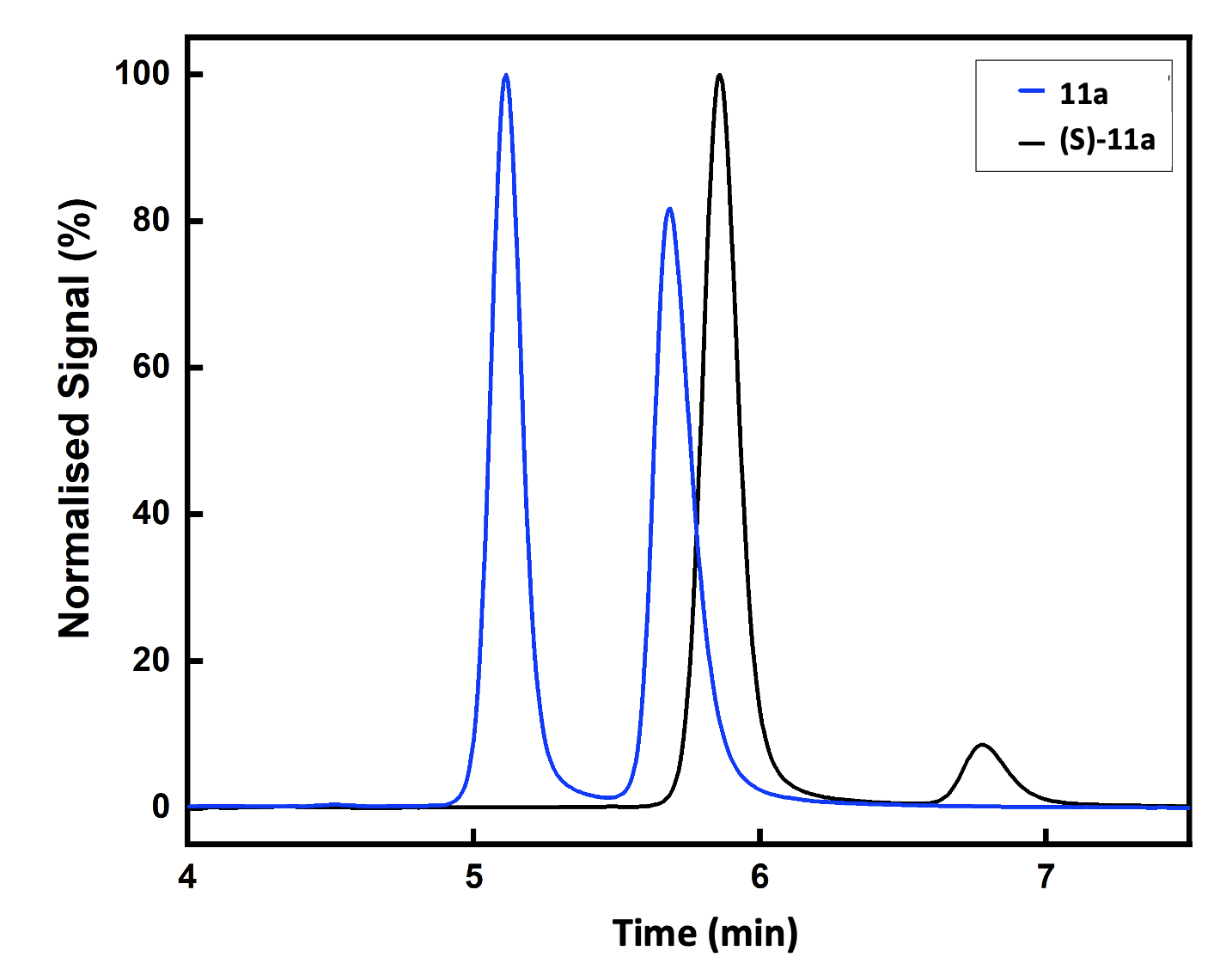 |
| --- |
| **Figure S2.** Chiral HPLC traces for the racemic THIQ (**11a**) and enantio-enriched (*S*)-THIQ **11a**, ee = 79%. |

**Efflux Data**

|  |
| --- |
| **Figure S3. Plot of normalised fluorescence over time for selected compounds in EtBr efflux inhibition assay with *M. aurum*.** Assay performed in technical triplicate. Measurements were taken every minute over 60 mins using a FLUOstar OPTIMA fluorimeter with settings: ex. 544 nm, em. 590 nm, gain 2200, T = 37 ^o^C. |

**Supplementary Methods**

***Middlebrook 7H9 Broth***

Middlebrook 7H9 broth powder (1.88 g, M7H9, BD Difco^TM^), glycerol (50%, 1.6 ml), and Tween 80 (1 ml, 20%) were dissolved in double distilled water (360 ml) and sterilised by autoclaving at 121 ^o^C for 10 minutes. Prior to use, this broth was supplemented by the addition of sterile albumin dextrose catalase (Remel^TM^) to 10% of the total volume of the media.

***Middlebrook 7H10 Agar***

Middlebrook 7H10 agar powder (7.4 g, M7H10, BD Difco^TM^) and glycerol (50%, 4 ml) were dissolved in double distilled water (360 ml) and sterilised by autoclaving 121 ^o^C for 10 minutes. Prior to use, this broth was supplemented by the addition of sterile oleic acid albumin dextrose catalase (BD Difco^TM^) to 10% of the total volume of the media.

***50% Glycerol Solution***

Glycerol (25 ml) was mixed with double distilled water (25 ml). This was filter sterilised, when required.

***Complete RPMI***

An aliquot (50 ml) was removed from a bottle (500 ml) of liquid Roswell Park Memorial Institute (RPMI) 1640 media with L-glutamine (Gibco®) and discarded. The volume removed was then replaced with fetal bovine serum (FBS, Sigma Aldrich, Non-US origin, sterile filtered, heat-inactivated).

***Phosphate Buffer (0.3 M, pH 6)***

A solution of KH_2_PO_4_ (0.3 M, 500 ml) was prepared by dissolving KH_2_PO_4_ (20.4 g, 0.15 mol) in 500 ml of double distilled water. A solution of K_2_HPO­_4_ (0.3 M, 500 ml) was then prepared by dissolving K_2_HPO_4_ (26.1 g, 0.15 M) in 500 ml of double distilled water. The two potassium phosphate solutions were then mixed together until a pH of 6 was achieved.

***HEPES buffer (50 mM, pH 7.5)***

To distilled water (500 ml), was added (4-(2-hydroxyethyl)-piperazineethanesulfonic acid) (HEPES, 11.92 g) and the pH was adjusted to 7.5.

**Growth and Maintenance of Cell Cultures**

***Mycobacterium aurum***

Liquid cultures of *M. aurum* were grown in M7H9 broth. The cultures were grown at 35 ^o^C with shaking at 180 rpm. Cultures of a 10 ml volume were grown in 50 ml centrifuge tubes so that the volume of culture was 20% of the total volume of the vessel. The growth of *M. aurum* cultures was observed by measuring the OD_600_ in a cell density meter (Biochrom WPA Biowave DNA). A sample of the culture was diluted as appropriate in sterile M7H9 medium to ensure that measurements fell within the linear range of the cell density meter (< 0.8). A total volume of 1 ml was used in a disposable cuvette. A measurement of the OD_600_ was then made against a blank sample comprised only of sterile M7H9 medium. When *M. aurum* cultures were passaged, this was performed by transferring a sample of the passaged culture (100 µl) into a new vessel containing sterile M7H9 broth (10 ml). The culture was then grown at 35 ^o^C with shaking at 180 rpm. Solid cultures of *M. aurum* were grown on M7H10 agar. Colonies were observed after 7 days of growth at 35 ^o^C.

***Mycobacterium bovis* BCG**

Liquid cultures of *M. bovis* BCG were grown in a medium comprised of M7H9 broth. The cultures were grown at 37 ^o^C with rolling at 2 rpm. Cultures of a 100 ml volume were grown in 500 ml roller bottles (VWR) so that the volume of culture was 20% of the total volume of the vessel. Alternatively, cultures were also grown at 37 ^o^C with shaking at 180 rpm, when required due to equipment failure. In this case, 10 ml cultures were grown in 50 ml centrifuge tubes so that the volume of culture was 20% of the total volume of the vessel. The growth of *M. bovis* BCG cultures was observed by measuring the OD_600_ in a cell density meter. A sample of the culture was diluted as appropriate in sterile M7H9 medium to ensure that measurements fell within the linear range of the cell density meter (< 0.8). A total volume of 1 ml was used in a disposable cuvette. A measurement of the OD_600_ was then made against a blank sample comprised only of sterile M7H9 medium. When rolling cultures of *M. bovis* BCG cultures were passaged, this was performed by transferring a sample of the passaged culture (1 ml) into a new vessel containing sterile M7H9 broth (100 ml). The culture was then grown at 37 ^o^C with rolling at 2 rpm. When shaking *M. bovis* BCG cultures were passaged, this was performed by transferring a sample of the passaged culture (100 µl) into a new vessel containing sterile M7H9 broth (10 ml). The culture was then grown at 37 ^o^C with shaking at 180 rpm. Solid cultures of *M. bovis* BCG were grown on M7H10 agar. Colonies were observed after 14 days of growth at 37 ^o^C.

**RAW 264.7**

Liquid cultures of RAW 264.7 murine macrophage cells were grown in tissue culture flasks (VWR® Tissue Culture Flask, 50 ml, non-treated, with vent cap, sterilized) in complete RPMI. Cultures were grown in in a CO_2_ incubator with no shaking at 37 ^o^C in a 5% CO_2_ atmosphere. The growth of the cultures was monitored my light microscopy with an inverted light microscope (Zeiss Primovert). RAW 264.7 cells were passaged by first removing the growth media from the tissue culture flask by pipetting. The cells were then washed with PBS (2 x 5 ml) and subsequently detached by incubating with a lidocaine/EDTA solution (10 mM, 5 ml) at RT for 10 min before striking the flask with the palm of the hand nine times. Complete RPMI (5 ml) was then added, and the contents of the flasks were transferred into 50 ml centrifuge tubes and the cells were pelleted by centrifugation (270 x g, 25 ^o^C, 5 min). The supernatant was discarded, and the pellet was washed by resuspension in PBS (5 ml) and subsequent pelleting by centrifugation (270 x g, 25 ^o^C, 5 min). The supernatant was discarded, and the pellet was re-suspended in complete RPMI (5 ml). An aliquot (10 µl) of the suspension was stained with Trypan Blue solution (0.4%, 10 µl) and an aliquot of the stained cells (10 µl) was transferred to a haemocytometer (Neubauer Improved C-Chip) for cell counting under an inverted light microscope. After determination of the cell density, the suspension was divided between as many tissue culture flasks as required and was diluted in the appropriate quantity of complete RPMI to achieve a cell density of 1x10^5^ cells/ml in each of the new cultures, at a total volume of 5 ml.

**THP-1**

Liquid cultures of THP-1 cells were grown in tissue culture flasks in complete RPMI. Cultures were grown with no shaking in a CO_2_ incubator (37 ^o^C in a 5% CO_2_). The growth of the cultures was monitored my light microscopy with an inverted light microscope. THP-1 cells were passaged by first transferring the liquid culture from the tissue culture flask into a centrifuge tube and then pelleting the cells by centrifugation (270 x g, 5 min). The supernatant was discarded, and the pellet was washed by resuspension in PBS (5 ml) and subsequent pelleting by centrifugation (270 x g, 25 ^o^C, 5 min). The pellet was the re-suspended in fresh, complete RPMI (5 ml). An aliquot (10 µl) of the suspension was stained with Trypan Blue solution (0.4%, 10 µl) and an aliquot of the stained cells (10 µl) was transferred to a haemocytometer for cell counting under an inverted light microscope. After determination of the cell density, the suspension was divided between as many tissue culture flasks as required and was diluted in the appropriate quantity of complete RPMI to achieve a cell density of 1x10^5^ cells/ml in each of the new cultures at a total volume of 5 ml.

**Chemical Syntheses**

**General Methods**

All solvents and reagents were obtained commercially and were used as provided. Thin layer chromatography was performed with Machery-Nagel Polygram® silica 60 polyester sheets, 0.2 mm thickness, UV_254_ indicator, 40 x 80 mm plate size. Thin layer chromatography plates were visualised under an UV lamp or were stained with ninhydrin, potassium permanganate, bromocresol green or phosphomolybdic acid solutions. Column chromatography was performed using Merck silica gel 60, (40–63 μm).

^1^H and ^13^C NMR spectroscopic data were obtained with Bruker Avance 300, Bruker Avance III 400, Bruker Avance Neo 500, Bruker, Avance III 600 and Bruker Avance Neo 700 spectrometers. Chemical shifts (δ) are reported in parts per million (ppm) and references to the deuterated solvent or an additional standard where required. The coupling constants (*J*) were measured in Hertz (Hz). Where appropriate, COSY, HMQC and HMBC experiments were carried out to aid signal assignment.

Melting points were determined using an Electrothermal IA9000 melting point apparatus and are uncorrected. Infra-red spectra were obtained using a Bruker Alpha FT-IR spectrometer. LC-MS data was collected using a Waters Acquity Ultra Performance LC system connected to a Waters micromass ZQ mass spectrometer which scanned in the *m*/*z* range from 100 to 1000. High resolution mass spectrometry data was collected on an Agilent 6510 Q TOF mass spectrometer, or an Orbitrap Q Exactive mass spectrometer.

***Chiral analytical HPLC*** was carried out on a Hewlett Packard 1100 HPLC system using either a Supelco Astec Chirobiotic T column (4.6 mm x 250 mm, 5 μm particle size) or a Chiralpak^®^ AD-H column (4.6 mm x 250 mm, 5 μm particle size).

**Preparative HPLC Methods**

***Preparative HPLC Method 1***: HPLC was conducted on a Varian Prostar or Dionex instrument equipped with a UV-visible detector, using a Phenomenex Onyx Monolithic semi-prep C18 column (100 mm length x 10 mm i.d.) at room temperature, eluting with solvents A/B (A: 0.1% v/v solution of trifluoroacetic acid in water, B: 0.1% v/v solution of trifluoroacetic acid in acetonitrile). The gradient employed was 5-40% B in A. The run time was 20 min and the flow rate was 8 mL/min unless otherwise stated. The UV detection was at wavelength 280 nm and 254 nm.

***Preparative HPLC Method 2***: HPLC was conducted on a Varian Prostar or Dionex instrument equipped with a UV-visible detector, using an Acentis C18 column (150 mm length x 21.2 mm i.d., 5 μm) at room temperature, eluting with solvents A/B (A: 0.1% v/v solution of trifluoroacetic acid in water, B: 0.1% v/v solution of trifluoroacetic acid in acetonitrile), according to one of the following gradients. Gradient A: 5-15% B in A; Gradient B: 5-20% B in A; Gradient C: 5-30% B in A; Gradient D: 10-15% B in A; Gradient E: 10-25% B in A; Gradient F: 15% B in A. The run time was 40 min and the flow rate was 7 mL/min unless otherwise stated. The UV detection was at wavelength 280 nm and 254 nm.

***Preparative HPLC Method 3*:** HPLC was conducted on a Varian Prostar instrument equipped with a UV-visible detector, using a Supelco Discovery Bio Wide Pore C18 column (250 mm length x 21.1 mm i.d., 10 μm) at room temperature, eluting with solvents A/B (A: 0.05 M triethylammonium bicarbonate buffer, B: acetonitrile), according to one of the following gradients. Gradient A: 0‑15% B in A; Gradient B: 0- 25% B in A; Gradient C: 0-30% B in A; Gradient D: 0-60% B in A; Gradient E: 0-98% B in A. The flow rate was 8 mL/min. The run time was 25 min unless otherwise stated. The UV detection was at wavelength 254 nm and 214 nm.

***Preparative HPLC Method 4*:** HPLC was conducted on an Agilent Infinity 1260 HPLC instrument, with a UV-visible detector, using a Supleco^®^ Analytical Discovery^®^BIO Wide Pore C18 column (25 cm x 21.2 mm, i.d. 10 µm) at room temperature, eluting with solvents A/B (A: water (+ 0.1% TFA), B: acetonitrile (+0.1% TFA)) according to one of the following gradients: Gradient A: 5–95% B in A; Gradient B: 10–95% B in A; Gradient C: 10–50% B in A; Gradient D: 35–50% B in A; Gradient E: 45–95% B in A; Gradient F: 50–75% B in A; Gradient G: 55–65% B in A; Gradient H: 55–95% B in A. The flow rate was 8 ml/min unless otherwise stated. The run time was 30 mins unless otherwise stated. The UV detection was at wavelengths 254 nm and 214 nm.

***Chiral HPLC Method 1:*** Chiral separation was performed with a Supelco Astec Chirobiotic T column with an isocratic mobile phase of 0.1% TFA and 0.2% triethylamine in MeOH at 1 ml/min at RT. The injections were of 5 µl at a concentration of 1 mg/ml.

***Chiral HPLC Method 2:*** Chiral separation was performed with a Chrialpak^®^ AD-H column (4.6 mm x 250 mm, 5 µm) with an isocratic mobile phase of 0.1% diethylamine and 20% ethanol in *n-*hexane at 1 ml/min at RT. The injections were of 5 µl at a concentration of 1 mg/ml.

**Chemical Synthesis**

**2-(3-Methoxyphenyl)ethan-1-amine**^1,2^

To a solution of 3-methoxyphenylacetonitrile (1.90 ml, 13.6 mmol) in anhydrous THF (20 ml) was added BF_3_.Et_2_O (0.50 ml, 4.00 mmol) followed by BH_3_.SMe_2_ in toluene (2.0 M, 17 ml, 34 mmol). The reaction was heated at reflux for 18 h then cooled on ice and HCl_(aq)_ (10 %, 170 ml) was added over 1 h. The reaction was warmed to RT while stirring for 24 h. The pH of the reaction mixture was increased to 13 by addition of NaOH_(aq)_ (6 M). The aqueous layer was then extracted with CH_2_Cl_2_ (4 x 100 ml). The combined organic layers were dried over Na_2_SO_4_ and the solvent was removed under reduced pressure afford the product as an oil (2.01 g, 97%). ^1^H NMR (600 MHz; CDCl_3_) δ 7.20 (1H, app. t, *J* = 7.8 Hz, **5-H**), 6.78–6.73 (3H, m, **2-**, **4‑** & **6-H**), 3.77 (3H, s, **OCH_3_**), 2.92 (2H, t, *J* = 6.9 Hz, **C*H*_2_N**), 2.70 (2H, t, *J* = 6.9 Hz, **C*H*_2_CH_2_N**), 1.78 (2H, broad s, **N*H*_2_**); ^13^C NMR (151 MHz; CDCl_3_) δ 161.4, 141.9, 130.6, 122.0, 115.5, 112.8, 55.6, 43.7, 39.1; *m/z* [ES+] 152 ([M + H]^+^, 100%).

**2-(4-Methoxyphenyl)ethan-1-amine**^3^

To a stirred solution of 4-methoxyphenylacetonitrile (1.50 g, 10.2 mmol) in THF (21 ml) at 0 ^o^C, was added a 1 M borane tetrahydrofuran complex solution in THF (25.5 ml, 25.5 mmol) and the reaction mixture was allowed to gradually warm to RT. After 24 h, the reaction was cooled to 0 ^o^C and methanol (30 ml) was added dropwise. The resulting clear solution was stirred at RT overnight and concentrated *in vacuo*. The residue was co-evaporated with methanol (3 x 20 mL), and the crude product was purified by column chromatography (CH_2_Cl_2_/CH_3_OH(+1% Et_3_N), 85:15) to give the product as a pale yellow oil (1.17 g, 76%). R*f* = 0.14 (CH_2_Cl_2_/CH_3_OH(+1% Et_3_N), 4:1); ^1^H NMR (500 MHz; CDCl_3_) δ 7.11 (2H, d, *J* = 8.5 Hz, **2 x 2-H**), 6.84 (2H, d, *J* = 8.5 Hz, **2 x 3-H**), 3.83 (3H, s, **OCH_3_**), 2.93 (2H, t, *J* = 7.0 Hz, **CH_2_N**), 2.68 (2H, t, *J* = 7.0 Hz, **C*H*_2_CH_2_N**), 1.26 (2H, br s, **NH_2_**); ^13^C NMR (125 MHz; CDCl_3_) δ 158.1, 132.0, 129.8, 114.0, 55.3, 43.8, 39.3.

**2-(3,5-Dimethoxyphenyl)ethan-1-amine**^2,4^

To a solution of (3,5-dimethoxyphenyl)acetonitrile (1.50 g, 8.47 mmol) in anhydrous THF (15 ml) under anhydrous conditions, was added BF_3_.Et_2_O (0.70 ml, 5.67 mmol), followed by B_2_H_6_.SMe_2_ in toluene (2 M, 10.6 ml, 21.1 mmol), dropwise. The reaction mixture was heated at reflux for 18 h. The reaction mixture was cooled on ice prior to the addition of HCl_(aq)_ (10%, 170 ml) dropwise over 1 h. The reaction was left to warm to RT while stirring for 24 h. The pH of the reaction mixture was then increased to 14 by addition of NaOH_(aq)_ and the aqueous layer was extracted with CH_2_Cl_2_ (4 x 100 ml). The combined organic layers were dried (Na_2_SO_4_) and the solvent removed under reduced pressure to afford the product as an oil (1.10 g, 72%). IR (neat) ν_max_/cm^-1^: 3361, 2937, 2937, 1593; ^1^H NMR (600 MHz; CDCl_3_) δ 6.34 (2H, d, *J* = 2.4 Hz, **2-H**), 6.31 (1H, t, *J* = 2.4 Hz, **4-H**), 3.76 (6H, s, **2 x** **OC*H*_3_**), 2.94 (2H, t, *J* = 6.9 Hz, **C*H*_2_N**), 2.67 (2H, t, *J* = 6.9 Hz, **C*H*_2_CH_2_N**); ^13^C NMR (151 MHz; CDCl_3_) δ 160.9, 142.3, 107.0, 98.1, 55.3, 43.4, 40.5; *m/z* [ES+] 182 ([M + H]^+^, 100%).

**5-(2-Aminoethyl)benzene-1,3-diol.HBr**^5^

To a solution of 2-(3,5-dimethoxyphenyl)ethan-1-amine (300 mg, 1.65 mmol) in anhydrous CH_2_Cl_2_ under argon, was added BBr_3_ in CH_2_Cl_2_ (1 M, 5.00 ml, 5.00 mmol). The reaction was heated at reflux for 18 h. It was cooled to 0 ^o^C and quenched by the dropwise addition of MeOH (5 ml) and the solvent was removed under reduced pressure. The crude product was repeatedly azeotroped with MeOH until no fumes were evolve upon further addition of MeOH. This afforded the hydrobromide salt of the product as an oil/solid (293 mg, 76%). ^1^H NMR (700 MHz; CD_3_OD) δ 6.23 (2H, d, *J* = 2.1 Hz, **2-H**), 6.19 (1H, t, *J* = 2.1 Hz, **4-H**), 3.14 (2H, t, *J* = 7.7 Hz, **C*H*_2_N**) 2.81 (2H, t, *J* = 7.7 Hz, **C*H*_2_CH_2_N**); ^13^C NMR (176 MHz; CD_3_OD) δ 159.9, 139.8, 108.0, 102.3, 41.7, 34.4; *m/z* [ES+] 154 ([M + H]^+^, 100%).

**2-(2-Bromo-5-methoxyphenyl)ethan-1-amine**^6^

A solution of 2-(3-methoxyphenyl)ethan-1-amine (1.50 g, 9.92 mmol) in glacial acetic acid (21 ml) was treated with a solution of bromine (0.56 ml, 10.9 mmol) in acetic acid (8.50 ml) over 45 min at -15 ^o^C. After stirring for a further 5 min, the resulting orange solution was treated with Et_2_O (80 ml) and cooled to 0 ^o^C. Water was added (80 ml) and the aqueous phase was then separated. The organic phase was extracted with water (2 x 80 ml). The combined aqueous extracts were washed with Et_2_O (2 x 50 ml), the aqueous phase was adjusted to pH 10 by addition of 2 M NaOH and then extracted with CH_2_Cl_2_ (3 x 150 ml). The combined organic extracts were dried (MgSO_4_) and solvent removed *in vacuo* to yield the product as a pale yellow oil (2.00 g, 88%), which was used without further purification. IR (CDCl_3_ cast) ν_max_/cm^-1^: 2934, 2836, 1594, 1571; ^1^H NMR (500 MHz; CDCl_3_) δ 7.42 (1H, d, *J* = 8.5 Hz, **3-H**), 6.78 (1H, d, *J* = 3.0 Hz, **6-H**), 6.64 (1H, dd, *J* = 8.5 and 3.0 Hz, **4-H**), 3.77 (3H, s, **OCH_3_**), 2.96 (2H, t, *J* = 7.0 Hz, **CH_2_N**), 2.84 (2H, t, *J* = 7.0 Hz, **C*H*_2_CH_2_N**), 1.25 (2H, br s, **NH_2_**); ^13^C NMR (125 MHz; CDCl_3_) δ 159.0, 140.2, 133.5, 116.7, 115.2, 113.5, 55.5, 42.2, 40.6.

**2-(2-Bromo-3,5-dimethoxyphenyl)ethan-1-amine**^7^

A solution of 2-(3,5-dimethoxyphenyl)ethan-1-amine (245 mg, 1.35 mmol) in glacial acetic acid (2.9 ml) was treated with a solution of bromine (0.08 ml, 1.48 mmol) in acetic acid (1.16 ml), dropwise at -15 ^o^C. After stirring for 5 min, the resulting orange solution was treated with Et_2_O (80 ml) and cooled to 0 ^o^C. Water (10 ml) was added and the aqueous phase separated and the organic phase was extracted with water (2 x 10 ml). The combined aqueous extracts were washed with Et_2_O (2 x 10 ml), the aqueous phase was adjusted to pH 10 by addition of 2 M NaOH and extracted with CH_2_Cl_2_ (3 x 20 ml). The combined organic extracts were dried (MgSO_4_) and solvent removed *in vacuo* to afford an oil which was purified by column chromatography (CH_2_Cl_2_/CH_3_OH(+1% Et_3_N), 9:1) to afford the product as a pale yellow oil (150 mg, 34%). R*f* = 0.34 (CH_2_Cl_2_/MeOH(+1% Et_3_N), 4:1); IR (CDCl_3_ cast) ν_max_/cm^-1^: 2938, 2844, 1584; ^1^H NMR (500 MHz; CDCl_3_) δ 6.43 (1H, d, *J* = 2.5 Hz, **Ar-H**), 6.37 (1H, d, *J* = 2.5 Hz, **Ar-H**), 3.86 (3H, s, **OCH_3_**), 3.80 (3H, s, **OCH_3_**), 3.08-2.90 (4H, m, **C*H*_2_C*H*_2_N**), 2.01 (2H, br s, **NH_2_**); ^13^C NMR (125 MHz; CDCl_3_) δ 159.6, 156.9, 140.8, 107.3, 105.0, 97.9, 56.4, 55.6, 41.9, 40.4; *m/z* [ES+] 262 ([^81^M + H]^+^, 30%), 260 ([^79^M + H]^+^, 35%), 245 ([^81^MH - OH]^+^, 100%); *m/z* [HRMS, ES+] found [M + H]^+^ 260.0264, C_10_H_15_O_2_N^79^Br requires 260.0286.

**2-(2-Bromo-4,5-dimethoxyphenyl)ethan-1-amine**^8^

A solution of 2-(3,4-dimethoxyphenyl)ethan-1-amine (1.80 g, 9.93 mmol) in glacial acetic acid (21 ml) was treated with a solution of bromine (0.56 ml, 10.9 mmol) in acetic acid (8.50 ml) over 45 min at -15 ^o^C. After stirring for 5 min, the resulting orange solution was treated with Et_2_O (80 ml) and cooled to 0 ^o^C. Water (80 ml) was added and the aqueous phase separated. The organic phase was extracted with water (2 x 80 ml) and the combined aqueous extracts were washed with Et_2_O (2 x 50 ml). The aqueous phase was adjusted to pH 10 by addition of 2 M NaOH and extracted with CH_2_Cl_2_ (3 x 150 ml). The combined organic extracts were dried (MgSO_4_) and solvent removed *in vacuo* to afford an oil which was purified by column chromatography (CH_2_Cl_2_/CH_3_OH(+1% Et_3_N), 9:1) to yield the product as a yellow oil (1.12 g, 43%). R*f* = 0.33 (CH_2_Cl_2_/CH_3_OH(+1% Et_3_N), 4:1); IR (CDCl_3_ cast) ν_max_/cm^-1^: 2934, 2844, 1617, 1586, 1507; ^1^H NMR (500 MHz; CDCl_3_) δ 6.96 (1H, s, **3-H**), 6.74 (1H, s, **6-H**), 3.85 (3H, s, **OCH_3_**), 3.84 (3H, s, **OCH_3_**), 2.95 (2H, t, *J* = 7.0 Hz, **CH_2_N**), 2.82 (2H, t, *J* = 7.0 Hz, **C*H*_2_CH_2_N**), 1.43 (2H, br s, **NH_2_**); ^13^C NMR (125 MHz; CDCl_3_) δ 148.4, 148.2, 131.1, 115.8, 114.4, 113.5, 56.2, 42.4, 39.9; *m/z* [CI] 262 ([^81^M + H]^+^, 90%), 260 ([^79^M + H]^+^, 100%); *m/z* [HRMS, ES+] found [M + H]^+^ 260.0283, C_10_H_15_O_2_N^79^Br requires 260.0286.

**2-(2-Bromo-5-hydroxyphenyl)ethan-1-amine hydrobromide**^9^

To a stirred solution of 2-(2-bromo-5-methoxyphenyl)ethan-1-amine (2.00 g, 8.69 mmol) in CH_2_Cl_2_ (40 ml) was added a solution of 1 M boron tribromide in CH_2_Cl_2_ (19.1 ml, 19.1 mmol) at -78 ^o^C. The reaction was stirred at RT for 24 h. The reaction was then cooled to 0 ^o^C and quenched by the addition of methanol (80 ml), the solution was stirred at RT for 3 h and then concentrated *in vacuo*. Further methanol (20 ml) was added to the oil, and the solvent evaporated. This was repeated until no white fumes were observed upon addition of methanol, to give the hydrobromide salt of the product as a pale brown solid (2.40 g, 93%). Mp. 162-172 ^o^C (decomposed); IR (neat) ν_max_/cm^-1^: 3348, 2924, 1588; ^1^H NMR (500 MHz; CD_3_OD) δ 7.36 (1H, d, *J* = 8.5 Hz, **3-H**), 6.79 (1H, d, *J* = 3.0 Hz, **6-H**), 6.65 (1H, dd, *J* = 8.5 and 3.0 Hz, **4-H**), 3.20-3.11 (2H, m, **CH_2_N**), 3.05-2.97 (2H, m, **C*H*_2_CH_2_N**); ^13^C NMR (125 MHz; CD_3_OD) δ 158.8, 138.0, 134.9, 119.0, 117.4, 113.7, 40.4, 35.0.

**2-(2-Bromo-4,5-dihydroxyphenyl)ethan-1-amine hydrobromide**^10^

To a stirred solution of 2-(2-bromo-4,5-dimethoxyphenyl)ethan-1-amine (655 mg, 2.52 mmol) in CH_2_Cl_2_ (12 ml) was added a solution of 1 M boron tribromide in CH_2_Cl_2_ (7.55 ml, 7.55 mmol) at ‑78 ^o^C. The reaction was warmed to RT and stirred for 24 h, cooled to 0 ^o^C and quenched by the addition of methanol (35 ml). The solution was stirred at RT for 3 h and then concentrated *in vacuo*. Further methanol (10 ml) was added, and solvent evaporated. This was repeated until no white fumes were observed upon addition of methanol, to afford the hydrobromide salt of the product as a pale brown solid (716 mg, 91%). ^1^H NMR (600 MHz; CD_3_OD) δ 6.97 (1H, s, **3-H**), 6.77 (1H, s, **6-H**), 3.12-3.07 (2H, m, **CH_2_N**), 2.96-2.90 (2H, m, **C*H*_2_CH_2_N**); ^13^C NMR (150 MHz; CD_3_OD) δ 147.0, 146.7, 127.7, 120.4, 118.4, 113.3, 40.7, 34.3; *m/z* [ES+] 234 ([^81^M + H]^+^, 95%), 232 ([^79^BrM + H]^+^, 100%); *m/z* [HRMS, ES+] found [M + H]^+^ 231.9978, C_8_H_11_NO_2_^79^Br requires 231.9973.

**2-(2-Bromo-4,5-dihydroxyphenyl)ethan-1-amine trifluoroacetic acid salt**

To a stirred solution of 2-(2-bromo-3,5-dimethoxyphenyl)ethan-1-amine (140 mg, 0.538 mmol) in CH_2_Cl_2_ (2.5 ml) was added a solution of 1 M boron tribromide in CH_2_Cl_2_ (1.61 ml, 1.61 mmol) at ‑78 ^o^C. The reaction was warmed to RT and stirred for 24 h, then cooled to 0 ^o^C and quenched by the addition of methanol (7 ml). The solution was stirred at RT for 3 h and concentrated *in vacuo* to give a brown oil. Further methanol (2 ml) was added to the oil, and solvent evaporated. This was repeated until no white fumes were observed upon addition of methanol, to afford an oil which was purified by prep-HPLC (Method 2, Gradient B, run time: 30 min, Rt = 14.6 min) to yield the TFA salt as a yellow oil (110 mg, 59%). ^1^H NMR (300 MHz; CD_3_OD) δ 6.36 (1H, d, *J* = 2.5 Hz, **Ar‑H**), 6.32 (1H, d, *J* = 2.5 Hz, **Ar-H**), 3.28-3.08 (2H, m, **CH_2_N**), 3.05-2.95 (2H, m, **C*H*_2_CH_2_N**); ^13^C NMR (150 MHz; CD_3_OD) δ 159.0, 156.7, 138.6, 110.1, 103.4, 102.4, 40.4, 35.3; *m/z* [CI] 234 ([^81^M + H]^+^, 100%), 232 ([^79^M + H]^+^, 95%); *m/z* [HRMS, ES+] found [M + H]^+^ 231.9973, C_8_H_11_NO_2_^79^Br requires 231.9973.

**2-(*m*-Tolyl)acetaldehyde**^11^

To a solution of 3-methylphenethyl alcohol (500 mg, 3.59 mmol) in DMSO/CH_2_Cl_2_ 1:1 (14 ml) was added DIPEA (1.88 ml, 10.8 mmol) at -15 ^o^C, followed by a solution of SO_3_.pyridine (1.43 g, 8.98 mmol) in DMSO (7 ml) over 15 min. The reaction mixture was stirred for 1 h at -15 ^o^C. The reaction was quenched by the addition of ice-cold water (40 ml). The aqueous layer was extracted with CH_2_Cl_2_ (3 x 40 ml). The combined organic extracts were washed with ice cold water (5 x 25 ml), brine (25 ml), dried (Na_2_SO_4_) and solvent evaporated to give the crude product, which was purified by column chromatography (hexane/EtOAc, 95:5) to give the product as a pale yellow oil (294 mg, 60%). R*f* = 0.68 (hexane/EtOAc, 2:1); ^1^H NMR (600 MHz; CDCl_3_) δ 9.74 (1H, t, *J* = 2.5 Hz, **CHO**), 7.26 (1H, t, *J* = 7.5 Hz, **Ar-H**), 7.12 (1H, d, *J* = 7.5 Hz, **Ar-H**), 7.05-7.00 (2H, m, 2 x **Ar-H**), 3.65 (2H, d, *J* = 2.5 Hz, **C*H*_2_CHO**), 2.36 (3H, s, **CH_3_**); ^13^C NMR (150 MHz; CDCl_3_) δ 199.8, 138.9, 131.8, 130.8, 129.0, 128.3, 126.8, 50.7, 21.5; *m/z* [CI] 135 ([M + H]^+^, 100%); *m/z* [HRMS, ES+] found [M + H]^+^ 135.0804, C_9_H_11_O, requires 135.0810

**3,4-(Methylenedioxy)phenethyl alcohol**^12^

A solution of 3,4-(methylenedioxy)phenylacetic acid (2.00 g, 11.1 mmol) in THF (50 ml) was added slowly to a solution of lithium aluminium hydride in THF (1 M, 24.4 ml, 24.4 mmol) at 0 ^o^C and stirred at RT for 5 h. The reaction mixture was cooled to 0 ^o^C, quenched with water (25 ml), EtOAc (100 ml) was added and the mixture was filtered to remove the solids present, which were washed with EtOAc (3 x 50 ml). The combined organic extracts were washed with brine (40 ml), dried (MgSO_4_) and solvent removed *in vacuo* to yield the product as a pale yellow oil (1.77 g, 96%) which was used without further purification. R*f* = 0.2 (hexane/EtOAc, 2:1); ^1^H NMR (300 MHz; CDCl_3_) δ 6.81-6.65 (3H, m, **3 x Ar-H**), 5.93 (2H, s, **OCH_2_O**), 3.81 (2H, t, *J* = 6.5 Hz, **C*H*_2_OH**), 2.78 (2H, t, *J* = 6.5 Hz, **C*H*_2_CH_2_OH**); *m/z* [CI] 166 (M^+^, 20%), 149 (M^+^ - OH, 100%); *m/z* [HRMS ES+] found [M + H]^+^ 166.0623, C_9_H_10_O_3_, requires 166.0630.

**3,4-(Methylenedioxy)phenylacetaldehyde**^13^

To a solution 3,4-(methylenedioxy)phenethyl alcohol (500 mg, 3.01 mmol) in DMSO/CH_2_Cl_2_ 1:1 (12 ml) was added DIPEA (1.58 ml, 9.07 mmol) at -15 ^o^C, followed by a solution of SO_3_.pyridine (1.20 g, 7.54 mmol) in DMSO (5.9 ml) over 15 min. The reaction mixture was stirred at -15 ^o^C for 1 h. The reaction was quenched by the addition of ice-cold water (40 ml) and the aqueous layer extracted with CH_2_Cl_2_ (3 x 40 ml). The combined organic extracts were washed with ice cold water (5 x 25 ml), brine (25 ml), dried (Na_2_SO_4_) and solvent evaporated to give the crude product, which was purified by column chromatography (hexane/EtOAc, 85:15) to give the product as a pale yellow oil (254 mg, 51%). R*f* = 0.50 (hexane/EtOAc, 2:1); ^1^H NMR (600 MHz; CDCl_3_) δ 9.70 (1H, s, **CHO**), 6.80 (1H, d, *J* = 8.0 Hz, **5‑H**), 6.69 (1H, s, **Ar-H**), 6.66 (1H, d, *J* = 8.0 Hz, **6-H**), 5.97 (2H, s, **OCH_2_O**), 3.60 (2H, s, **CH_2_CHO**); ^13^C NMR (150 MHz; CDCl_3_) δ 199.5, 148.3, 147.1, 125.4, 123.0, 110.1, 109.0, 101.4, 50.3; *m/z* [CI] 165 ([M + H]^+^, 100%); *m/z* [HRMS, ES+] found [M + H]^+^ 165.0550, C_9_H_9_O_3_ requires 165.0552.

**1-Ethyl-1,2,3,4-tetrahydroisoquinoline-6,7-diol** (**1a**)^14^ **and 1-Ethyl-1,2,3,4- tetrahydroisoquinoline-7,8-diol** (**1b**)^15^

Propionaldehyde (92.0 μl, 1.27 mmol) was added to dopamine hydrochloride (200 mg, 1.05 mmol) in potassium phosphate buffer (10 ml, 0.1 M, pH 6) and acetonitrile (10 ml) under argon. The solution was stirred at 50 ^o^C for 18 h. The reaction was concentrated under vacuum, CH_2_Cl_2_/CH_3_OH (1:1, 20 ml) was added, and the resulting suspension filtered to remove the solid. The filtrate was evaporated to dryness under vacuum to give the crude products which were purified by preparative HPLC (Method 1, run time: 10 min, flow rate: 5 ml/min). The appropriate fractions were combined, concentrated and co-evaporated with methanol (x 3) to give **1a** as a pale brown oil (Rt = 1.5 min, 156 mg, 77%) and **1b** as a pale yellow oil (Rt = 2.6 min, 8.3 mg, 4%).

*Major regioisomer* **1a**: ^1^H NMR (500 MHz; CD_3_OD) δ 6.67 (1H, s**, 8-H**), 6.63 (1H, s, **5‑H**), 4.33-4.29 (1H, m, **1‑CH**), 3.56-3.29 (2H, m, **3-CH_2_**), 3.05-2.87 (2H, m, **4-CH_2_**), 2.17-1.88 (2H, m, **1-C*H*CH_2_**), 1.12 (3H, t, *J*= 7.5 Hz, **CH_3_**); ^13^C NMR (125 MHz; CD_3_OD) δ 145.2, 144.4, 122.5, 122.2, 114.7, 112.4, 56.4, 39.6, 26.4, 24.2, 8.5; *m/z* [CI] 194 ([M + H]^+^, 10%); *m/z* [HRMS, ES+] found [M + H]^+^ 194.1179, C_11_H_16_O_2_N requires 194.1181.

*Minor regioisomer* **1b**: ^1^H NMR (500 MHz; CD_3_OD) δ 6.73 (1H, d, *J* = 8.0 Hz, **6-H**), 6.55 (1H, d, *J* = 8.0 Hz, **5-H**), 4.57-4.51 (1H, m, **1-H**), 3.51-3.27 (2H, m, **3-H_2_**), 3.04-2.89 (2H, m, **4-H_2_**), 2.25-1-81 (2H, m, **1-CHCH_2_**), 1.11 (3H, t, *J* = 7.5 Hz, **CH_3_**); ^13^C NMR (125 MHz; CD_3_OD) δ 144.6, 143.4, 123.3, 121.1, 120.5, 116.0, 54.8, 38.8, 26.1, 25.5, 10.7; *m/z* [CI] 194 ([M + H]^+^, 30%), 164 ([MH - C_2_H_6_]^+^, 100%); *m/z* [HRMS, ES+] found [M + H]^+^ 194.1180, C_11_H_16_O_2_N, requires 194.1181.

**1-Propyl-1,2,3,4-tetrahydroisoquinoline-6,7-diol** (**2a**)^14^ **and 1-Propyl- 1,2,3,4-tetrahydroisoquinoline-7,8-diol** (**2b**)

Butyraldehyde (114 μl, 1.27 mmol) was added to dopamine hydrochloride (200 mg, 1.05 mmol) in potassium phosphate buffer (10 ml, 0.1 M, pH 6) and acetonitrile (10 ml) under argon. The solution was stirred at 50 ^o^C for 18 h. The reaction was concentrated under vacuum, CH_2_Cl_2_/CH_3_OH (1:1, 20 ml) added, and the resulting suspension filtered to remove the solid. The filtrate was evaporated to dryness under vacuum to give the crude products which were purified by preparative HPLC (Method 2, Gradient A) to give **2a** as a pale brown solid (Rt = 17.8 min, 99.0 mg, 45%) and **2b** as a pale yellow oil (Rt = 30.4 min, 7.7 mg, 3%).

*Major regioisomer* **2a**: Mp. 193-195 ^o^C; ^1^H NMR (600 MHz; CD_3_OD) δ 6.65 (1H, s, **8-H**), 6.61 (1H, s, **5-H**), 4.37-4.32 (1H, m, **1-H**), 3.54-3.28 (2H, m, **3-H_2_**), 3.02-2.87 (2H, m, **4-H_2_**), 2.05-1.81 (2H, m, **C*H*_2_CH_2_CH_3_**), 1.59-1.47 (2H, m, **C*H*_2_CH_3_**), 1.05 (3H, t, *J* = 7.5 Hz, **CH_3_**); ^13^C NMR (150 MHz; CD_3_OD) δ 146.7, 145.9, 124.2, 123.5, 116.2, 113.8, 56.4, 40.9, 37.3, 25.7, 19.8, 14.1; *m/z* [CI] 208 ([M + H]^+^, 50%), 131 ([MH - C_3_H_9_O_2_]^+^, 40%); *m/z* [HRMS, ES+] found [M + H]^+^ 208.1331 C_12_H_18_NO_2_, requires 208.1338.

*Minor regioisomer* **2b**: IR (neat) ν_max_/cm^-1^: 3330 br, 3047, 2813, 1665, 1633, 1586, 1506; ^1^H NMR (600 MHz; CD_3_OD) δ 6.74 (1H, d, *J* = 8.0 Hz, **6-H**), 6.55 (1H, d, *J* = 8.0 Hz, **5-H**), 4.63 (1H, dd, *J* = 9.5 and 3.5 Hz, **1-H**), 3.51-3.28 (2H, m, **3-H_2_**), 3.04-2.92 (2H, m, **4-H_2_**), 2.13-1.80 (2H, m, **C*H*_2_CH_2_CH_3_**), 1.65-1.47 (2H, m, **C*H*_2_CH_3_**), 1.03 (3H, t, *J* = 7.5 Hz, **CH_3_**); ^13^C NMR (150 MHz; CD_3_OD) δ 144.7, 143.3, 123.2, 121.3, 120.4, 116.0, 53.1, 38.7, 35.2, 25.5, 20.3, 14.0; *m/z* [CI] 208 ([M + H]^+^, 45%), 164 ([MH - C_3_H_8_]^+^, 40%); *m/z* [HRMS, ESI+] found 208.1331, C_12_H_18_NO_2_ requires 208.1338.

**1-Butyl-1,2,3,4-tetrahydroisoquinoline-6,7-diol** (**3a**)^16^ **and 1-Butyl-1,2,3,4- tetrahydroisoquinoline-7,8-diol** (**3b**)

Valeraldehyde (135 μl, 1.27 mmol) was added to dopamine hydrochloride (200 mg, 1.05 mmol) in potassium phosphate buffer (10 ml, 0.1 M, pH 6) and acetonitrile (10 ml) under argon. The solution was stirred at 50 ^o^C for 18 h. The reaction was concentrated under vacuum, CH_2_Cl_2_/CH_3_OH (1:1, 20 ml) added, and the resulting suspension filtered to remove the solid. The filtrate was evaporated to dryness under vacuum to afford the crude products which were purified by preparative HPLC (Method 2, Gradient A, run time: 50 min) to give **3a** as a pale brown solid (Rt = 22.5 min, 193 mg, 83%) and **3b** as a pale yellow oil (Rt = 31.7 min, 11.4 mg, 5%).

*Major regioisomer* **3a**: Mp. 185-186 ^o^C; ^1^H NMR (600 MHz; CD_3_OD) δ 6.65 (1H, s, **8-H**), 6.61 (1H, s, **5-H**), 4.36-4.32 (1H, m, **1-H**), 3.54-3.28 (2H, m, **3-H_2_**), 3.03-2.87 (2H, m, **4-H_2_**), 2.09-1.84 (2H, m, **CHC*H*_2_**), 1.53-1.39 (4H, m, **C*H*_2_C*H*_2_CH_3_**), 0.99 (3H, t, *J* = 7.0 Hz, **CH_3_**); ^13^C NMR (150 MHz; CD_3_OD) δ 146.7, 145.9, 124.2, 123.6, 116.2, 113.8, 56.6, 41.0, 34.9, 28.6, 25.7, 23.6, 14.2; *m/z* [CI] 222 ([M + H]^+^, 80%), 164 ([MH - C_4_H_10_]^+^, 85%), 131 ([MH - C_4_H_11_O_2_]^+^, 40%); *m/z* [HRMS, ES+] found [M + H]^+^ 222.1490, C_13_H_20_NO_2_ requires 222.1494.

*Minor regioisomer* **3b**: IR (neat) ν_max_/cm^-1^: 3047 br, 2962, 2875, 1664, 1625, 1586, 1500; ^1^H NMR (600 MHz; CD_3_OD) δ 6.74 (1H, d, *J* = 8.0 Hz, **6-H**), 6.55 (1H, d, *J* = 8.0 Hz, **5-H**), 4.62 (1H, dd, *J* = 9.5 and 3.5 Hz, **1-H**), 3.51-3.28 (2H, m, **3-H_2_**), 3.04-2.92 (2H, m, **4-H_2_**), 2.19-1.80 (2H, m, **CHC*H*_2_**), 1.59-1.37 (4H, m, **C*H*_2_C*H*_2_CH_3_**), 0.98 (3H, t, *J* = 7.5 Hz, **CH_3_**); ^13^C NMR (150 MHz; CD_3_OD) δ 144.7, 143.3, 123.3, 121.3, 120.4, 116.0, 53.8, 38.7, 32.7, 29.1, 25.5, 23.3, 14.2; *m/z* [CI] 222 ([M + H]^+^, 95%), 164 ([MH - C_4_H_10_]^+^, 100%); *m/z* [HRMS, ES+] found [M + H]^+^ 222.1488, C_13_H_20_NO_2_ requires 222.1494.

**1-Pentyl-1,2,3,4-tetrahydroisoquinoline-6,7-diol** (**4a**)^14^ **and 1-Pentyl-1,2,3,4- tetrahydroisoquinoline-7,8-diol** (**4b**)

Hexanal (77.2 μl, 0.632 mmol) was added to dopamine hydrochloride (100 mg, 0.527 mmol) in potassium phosphate buffer (5 ml, 0.1 M, pH 6) and acetonitrile (5 ml) under argon. The solution was stirred at 50 ^o^C for 18 h. The reaction was concentrated under vacuum, CH_2_Cl_2_/CH_3_OH (1:1, 10 ml) added, and the resulting suspension filtered to remove the solid. The filtrate was evaporated to dryness under vacuum and the crude products purified by preparative HPLC (Method 1, run time: 30 min) to give **4a** as a white solid (Rt = 17.2 min, 92.7 mg, 75%) and **4b** as an orange oil (Rt = 22.6 min, 11.3 mg, 9%).

*Major regioisomer* **4a**: Mp. 166-168 ^o^C (lit^14^ 164-170 ^o^C, Et_2_O); ^1^H NMR (500 MHz; CD_3_OD) δ 6.64 (1H, s, **8-H**), 6.60 (1H, s, **5-H**), 4.36-4.30 (1H, m, **1-H**), 3.51- 3.26 (2H, m, **3-H_2_**), 3.02-2.84 (2H, m, **4-H_2_**), 2.08-1.81 (2H, m, **CHC*H*_2_**), 1.56- 1.33 (6H, m, **3 x CH_2_**), 0.94 (3H, t, *J* = 6.5 Hz, **CH_3_**); ^13^C NMR (125 MHz; CD_3_OD) δ 146.7, 145.9, 124.3, 123.6, 116.2, 113.9, 56.7, 41.0, 35.1, 32.7, 26.1, 25.7, 23.5, 14.3; *m/z* [CI] 236 ([M + H]^+^, 85%), 164 ([MH - C_5_H_12_]^+^, 100%); *m/z* [HRMS, ES+] found [M + H]^+^ 236.1644, C_14_H_22_O_2_N requires 236.1645.

*Minor regioisomer* **4b**: IR (neat) ν_max_/cm^-1^: 3039 br, 2961, 2932, 1662, 1617, 1578, 1501; ^1^H NMR (600 MHz; CD_3_OD) δ 6.74 (1H, d, *J* = 8.0 Hz, **6-H**), 6.55 (1H, d, *J* = 8.0 Hz, **5-H**), 4.62 (1H, dd, *J* = 9.5 and 3.5 Hz, **1-H**), 3.51-3.28 (2H, m, **3-H_2_**), 3.04-2.92 (2H, m, **4-H_2_**), 2.17-1.80 (2H, m, **CHC*H*_2_**), 1.62-1.47 (2H, m, **CHCH_2_C*H*_2_**), 1.45-1.35 (4H, m, **C*H*_2_C*H*_2_CH_3_**), 0.94 (3H, t, *J* = 7.0 Hz, **CH_3_**); ^13^C NMR (150 MHz; CD_3_OD) δ 144.6, 143.3, 123.3, 121.3, 120.4, 116.0, 53.4, 38.7, 33.0, 32.5, 26.7, 25.5, 23.5, 14.3; *m/z* [CI] 236 ([M + H]^+^, 35%), 164 ([MH - C_5_H_10_]^+^, 100%); *m/z* [HRMS, ES+] found [M + H]^+^ 236.1644, C_14_H_22_O_2_N requires 236.1645.

**1-Pentyl-1,2,3,4-tetrahydroisoquinoline-6,7-diol hydrochloride (4a.HCl)**

Hexanal (264 mg, 2.64 mmol) was dissolved in a mixture of acetonitrile (7.5 ml) and phosphate buffer (0.3 M, pH 6, 7.5 ml). Ascorbic acid (232 mg, 1.32 mmol) was added, followed by dopamine hydrochloride (750 mg, 3.95 mmol). The reaction vessel was flushed with argon and stirred at 60 ^o^C for 18 h. The pH of the reaction mixture was increased to 7.5 by the addition of NaOH_(aq)_ (1 M) and an extraction was performed with EtOAc (4 x 30 ml). The organic layer was dried over anhydrous Na_2_SO_4_ and the solvent was removed under reduced pressure. The residue was dissolved HCl_(aq)_ (1 M, 20 ml) and dimethyl carbonate (20 ml). The aqueous layer was washed with dimethyl carbonate (1 x 10 ml) and then co-evaporated with MeOH. The glassy solid obtained was dissolved in a 1:10 solution of water and acetonitrile before lyophilisation to afford the product as a mixture of two isomers (523 mg, 73%). To prepare the product for biological characterisation a portion of the product further was purified by preparative HPLC (Method 4, Gradient B, run time: 15 min, flow rate: 15 ml/min, Rt = 11.52 min). The purified product was dissolved in HCl_(aq)_ (0.01 M) and lyophilised to obtain the hydrochloride salt of the product as a white solid (13.4 mg). IR (neat, ν_max_/cm^-1^): 3125 br., 2928, 1670, 1523; ^1^H NMR (700 MHz, CD_3_OD) δ 6.65 (1H, s, **8‑H**), 6.61 (1H, s, **5‑H**), 4.34 (1H, dd, *J* = 7.8, 5.1 Hz, **1-H**), 3.52–3.49 (1H, dt, *J* = 12.4, 5.9, **3‑*H*H**), 3.33–3.29 (1H, m, **3‑H*H***), 3.02–2.97 (1H, m, **4-*H*H**), 2.92–2.88 (1H, m, **4‑H*H***), 2.07–1.98 (1H, m, **CHC*H*H**), 1.94–1.83 (1H, m, **CHCH*H***), 1.55–1.47 (2H, m, **CHCH_2_C*H*_2_**), 1.44–1.36 (4H, m, **2 x CH_2_**), 0.94 (3H, t, *J* = 7.0 Hz, **CH_3_**); ^13^C NMR (176 MHz, CD_3_OD) δ 146.5, 145.7, 124.1, 123.5, 116.0, 113.7, 56.5, 40.9, 34.9, 32.6, 25.9, 25.5, 23.3, 14.1; *m/z* [ES+] 236 ([M + H]^+^, 100%); *m/z* [HRMS, ES+] found [M + H]^+^ 236.1648. C_14_H_22_NO_2_ requires 236.1651.

**1-Hexyl-1,2,3,4-tetrahydroisoquinoline-6,7-diol** (**5a**)^13^ **and 1-Hexyl-1,2,3,4- tetrahydroisoquinoline-7,8-diol** (**5b**)^13^

Heptanal (177 μl, 1.27 mmol) was added to dopamine hydrochloride (200 mg, 1.05 mmol) in potassium phosphate buffer (10 ml, 0.1 M, pH 6) and acetonitrile (10 ml) under argon. The solution was stirred at 50 ^o^C for 18 h. The reaction was concentrated under vacuum, CH_2_Cl_2_/CH_3_OH (1:1, 20 ml) added, and the resulting suspension filtered to remove the solid. The filtrate was evaporated to dryness under vacuum to afford the crude products which were purified by preparative HPLC (Method 2, Gradient F) to give **5a** as a pale brown oil (Rt = 18.2 min, 225 mg, 86%) and **5b** as a pale yellow oil (Rt = 28.8 min, 25.1 mg, 10%).

*Major regioisomer* **5a**: ^1^H NMR (600 MHz; CD_3_OD) δ 6.65 (1H, s, **8-H**), 6.61 (1H, s, **5-H**), 4.36-4.32 (1H, m, **1-H**), 3.54-3.28 (2H, m, **3-H_2_**), 3.02-2.86 (2H, m, **4-H_2_**), 2.07-1.83 (2H, m, **CHC*H*_2_**), 1.55-1.33 (8H, m, **4 x CH_2_**), 0.93 (3H, t, *J* = 7.0 Hz, **CH_3_**); ^13^C NMR (150 MHz; CD_3_OD) δ 146.7, 145.9, 124.2, 123.6, 116.2, 113.8, 56.7, 41.0, 35.2, 32.8, 30.3, 26.4, 25.7, 23.6, 14.3; *m/z* [ES+] 250 ([M + H]^+^, 100%); *m/z* [HRMS, ES+] found [M + H]^+^ 250.1809, C_15_H_24_NO_2_ requires 250.1807.

*Minor regioisomer* **5b**: ^1^H NMR (600 MHz; CD_3_OD) δ 6.74 (1H, d, *J* = 8.0 Hz, **6-H**), 6.55 (1H, d, *J* = 8.0 Hz, **5-H**), 4.61 (1H, dd, *J* = 9.5 and 3.5 Hz, **1-H**), 3.51-3.28 (2H, m, **3-H_2_**), 3.03-2.92 (2H, m, **4-H_2_**), 2.17-1.79 (2H, m, **CHC*H*_2_**), 1.61-1.27 (8H, m, **4 x CH_2_**), 0.91 (3H, t, *J* = 7.0 Hz, **CH_3_**); ^13^C NMR (150 MHz; CD_3_OD) δ 144.7, 143.3, 123.2, 121.3, 120.4, 116.0, 53.4, 38.7, 33.0, 32.7 30.0, 27.0, 25.5, 23.7, 14.4; *m/z* (ES+) 250 ([M + H]^+^, 100%), 233 ([MH - OH]^+^, 90%); *m/z* [HRMS, ES+] found [M + H]^+^ 250.1810, C_15_H_24_NO_2_ requires 250.1807.

**1-Heptyl-1,2,3,4-tetrahydroisoquinoline-6,7-diol** (**6a**) **and 1-Heptyl-1,2,3,4- tetrahydroisoquinoline-7,8-diol** (**6b**)

Octanal (198 μl, 1.27 mmol) was added to dopamine hydrochloride (200 mg, 1.05 mmol) in potassium phosphate buffer (10 ml, 0.1 M, pH 6) and acetonitrile (10 ml) under argon. The solution was stirred at 50 ^o^C for 18 h. The reaction was concentrated under vacuum, CH_2_Cl_2_/CH_3_OH (1:1, 20 ml) added, and the resulting suspension filtered to remove the solid. The filtrate was evaporated to dryness under vacuum to afford the crude products which were purified by preparative HPLC (Method 2, Gradient D) to give **6a** as a yellow oil (Rt = 22.8 min, 238 mg, 85%) and **6b** as a yellow oil (Rt = 33.3 min, 30.9 mg, 11%).

*Major regioisomer* **6a**: IR (neat) ν_max_/cm^-1^: 2929, 2875, 1664, 1625, 1519; ^1^H NMR (600 MHz; CD_3_OD) δ 6.65 (1H, s, **8-H**), 6.61 (1H, s, **5-H**), 4.36-4.32 (1H, m, **1-H**), 3.53-3.28 (2H, m, **3-H_2_**), 3.02-2.86 (2H, m, **4-H_2_**), 2.97-1.83 (2H, m, **CHC*H*_2_**), 1.55-1.28 (10H, m, **5 x CH_2_**), 0.91 (3H, t, *J* = 7.0 Hz, **CH_3_**); ^13^C NMR (150 MHz; CD_3_OD) δ 146.7, 145.9, 124.3, 123.6, 116.2, 113.8, 56.7, 41.0, 35.2, 32.9, 30.5, 30.2, 26.4, 25.7, 23.7, 14.4; *m/z* [CI] 264 ([M + H]^+^, 100%), 164 ([MH - C_7_H_16_]^+^, 60%); *m/z* [HRMS, ES+]^+^ found [M + H]^+^ 264.1967, C_16_H_26_NO_2_ requires 264.1964.

*Minor regioisomer* **6b**: IR (neat) ν_max_/cm^-1^: 3031 br, 2969, 2928, 2852, 1662, 1578, 1500; ^1^H NMR (600 MHz; CD_3_OD) δ 6.74 (1H, d, *J* = 8.0 Hz, **6-H**), 6.55 (1H, d, *J* = 8.0 Hz, **5-H**), 4.61 (1H, dd, *J* = 9.5 and 3.5 Hz, **1-H**), 3.51-3.28 (2H, m, **3-H_2_**), 3.04-2.92 (2H, m, **4-H_2_**), 2.17-1.79 (2H, m, **CHC*H*_2_**), 1.61-1.27 (10H, m, **5 x CH_2_**), 0.91 (3H, t, *J* = 7.0 Hz, **CH_3_**); ^13^C NMR (150 MHz; CD_3_OD) δ 144.7, 143.3, 123.2, 121.3, 120.4, 116.0, 53.4, 38.7, 33.0, 32.9, 30.3, 30.2, 27.0, 25.5, 23.7, 14.4; *m/z* [CI] 264 ([M + H]^+^, 100%), 164 ([M + H]^+^ - C_7_H_16_, 50%); *m/z* [HRMS, ES+] found [M + H]^+^ 264.1962, C_16_H_26_NO_2_ requires 264.1964.

**1-Octyl-1,2,3,4-tetrahydroisoquinoline-6,7-diol** (**7a**) **and 1-Octyl-1,2,3,4- tetrahydroisoquinoline-7,8-diol** (**7b**)

Nonanal (218 μl, 1.27 mmol) was added to dopamine hydrochloride (200 mg, 1.05 mmol) in potassium phosphate buffer (10 ml, 0.1 M, pH 6) and acetonitrile (10 ml) under argon. The solution was stirred at 50 ^o^C for 18 h. The reaction was concentrated under vacuum, CH_2_Cl_2_/CH_3_OH (1:1, 20 ml) added, and the resulting suspension filtered to remove the solid. The filtrate was evaporated to dryness under vacuum and the mixture of products in ¾ of the crude material purified by preparative HPLC (Method 2, Gradient F, flow rate: 8 ml/min) to give **7a** as an oil (Rt = 17.3 min, 168 mg, 77%) and **7b** as a yellow oil (Rt = 23.9 min, 18.0 mg, 8%).

*Major regioisomer* **7a**: IR (neat) ν_max_/cm^-1^: 3047 br, 2927, 2859, 1669, 1617, 1530; ^1^H NMR (500 MHz; CD_3_OD) δ 6.64 (1H, s, **8-H**), 6.60 (1H, s, **5-H**), 4.35-4.29 (1H, m, **1-H**), 3.52-3.25 (2H, m, **3-H_2_**), 3.01-2.83 (2H, m, **4-H_2_**), 2.07-1.80 (2H, m, **CHC*H*_2_**), 1.55-1.22 (12H, m, **6 x CH_2_**), 0.89 (3H, t, *J* = 7.0 Hz, **CH_3_**); ^13^C NMR (125 MHz; CD_3_OD) δ 146.7, 145.9, 124.3, 123.7, 116.2, 113.9, 56.7, 41.0, 35.1, 33.0, 30.5, 30.4, 30.3, 26.4, 25.7, 23.7, 14.4; *m/z* (ES+]278 ([M + H]^+^, 100%), 261 ([MH - OH]^+^, 60%); *m/z* [HRMS, ES+] found [M + H]^+^ 278.2124, C_17_H_28_NO_2_ requires 278.2120.

*Minor regioisomer* **7b**: IR (neat) ν_max_/cm^-1^: 3320, 3047, 2928, 2852, 1664, 1625, 1506; ^1^H NMR (500 MHz; CD_3_OD) δ 6.73 (1H, d, *J* = 8.0 Hz, **6-H**), 6.54 (1H, d, *J* = 8.0 Hz, **5-H**), 4.64-4.58 (1H, m, **1‑H**), 3.51-3.25 (2H, m, **3-H_2_**), 3.03-2.90 (2H, m, **4-H_2_**), 2.17- 1.77 (2H, m, **CHC*H*_2_**), 1.61-1.21 (12H, m, **6 x CH_2_**), 0.89 (3H, t, *J* = 7.0 Hz, **CH_3_**); ^13^C NMR (125 MHz; CD_3_OD) δ 144.7, 143.3, 123.3, 121.3, 120.5, 116.0, 53.4, 38.8, 33.1, 33.0, 30.4, 30.3, 26.9, 25.5, 23.7, 14.4; *m/z* (ES+] 278 ([M + H]^+^, 100%), 261 ([MH - OH]^+^, 40%); *m/z* [HRMS, ES+] found [M + H]^+^ found 278.2114, C_17_H_28_NO_2_ requires 278.2120.

**1-Cyclopentyl-1,2,3,4-tetrahydroisoquinoline-6,7-diol** (**8a**)^16^ **and 1- Cyclopentyl-1,2,3,4-tetrahydroisoquinoline-7,8-diol** (**8b**)

Cyclopentanecarboxaldehyde (155 mg, 1.58 mmol) was added to dopamine hydrochloride (150 mg, 0.791 mmol) in potassium phosphate buffer (7.5 ml, 0.1 M, pH 6) and acetonitrile (7.5 ml) under argon. The solution was stirred at 50 ^o^C for 18 h. The reaction was concentrated under vacuum, CH_2_Cl_2_/CH_3_OH (1:1, 15 ml) added, and the resulting suspension filtered to remove the solid. The filtrate was evaporated to dryness under vacuum to afford the crude products which were purified by preparative HPLC (Method 2, Gradient F, flow rate: 8 ml/min) to give **8a** as a white solid (Rt = 10.0 min, 59.2 mg, 32%) and **8b** as a colourless oil (Rt = 26.5 min, 6.5 mg, 4%).

*Major regioisomer* **8a**: Mp. 207-210 ^o^C; IR (neat) ν_max_/cm^-1^: 3135 br, 2969, 1675, 1612, 1531; ^1^H NMR (500 MHz; CD_3_OD) δ 6.69 (1H, s, **8-H**), 6.62 (1H, s, **5-H**), 4.22 (1H, d, *J* = 8.0 Hz, **1-H**), 3.56-3.32 (2H, m, **3‑H_2_**), 3.02-2.92 (2H, m, **4-H_2_**), 2.44-2.35 (1H, m, **1-CHC*H***), 1.92-1.60 (6H, m, **3 x CH_2_**), 1.53-1.31 (2H, m, **CH_2_**); ^13^C NMR (125 MHz; CD_3_OD) δ 146.9, 145.5, 124.2, 123.7, 116.3, 114.9, 60.5, 44.6, 39.9, 31.4, 29.9, 26.0, 25.7, 25.3; *m/z* (EI) 233 ([M]^+^, 30%), 165 ([M - C_5_H_8_]^+^, 100%); *m/z* [HRMS, ES+] found [M + H]^+^ 233.1419, C_14_H_19_O_2_N requires 233.1416.

*Minor regioisomer* **8b**: IR (neat) ν_max_/cm^-1^) 3063 br, 2969, 2924, 2875, 1668, 1633, 1516; ^1^H NMR (600 MHz; CD_3_OD) δ 6.75 (1H, d, *J* = 8.0 Hz, **6-H**), 6.57 (1H, d, *J* = 8.0 Hz, **5-H**), 4.70 (1H, d, *J* = 8.5 Hz, **1-H**), 3.64-3.24 (2H, m, **3-H_2_**), 3.09-2.94 (2H, m, **4-H_2_**), 2.51-2.41 (1H, m, **1-CHC*H***), 1.96-1.42 (8H, m, **4 x CH_2_**); ^13^C NMR (150 MHz; CD_3_OD) δ 144.7, 143.8, 123.8, 121.3, 120.4, 116.1, 55.8, 44.1, 38.6, 30.5, 30.4, 25.9, 25.6, 24.7; *m/z* [CI] 234 ([M + H]^+^, 20%), 164 ([MH - C_5_H_10_]^+^, 100%); *m/z* [HRMS, ES+] found [M + H]^+^ 234.1491, C_14_H_20_O_2_N required 234.1494.

**1-(3-Methylbenzyl)-1,2,3,4-tetrahydroisoquinoline-6,7-diol** (**9a**)^11^ **and 1-(3- Methylbenzyl)-1,2,3,4-tetrahydroisoquinoline-7,8-diol** (**9b**)

To dopamine hydrochloride (150 mg, 0.791 mmol) in potassium phosphate buffer (7.5 ml, 0.1 M, pH 6) and acetonitrile (7.5 ml) under argon was added 2-(*m*-tolyl)acetaldehyde (127 mg, 0.949 mmol). The solution was stirred at 50 ^o^C for 18 h. The reaction was concentrated under vacuum, CH_2_Cl_2_/CH_3_OH (1:1, 15 ml) was added, and the resulting suspension filtered to remove the solid. The filtrate was evaporated to dryness under vacuum to afford the crude products which were separated and purified by preparative HPLC (Method 2, Gradient F) to give **9a** as a yellow oil (Rt = 13.0 min, 148 mg, 69%) and **9b** as a yellow oil (Rt = 29.4 min, 6.1 mg, 3%).

*Major regioisomer* **9a**: Mp. 60-64 ^o^C; IR (neat) ν_max_/cm^-1^: 3034 br, 2859, 1667, 1617, 1530; ^1^H NMR (500 MHz; CD_3_OD) δ 7.27 (1H, t, *J* = 7.5 Hz, **5’-H**), 7.17-7.07 (3H, m, **2’-H, 4’-H,** **6’-H**), 6.63-6.59 (2H, m, **5-H, 8-H**), 4.66-4.40 (1H, m, **1-H**), 3.50-3.20 (3H, m, **3-H_2_**, **CHC*H*H**), 3.06-2.85 (3H, m, **4-H_2_, CHCH*H***), 2.35 (3H, s, **CH_3_**); ^13^C NMR (125 MHz; CD_3_OD) δ 146.9, 145.8, 140.1, 136.5, 131.2, 130.1, 129.5, 127.6, 123.7, 116.2, 114.2, 57.8, 41.2, 41.0, 25.7, 21.4; *m/z* [CI] 270 ([M + H]^+^, 15%), 164 ([MH - C_8_H_10_]^+^, 100%); *m/z* [HRMS, ES+] found [M + H]^+^ 270.1485, C_17_H_20_O_2_N requires 270.1494.

*Minor regioisomer* **9b**: IR (neat) ν_max_/cm^-1^: 3063 br., 2945, 2924, 2844, 1672, 1617, 1502; ^1^H MNR (600 MHz; CD_3_OD) δ 7.28 (1H, t, *J* = 7.5 Hz, **5’-H**), 7.21 (1H, s, **2’-H**), 7.20-7.13 (2H, m, **4’-H, 6’-H**), 6.79 (1H, d, *J* = 8.5 Hz, **6-H**), 6.59 (1H, d, *J* = 8.5 Hz, **5-H**), 4.95.4.85 (1H, m, **1-H**), 3.67-3.60 (1H, m, **CHC*H*H**), 3.53-3.23 (2H, m, **3-H_2_**), 3.05-2.93 (3H, m, **4-H_2_,** **CHCH*H***), 2.36 (3H, s, **CH_3_**); ^13^C NMR (150 MHz; CD_3_OD) δ 144.8, 143.4, 140.1, 137.3, 131.0, 130.2, 129.4, 127.4, 123.4, 120.6, 120.4, 116.3**,** 55.0, 38.7, 38.1, 25.5, 21.5; *m/z* [CI] 270 ([M + H]^+^, 70%), 164 ([MH - C_8_H_10_]^+^, 100%); *m/z* [HRMS, ES+] found [M + H]^+^ 270.1488, C_17_H_20_O_2_N requires 270.1494.

**1-(Benzo[*d*][1,3]dioxol-5-ylmethyl)-1,2,3,4-tetrahydroisoquinoline-6,7-diol** (**10a)**^9^ **and 1-(Benzo[*d*][1,3]dioxol-5-ylmethyl)-1,2,3,4-tetrahydroisoquinoline-7,8- diol** (**10b**)

To dopamine hydrochloride (145 mg, 0.765 mmol) in potassium phosphate buffer (7.5 ml, 0.1 M, pH 6) and acetonitrile (7.5 ml) under argon was added 3,4-(methylenedioxy)phenylacetaldehyde (150 mg, 0.917 mmol). The solution was stirred at 50 ^o^C for 18 h. The reaction was concentrated under vacuum, CH_2_Cl_2_/CH_3_OH (1:1, 15 ml) was added, and the suspension filtered to remove the solid. The filtrate was evaporated to dryness under vacuum to afford the crude products which were purified by preparative HPLC (Method 2, Gradient D) to give **10a** as a yellow oil (Rt = 16.0 min, 112 mg, 49%) and **10b** as a pale yellow oil (Rt = 23.0 min, 6.2 mg, 3%).

*Major regioisomer* **10a**: Mp. 64-66 ^o^C; IR (neat) ν_max_/cm^-1^: 3018 br, 2859, 1668, 1617, 1531; ^1^H NMR (600 MHz; CD_3_OD) δ 6.85 (1H, d, *J* = 1.5 Hz, **2’-H**), 6.83 (1H, d, *J* = 8.0 Hz, **5’-H**), 6.78 (1H, dd, *J* = 8.0 and 1.5 Hz, **6’-H**), 6.64-6.61 (2H, m, **5-H**, **8-H**), 5.97 (2H, s, **OCH_2_O**), 4.62-4.58 (1H, m, **1-H**), 3.51-3.45 (1H, m, **3-*H*H**), 3.40 (1H, dd, *J* = 14.5 and 5.5 Hz, **CHC*H*H**), 3.29-3.23 (1H, m, **3-H*H***), 3.04-2.88 (3H, m, **4-H_2_**, **CHCH*H***); ^13^C NMR (150 MHz; CD_3_OD) δ 149.9, 148.8, 146.9, 145.9, 130.1, 123.9, 123.6, 116.2, 114.1, 110.5, 109.7, 102.6, 57.8, 40.9, 25.7; *m/z* (ES+]300 ([M + H]^+^, 100%), 283 ([MH - OH]^+^, 80%), 164 ([MH - C_8_H_8_O_2_]^+^, 100%); *m/z* [HRMS, ES+] found [M + H]^+^ 300.1221, C_17_H_18_NO_4_ requires 300.1236.

*Minor regioisomer* **10b**: IR (neat) ν_max_/cm^-1^: 3029 br, 1667, 1625; ^1^H NMR (600 MHz; CD_3_OD) δ 6.88- 6.83 (3H, m, **2’-H**, **5’-H**, **6’-H**), 6.79 (1H, d, *J* = 8.0 Hz, **6-H**), 6.59 (1H, d, *J* = 8.0 Hz, **5-H**), 5.96 (2H, s, **OCH_2_O**), 4.85 (1H, dd, *J* = 10.5 and 3.0 Hz, **1-H**), 3.59-3.48 (2H, m, **3-*H*H**, **CHC*H*H**), 3.30-3.24 (1H, m, **3-H*H***), 3.05-2.92 (3H, m, **4-H_2_, CHCH*H***); ^13^C NMR (150 MHz; CD_3_OD) δ 149.9, 148.7, 144.8, 143.3, 130.9, 123.6, 123.3, 120.6, 120.3, 116.3, 110.3, 109.7, 102.6, 55.1, 38.6, 37.8, 25.5; *m/z* (ES+] 300 ([M + H]^+^, 40%), 164 ([MH - C_8_H_8_O_2_]^+^, 100%); *m/z* [HRMS, ES+] found [M + H]^+^ 300.1236, C_17_H_18_NO_4_, requires 300.1236.

**1-Pentyl-1,2,3,4-tetrahydroisoquinolin-6-ol** (**11a**)^2^ **and 1-Pentyl-1,2,3,4- tetrahydroisoquinolin-8-ol** (**11b**)

Hexanal (126 μl, 1.03 mmol) was added to a solution of *m*-tyramine (150 mg, 0.688 mmol) in potassium phosphate buffer (7.5 ml, 0.1 M, pH 6) and acetonitrile (7.5 ml) under argon. The solution was stirred at 50 ^o^C for 18 h. The reaction was concentrated under vacuum, CH_2_Cl_2_/CH_3_OH (1:1, 15 ml) was added and the resulting suspension filtered to remove the solid. The filtrate was evaporated to dryness under vacuum to afford the crude products, which were purified by preparative HPLC (Method 2, Gradient A, run time: 30 min) to give **11a** as a pale yellow oil (Rt = 24.5 min, 121 mg, 81%) and **11b** as a pale yellow oil (Rt = 28.8 min, 17.3 mg, 12%).

*Major regioisomer* **11a**: IR (neat) ν_max_/cm^-1^: 3031 br, 2958, 2927, 2867, 1668, 1617, 1523; ^1^H NMR (600 MHz; CD_3_OD) δ 7.10 (1H, d, *J* = 8.5 Hz, **8-H**), 6.72 (1H, dd, *J* = 8.5 and 2.5 Hz, **7‑H**), 6.64 (1H, d, *J* = 2.5 Hz, **5-H**), 4.42-4.38 (1H, m, **1-H**), 3.55-3.31 (2H, m, **3-H_2_**), 3.10-2.95 (2H, m, **4-H_2_**), 2.10-1.84 (2H, m, **CHC*H*_2_**), 1.55-1.35 (6H, m, **3 x CH_2_**), 0.94 (3H, t, *J* = 7.0 Hz, **CH_3_**); ^13^C NMR (150 MHz; CD_3_OD) δ 156.9, 132.4, 127.3, 122.6, 114.5, 114.2, 55.2, 39.1, 33.5, 31.2, 24.8, 24.6, 21.9, 12.7; *m/z* [CI] 220 ([M + H]^+^, 80%), 148 ([MH - C_5_H_12_]^+^, 20%); *m/z* [HRMS, ES+] found [M + H]^+^ 220.1706, C_14_H_22_NO requires 220.1701.

*Minor regioisomer* **11b**: IR (neat) ν_max_/cm^-1^: 3039 br, 2961, 2930, 2867, 1667, 1633, 1592; ^1^H NMR (600 MHz; CD_3_OD) δ 7.11 (1H, t, *J* = 8.0 Hz, **6-H**), 6.72-6.68 (2H, m, **5-H**, **7-H**), 4.60 (1H, dd, *J* = 9.5 and 3.5 Hz, **1-H**), 3.55-3.32 (2H, m, **3-H_2_**), 3.12-3.00 (2H, m, **4-H_2_**), 2.14-1.79 (2H, m, **CHCH_2_**), 1.62-1.34 (6H, m, **3 x CH_2_**), 0.94 (3H, t, *J* = 7.0 Hz, **CH_3_**); ^13^C NMR (150 MHz; CD_3_OD) δ 155.3, 133.4, 129.8, 121.1, 120.8, 114.2, 53.2, 38.3, 33.0, 32.5, 26.7, 25.9, 23.5, 14.3; *m/z* [CI] 220 ([M + H]^+^, 30%), 148 ([MH - C_5_H_12_]^+^, 100%); *m/z* [HRMS, ES+] found [M + H]^+^ 220.1698, C_14_H_22_NO requires 220.1701.

**1-Pentyl-1,2,3,4-tetrahydroisoquinolin-6-ol.HCl** (**11a.HCl**)^2^

To a solution of hexanal (0.140 ml, 1.15 mmol) and sodium ascorbate (113 mg, 0.570 mmol) in acetonitrile (5 ml) and phosphate buffer (0.3 M, pH 6, 5 ml), was added *meta*-tyramine hydrochloride (300 mg, 1.73 mmol). The reaction vessel was flushed with argon and the reaction was stirred at 50 ^o^C for 18 h. The pH of the reaction mixture was adjusted to 8 with NaOH_(aq)_ (1 M), and the aqueous layer was extracted with EtOAc (3 x 20 ml). The combined organic layer was dried under reduced pressure. The crude product was dissolved in dimethyl carbonate (10 ml) and HCl_(aq)_ (1 M, 10 ml). The two layers were separated, and the aqueous layer was washed with dimethyl carbonate (4 x 5 ml)_._ The solvent was removed from the aqueous layer to yield the hydrochloride salt of the product as a solid (139 mg, 47%). To prepare the product for biological characterisation a portion of the product was further purified by preparative HPLC (Method 4,Gradient A, run time: 20 min, Rt = 13.1 min) to yield the TFA salt of the product. This solid was then dissolved in HCl_(aq)_ (0.01 M) and lyophilised to yield the hydrochloride salt of the product as a white solid (12.4 mg). IR (neat, ν_max_/cm^-1^): 3378 br., 2959, 1670, 1615, 1505; ^1^H NMR (700 MHz, CD_3_OD) δ 7.10 (1H, d, *J* = 8.5 Hz, **8-H**), 6.72 (1H, dd, *J* = 8.5, 2.5, **7-H**), 6.64 (1H, d, *J* = 2.5, **5-H**), 4.10 (1H, dd, *J* = 8.1, 5.1, **1-H**), 3.53 (1H, dt, *J* = 12.6, 6.1, **3-*H*H**), 3.37–3.32 (1H, m, **3‑H*H***), 3.10–3.05 (1H, m, **4-*H*H**), 3.01–2.97 (1H, m, **4‑H*H***), 2.09–2.03 (1H, m, **CHC*H*H**), 1.91–1.85 (1H, m, **CHCH*H***), 1.54–1.47 (2H, m, **CHCH_2_C*H*_2_**), 1.43–1.38 (4H, m, **2 x CH_2_**), 0.94 (3H, t, *J* = 7.1 Hz, **CH_3_**); ^13^C NMR (151 MHz, CD_3_OD) δ 158.4, 134.0, 128.8, 124.2, 116.1, 115.8, 56.8, 40.7, 35.0, 32.7, 26.4, 26.1, 23.5, 14.3; *m/z* [ES+] 220 ([M + H]^+^, 100%), 203 ([MH‑OH]^+^ 17%); *m/z* [HRMS, ES+] found [M + H]^+^ 220.1696. C_14_H_22_NO requires 220.1696.

**(1*S*)-1-Pentyl-1,2,3,4-tetrahydroisoquinoline-6,7-diol ((1*S*)-4a).HCl**^17^

To dopamine hydrochloride (28 mg, 0.15 mmol) and sodium ascorbate (10 mg, 50 µmol) in HEPES buffer (9 ml), was added a solution of hexanal (30 mg, 0.30 mmol) in acetonitrile (1 ml) followed by a solution of *Tf*NCSΔ33 (1 mg) in water (0.1 ml). The reaction mixture was then stirred at 37 ^o^C for 18 h. A solution of HCl_(aq)_ (2 M, 2 ml) was added, and denatured *Tf*NCSΔ33 removed by centrifugation (13,600 rpm, 20 min). The supernatant was lyophilised and then purified by preparative HPLC (Method 4, Gradient B, run time: 20 min, flow rate: 15 ml/min, Rt = 12.8 min). The solid obtained was dissolved in 0.1 M HCl_(aq)_ and lyophilised to yield the hydrochloride salt of the product as a white solid (7.3 mg, 18%), 79% ee (chiral HPLC method 1). IR (neat) ν_max_/cm^-1^: 3125 br, 2928, 1670, 1523; ^1^H NMR (700 MHz; CD_3_OD) δ 6.65 (1H, s, **Ar-H**), 6.61 (1H, s, **Ar-H**), 4.34 (1H, dd, *J* = 7.6 and 5.1 Hz, **1‑H**), 3.50 (1H, dt, *J* = 12.3 and 6.0 Hz, **3-*H*H**), 3.33-3.29 (1H, m, **3‑H*H***), 3.01-2.97 (1H, m, **4-*H*H**), 2.92-2.88 (1H, m, **4-H*H***), 2.05-2.00 (1H, m, **9-*H*H**), 1.91-1.85 (1H, m, **9‑H*H***), 1.55-1.46 (2H, m, **CH_2_**), 1.45-1.37 (4H, m, **2 x CH_2_**), 0.94 (3H, t, *J*= 7.0 Hz, **CH_3_**); ^13^C NMR (151 MHz; CD_3_OD) δ 146.7, 145.9, 124.3, 123.7, 116.2, 113.9, 56.7, 41.0, 35.1, 32.7, 26.1, 25.7, 23.5, 14.3; *m/z* [ES+] 236 ([M + H]^+^, 100%); *m/z* [HRMS, ES+] found [M + H]^+^ 236.1645. C_14_H_22_NO_2_ requires 236.1651.

**(1*S*)-1-Pentyl-1,2,3,4-tetrahydroisoquinolin-6-ol ((1*S*)-11a).HCl**

To a solution of *meta*-tyramine hydrochloride (26 mg, 0.15 mmol) and sodium ascorbate (10 mg, 0.05 mmol) in HEPES buffer (9 ml), was added hexanal (13 µl, 0.10 mmol) in acetonitrile (1 ml), followed by *Tf*NCSΔ33 (2 mg) in water (0.2 ml). The reaction mixture was then left to stir at 37 ^o^C for 18 h and then HCl_(aq)_ (2 M, 1 ml) added. The denatured *Tf*NCSΔ33 was removed by centrifugation (13,600 rpm, 20 min) and the supernatant lyophilised. The product was purified by preparative HPLC (Method 4, Gradient A, run time: 20 min, Rt = 13.2 min) to yield the TFA salt which was dissolved in HCl_(aq)_ (0.1 M) and lyophilised to yield the hydrochloride salt as an oil (4.2 mg, 11%), 78% ee (chiral HPLC method 2). IR (neat) ν_max_/cm^-1^: 3378 br, 2959, 1670, 1615, 1505; ^1^H NMR (700 MHz; D_2_O) δ 7.90 (1H, d, *J* = 8.5 Hz, **8‑H**), 6.91 (1H, dd, *J* = 8.5 and 2.6 Hz, **7‑H**), 6.85 (1H, d, *J* = 2.6 Hz, **5-H**), 4.57 (1H, dd, *J* = 8.1, 5.1 Hz, **1-H**), 3.65 (1H, dt, *J* = 12.7 and 6.2 Hz, **3-*H*H**), 3.49-3.44 (1H, m, **3‑H*H***), 3.21-3.16 (1H, m, **4-*H*H**), 3.13-3.08 (1H, m, **4‑H*H***), 2.18-2.12 (1H, m, **CHC*H*H**), 2.02-1.96 (1H, m, **CHCH*H***), 1.57-1.49 (2H, m, **CHCH_2_C*H*_2_**), 1.47-1.38 (4H, m, **2 x CH_2_**), 0.96 (3H, t, *J* = 7.1 Hz, **CH_3_**); ^13^C NMR (176 MHz; D_2_O) δ 155.5, 133.8, 128.7, 124.7, 115.5, 115.0, 55.8, 39.7, 33.7, 31.3, 25.3, 24.7, 22.2, 13.7; *m/z* [ES+] 220 ([M + H]^+^, 100%), 203 ([MH - OH]^+^ 17%); *m/z* [HRMS, ES+] found [M + H]^+^ 220.1696, C_14_H_22_NO requires 220.1696.

**1-Pentyl-1,2,3,4-tetrahydroisoquinoline-6,8-diol (12).HCl^2^**

To a solution of 5-(2-aminoethyl)benzene-1,3-diol hydrobromide (60.0 mg, 0.26 mmol) and sodium ascorbate (40 mg, 0.2 mmol) in phosphate buffer (0.3 M, pH 6, 5.0 ml) and acetonitrile (5 ml), was added hexanal (75 µl, 0.6 mmol). The reaction mixture was stirred at 50 ^o^C for 18 h, the pH adjusted to 8 with NaOH_(aq)_ (1 M) and the product extracted with EtOAc (3 x 20 ml). The combined organic layers were dried over anhydrous Na_2_SO_4_ and the solvent removed under reduced pressure. The crude product was dissolved in HCl_(aq)_ (1 M, 10 ml) and dimethyl carbonate (DMC) (10 ml) and the aqueous layer extracted with DMC (4 x 5 ml). The solvent was then removed from the aqueous layer under reduced pressure to afford the hydrochloride salt of the product as a yellow solid (39.5 mg, 57%). To prepare the product for biological characterisation a portion of the product was further purified by preparative HPLC (Method 4, Gradient B, 20 min, 15 ml/min, Rt = 12.1 min). The solid was dissolved in HCl_(aq)_ (0.01 M) and lyophilised to yield the hydrochloride salt as a yellow solid (9.2 mg). IR (neat) ν_max_/cm^‑1^: 3349, 3231, 2929 1608, 1588; ^1^H NMR (700 MHz; D_2_O) δ 6.29 (1H, d, *J* = 2.3 Hz, **Ar-H**), 6.27 (1H, d, *J* = 2.3 Hz, **Ar-H**), 4.51 (1H, dd, *J*= 9.6 and 3.8 Hz, **1-H**), 3.43 (1H, ddd, *J* = 12.8, 9.8 and 5.9 Hz, **3-*H*H**), 3.33 (1H, ddd, *J*= 12.8, 6.3 and 4.2 Hz, **3‑H*H***) 2.99-2.89 (2H, m, **4‑H_2_**), 1.93-1.88 (1H, m, **CHC*H*H**), 1.75-1.70 (1H, m, **CHCH*H***), 1.45-1.35 (2H, m, **CH_2_**), 1.30‑1.23 (4H, m, **2 x CH_2_**), 0.93 (3H, t, *J* = 7.1 Hz, **CH_3_**); ^13^C NMR (176 MHz; D_2_O) δ 156.3, 154.5, 134.4, 113.2, 107.4, 101.8, 51.9, 37.3, 32.1, 31.1, 25.3, 25.0, 22.2, 13.8; *m/z* [ES+] 237 ([M + H]^+^, 100%); *m/z* [HRMS, ES+] found [M + H]^+^ 236.1655. C_14_H_22_NO_2_ requires 236.1651.

**5-Bromo-1-pentyl-1,2,3,4-tetrahydroisoquinolin-8-ol** (**13**)

Hexanal (49.0 μl, 0.404 mmol) was added to a solution of 2-(2-bromo-5-hydroxyphenyl)ethan-1-amine hydrobromide (100 mg, 0.337 mmol) in potassium phosphate buffer (5 ml, 0.5 M, pH 6) and acetonitrile (5 ml) under argon. The solution was stirred at 50 ^o^C for 18 h. Further hexanal (21.0 μl, 0.169 mmol) was added to the reaction mixture which was stirred at 50 ^o^C for 5 h. The reaction was concentrated under vacuum, CH_2_Cl_2_/CH_3_OH (1:1, 15 ml) added and the suspension filtered to remove the solid. The filtrate was evaporated under vacuum to give the crude product, which was purified by preparative HPLC (Method 1, Rt = 6.5 min). The appropriate fractions were combined and co-evaporated with methanol (x 3) to give the product as a pale yellow oil (14.2 mg, 14%). IR (neat) ν_max_/cm^-1^: 3031 br, 2953, 2928, 2859, 1666, 1641, 1581; ^1^H NMR (600 MHz; CD_3_OD) δ 7.39 (1H, d, *J* = 8.5 Hz, **6-H**), 6.70 (1H, d, *J* = 8.5 Hz, **7-H**), 4.62 (1H, dd, *J* = 9.5 and 3.5 Hz, **1-H**), 3.57-3.42 (2H, m, **3-H_2_**), 3.09-2.95 (2H, m, **4-H_2_**), 2.10-1.79 (2H, m, **CHC*H*_2_**), 1.64-1.47 (2H, m, **CHCH_2_C*H*_2_**), 1.45-1.34 (4H, m, **CH_2_C*H*_2_CH_3_**), 0.94 (3H, t, *J* = 7.0 Hz, **CH_3_**); ^13^C NMR (150 MHz; CD_3_OD) δ 154.8, 133.4, 132.6, 123.8, 115.9, 114.5, 52.7, 37.6, 32.6, 32.4, 27.4, 26.7, 23.4, 14.3; *m/z* [CI] 300 ([^81^M + H]^+^, 20%), 298 ([^79^M + H]^+^, 25%), 219 ([MH - Br]^+^, 35%), 131 ([MH - C_5_H_12_BrO]^+^, 35%); *m/z* [HRMS, ES+] found [M + H]^+^ 298.0807, C_14_H_21_NO^79^Br requires 298.0811.

**5-Bromo-1-pentyl-1,2,3,4-tetrahydroisoquinoline-7,8-diol** (**14**)

Hexanal (117 μl, 0.958 mmol) was added to a solution of 2-(2-bromo-4,5-dihydroxyphenyl)ethan-1-amine hydrobromide (200 mg, 0.639 mmol) in potassium phosphate buffer (7.5 ml, 0.1 M, pH 6) and acetonitrile (7.5 ml) under argon. The solution was stirred at 50 ^o^C for 18 h. The reaction was concentrated under vacuum, CH_2_Cl_2_/CH_3_OH (1:1, 15 ml) added and the suspension filtered to remove the solid. The filtrate was evaporated under vacuum to give the crude product which was purified by preparative HPLC (Method 2, Gradient C, Rt = 30.0 min) to give the product as a yellow oil (13.2 mg, 7%). IR (neat) ν_max_/cm^-1^: 3039 br, 2953, 2930, 2859, 1667, 1625; ^1^H NMR (600 MHz; CD_3_OD) δ 7.02 (1H, s, **Ar‑H**), 4.63 (1H, dd, *J* = 9.5 and 3.5 Hz, **1-H**), 3.53-3.38 (2H, m, **3-H_2_**), 3.00-2.87 (2H, m, **4‑H_2_**), 2.12-1.79 (2H, m, **CHC*H*_2_**), 1.63-1.35 (6H, m, **3 x** **CH_2_**), 0.94 (3H, t, *J* = 7.0 Hz, **CH_3_**); ^13^C NMR (150 MHz; CD_3_OD) δ 144.2, 141.6, 121.7, 120.7, 117.7, 112.0, 51.3, 36.3, 31.0, 30.9, 25.1, 21.9, 12.8; *m/z* (ES+) 316 ([^81^M + H]^+^, 90%), 314 ([^79^M + H]^+^, 100%); *m/z* [HRMS, ES+] found [M + H]^+^ 314.0708, C_14_H_21_NO_2_^79^Br requires 314.0756.

**5-Bromo-1-pentyl-1,2,3,4-tetrahydroisoquinoline-6,8-diol** (**15**)

Hexanal (53.0 μl, 0.433 mmol) was added to a solution of 2-(2-bromo-4,5-dihydroxyphenyl)ethan-1-amine trifluoroacetic acid salt (100 mg, 0.289 mmol) in potassium phosphate buffer (3.4 ml, 0.1 M, pH 6) and acetonitrile (3.4 ml) under argon. The solution was stirred at 50 ^o^C for 18 h, concentrated under vacuum, CH_2_Cl_2_/CH_3_OH (1:1, 7 ml) added to the resulting residue and the suspension filtered. The filtrate was evaporated to dryness under vacuum and the crude product purified by preparative HPLC (Method 2, Gradient E, run time: 20 min, flow rate: 8 ml/min, Rt = 7.8 min) to give the product as a yellow oil (65.0 mg, 72%). IR (neat) ν_max_/cm^-1^: 3039 br, 2959, 2938, 2867, 1666, 1588; ^1^H NMR (600 MHz; CD_3_OD) δ 6.45 (1H, s, **7-H**), 4.53 (1H, dd, *J* = 9.5 and 3.5 Hz, **1-H**), 3.54-3.39 (2H, m, **3-H_2_**), 3.08-2.93 (2H, m, **4-H_2_**), 2.08-1.76 (2H, m, **CHC*H*_2_**), 1.61-1.44 (2H, m, **CHCH_2_C*H*_2_**), 1.43-1.34 (4H, m, **CH_2_C*H*_2_CH_3_**), 0.94 (3H, t, *J* = 7.0 Hz, **CH_3_**); ^13^C NMR (150 MHz; CD_3_OD) δ 155.7, 155.4, 133.1, 114.6, 102.6, 102.5, 52.6, 37.7, 33.0, 32.5, 27.6, 26.6, 23.5, 14.3; *m/z* [ES+] 316 ([^81^M + H]^+^, 100%), 314 ([^79^M + H]^+^, 100%), 229 ([^81^MH - C_5_H_11_O]^+^, 65%), 227 ([^79^M + H]^+^ - C_5_H_11_O, 65%); *m/z* [HRMS, ES+] found [M + H]^+^ 314.0801, C_14_H_21_NO_2_^79^Br required 314.0756.

**1-(Benzo[*d*][1,3]dioxol-4-yl)-5-bromo-1,2,3,4-tetrahydroisoquinolin-8-ol** (**36**)^9^

A solution of 2,3-(methylenedioxy)benzaldehyde (36.4 mg, 0.242 mmol) in acetonitrile (3 ml) was added to 2-(2-bromo-5-hydroxyphenyl)ethan-1-amine hydrobromide (60.0 mg, 0.202 mmol) in potassium phosphate buffer (3 ml, 0.5 M, pH 6) under argon and stirred at 80 ^o^C for 21 h. The reaction mixture was concentrated *in vacuo*, CH_2_Cl_2_/CH_3_OH (1:1, 10 ml) added to the resulting residue and the suspension filtered. The filtrate was evaporated to dryness under vacuum and the crude product purified by preparative HPLC (Method 1, Rt = 4.4 min). Fractions were concentrated and co-evaporated with methanol (x 3) to give the product as an oil (8.7 mg, 12%). ^1^H NMR (600 MHz; CD_3_OD) δ 7.48 (1H, d, *J* = 8.5 Hz, **6-H**), 6.89 (1H, d, *J* = 8.0 Hz, **4’-H**), 6.82 (1H, t, *J* = 8.0 Hz, **5’-H**), 6.68 (1H, d, *J* = 8.5 Hz, **7-H**), 6.43 (1H, d, *J* = 8.0 Hz, **6’-H**), 6.08 (1H, d, *J* = 1.0 Hz, **OC*H*HO**), 6.03 (1H, d, *J* = 1.0 Hz, **OCH*H*O**), 5.90 (1H, s, **1-CH**), 3.51-3.28 (2H, m, **3-H_2_**), 3.21-3.01 (2H, m, **4-H_2_**); ^13^C NMR (150 MHz; CD_3_OD) δ 155.2, 149.4, 147.6, 134.2, 133.7, 123.4, 123.1, 120.3, 118.1, 115.9, 114.2, 110.7, 103.2, 50.5, 38.0, 27.5; *m/z* [ES+] 350 ([^81^M + H]^+^, 95%), 348 ([^79^M + H]^+^, 100%); *m/z* [HRMS ES+] found 348.0235, C_16_H_15_NO_3_^79^Br requires 348.0235.

***N-*(4-Methoxyphenethyl)hexanamide** **(17)**

Triethylamine (4.15 ml, 29.8 mmol) was added to a stirred solution of 2-(4-methoxyphenyl)ethan-1-amine (900 mg, 5.95 mmol) in CH_2_Cl_2_ (65 ml) at 0 ^o^C. After 5 min, a solution of hexanoyl chloride (1 ml, 7.14 mmol) in CH_2_Cl_2_ (15 ml) was added dropwise at 0 ^o^C. After stirring the reaction at RT for 24 h, water (100 ml) was added. The aqueous phase was extracted with CH_2_Cl_2_ (3 x 50 ml), the combined organic extracts washed with brine, dried (Na_2_SO_4_) and solvent removed *in vacuo* to give the crude product which was purified by column chromatography (EtOAc/petroleum ether 40-60, 1:1) to give the product as a white solid (1.34 g, 90%). R*f* = 0.34 (EtOAc/petroleum ether 40-60, 1:1); Mp. 78-79 ^o^C; IR (CDCl_3_ cast) ν_max_/cm^-1^: 3308, 2961, 2929, 2891, 1637, 1617, 1544, 1514; ^1^H NMR (500 MHz; CDCl_3_) δ 7.10 (2H, d, *J* = 8.5 Hz, **2 x 2-H**), 6.84 (2H, d, *J* = 8.5 Hz, **2 x 3-H**), 5.41 (1H, br s, **NH**), 3.79 (3H, s, **OCH_3_**), 3.52-3.45 (2H, m, **CH_2_N**), 2.75 (2H, t, *J* = 7.0 Hz, **C*H*_2_CH_2_N**), 2.11 (2H, t, *J* = 7.5 Hz, **COCH_2_**), 1.62-1.20 (6H, m, **3 x CH_2_**), 0.88 (3H, t, *J* = 7.0 Hz, **CH_3_**); ^13^C NMR (125 MHz; CDCl_3_) δ 173.2, 158.4, 131.0, 129.8, 114.1, 55.3, 40.7, 36.9, 34.9, 31.5, 25.5, 22.5, 14.0; *m*/*z* [EI] 249 (M^+^, 5%), 134 ([M - C_7_H_15_O]^+^, 100%); *m*/*z* [HRMS, ES+] found [M]^+^ 249.1728, C_15_H_23_NO_2_ requires 249.1729.

**7-Methoxy-1-pentyl-1,2,3,4-tetrahydroisoquinoline (18)** **and 6-Methoxy-1- pentyl-1,2,3,4-tetrahydroisoquinoline** **(19)**

To a solution of *N-*(4-methoxyphenethyl)hexanamide (100 mg, 0.401 mmol) in xylene (5 ml), was added P_2_O_5_ (142 mg, 1.00 mmol) and POCl_3_ (0.336 ml, 3.61 mmol), and the mixture was heated at reflux for 20 h. After cooling to RT, the reaction mixture was quenched with water (10 ml), then 2 M NaOH was added until the solution was basic. The aqueous phase was extracted with EtOAc (3 x 20 ml). The organic extracts were combined, washed with brine, dried (Na_2_SO_4_) and solvent removed *in vacuo* to yield the intermediate imine which was used directly in the next step. To a solution of the imine in methanol (10 ml) at 0 ^o^C, was added sodium borohydride (121 mg, 3.21 mmol) portion wise and the mixture was stirred at RT for 2 h. The reaction was concentrated under vacuum, water (15 ml) was added, and the product extracted with EtOAc (3 x 25 ml). The organic extracts were combined, washed with brine, dried (Na_2_SO_4_) and solvent evaporated *in vacuo* to give the crude product as a mixture of products **18** and **19** (56.7 mg, 37%) as an oil, in a ratio 1.6:1 as determined by ^1^H NMR spectroscopy. ^1^H NMR (600 MHz; CD_3_OD) δ 7.20 (1H, d, *J* = 8.5 Hz, **minor Ar‑H**), 7.16 (1H, d, *J* = 8.5 Hz, **major Ar-H**), 6.90-6.85 (2H, m, **major Ar-H, minor Ar-H**), 6.81 (1H, d, *J* = 2.5 Hz, **major Ar-H**), 6.79 (1H, d, *J* = 2.5 Hz, **minor Ar-H**), 4.51-4.47 (1H, m, **major 1-H**), 4.46-4.43 (1H, m, **minor 1-H**), 3.79-3.78 (6H, m, **major OCH_3_, minor OCH_3_**), 3.59-3.32 (4H, m, **major 3-H_2_, minor 3-H_2_**), 3.16-2.97 (4H, m, **major 4-H_2_, minor 4-H_2_**), 2.14-1.87 (4H, m, **major CH_2_, minor CH_2_**), 1.57-1.34 (12H, m, **major 3 x CH_2_, minor 3 x CH_2_**), 0.98-0.91 (6H, m, **major CH_3_, minor CH_3_**); *m*/*z* [ES+] 234 ([M + H]+, 100%).

**1-Pentyl-1,2,3,4-tetrahydroisoquinolin-7-ol** (**16**)

To a stirred solution of THIQ regioisomers **18** and **19** (34.0 mg, 0.146 mmol) in CH_2_Cl_2_ (0.7 ml) was added a solution of 1 M boron tribromide in CH_2_Cl_2_ (0.364 ml, 0.364 mmol) at ‑78 ^o^C. The reaction was warmed to RT and stirred for 24 h. The reaction was then cooled to 0 ^o^C and quenched by the addition of methanol (2 ml). The solution was stirred at RT for 3 h and concentrated *in vacuo*. Further methanol (2 ml) was added, and solvent evaporated. This was repeated until no white fumes were observed upon addition of methanol, to afford a mixture of products which were purified by preparative HPLC (Method 2, Gradient A) to give **16** as a pale yellow oil (Rt = 32.6 min, 13.8 mg, 43%) and **11a** as a colourless oil (Rt = 37.1 min, 7.3 mg, 23%).

*Major regioisomer* **16**: IR (neat) ν_max_/cm^-1^: 3039 br, 2959, 2938, 2867, 1668, 1625, 1506; ^1^H NMR (600 MHz; CD_3_OD) δ 7.06 (1H, d, *J* = 8.0 Hz, **5-H**), 6.74 (1H, dd, *J* = 8.0 and 2.5 Hz, **6-H**), 6.68 (1H, d, *J* = 2.5 Hz, **8-H**), 4.45-4.41 (1H, m, **1-H**), 3.57-3.29 (2H, m, **3-H_2_**), 3.07-2.93 (2H, m, **4-H_2_**), 2.11-1.88 (2H, m, **CHC*H*_2_**), 1.57-1.36 (6H, m, **3 x CH_2_**), 0.95 (3H, t, *J* = 7.0 Hz, **CH_3_**); ^13^C NMR (150 MHz; CD_3_OD) δ 157.7, 134.4, 131.4, 123.0, 116.7, 113.6, 56.9, 41.0, 35.0, 32.7, 26.2, 25.5, 23.5, 14.3; *m/z* [CI] 220 ([M + H]^+^, 100%), 148 ([MH - C_5_H_12_]^+^, 50%); *m/z* [HRMS, ES+] found [M + H]^+^ 220.1699, C_14_H_22_NO requires 220.1701.

*Minor regioisomer* **11a** – characterisation data provided above.

***N*-(3-Methoxyphenethyl)hexanamide (22)**

#####

To a solution of hexanoic acid (0.625 ml, 5.00 mmol) and 2-(3-methoxyphenyl)ethan-1-amine (830 mg, 5.50 mmol) in TAME (10 ml), with a Dean-Stark adapter attached, was added B(OCH_2_CF_3_)_3_ (0.1 ml, 0.5 mmol). The reaction mixture was heated at reflux for 18 h and the solvent removed *in vacuo*. Water (20 ml) was added to the crude product and the pH decreased to 3 with HCl_(aq)_ (1 M). The aqueous layer was extracted with EtOAc (3 x 20 ml), and the combined organic layers washed with saturated NaHCO_3(aq)_ (3 x 20 ml). The solvent was removed *in vacuo* to yield the product as a yellow oil (1.18 g, 86%). IR (neat) ν_max_/cm^-1^: 3278, 2928, 1641, 1601, 1584, 1546; ^1^H NMR (600 MHz; CDCl_3_) δ 7.22 (1H, t, *J* = 8.1 Hz, **5-H**), 6.78 (2H, dd, *J* = 8.1 and 2.3 Hz, **4-H** & **6‑H**), 6.74 (1H, t, *J* = 2.3 Hz, **2‑H**), 5.49 (1H, br s, **NH**), 3.80 (3H, s, **OCH_3_**), 3.52 (2H, td, *J* = 6.9 and 5.9 Hz, **C*H*_2_NH**), 2.79 (2H, t, *J* = 6.9 Hz, **C*H*_2_CH_2_NH**), 2.11 (2H, t, *J*= 7.4 Hz, **NHCOC*H*_2_**), 1.59 (2H, pentet, *J* = 7.4 Hz, **COCH_2_C*H*_2_**), 1.35-1.22 (4H, m, **2 x CH_2_**), 0.88 (3H, t, *J* = 7.1 Hz, **CH_3_**); ^13^C NMR (151 MHz; CDCl_3_) δ 173.3, 160.0, 140.7, 129.7, 121.7, 114.6, 112.0, 55.3, 40.5, 36.9, 35.9, 31.6, 25.6, 22.5, 14.0; *m/z* [ES+] 270 ([M + Na]^+^, 20%), 250 ([M + H]^+^, 100%), 152 ([MH - C_6_H_11_O]^+^, 55%); *m/z* [HRMS, ES+] found [M + H]^+^ 250.1807. C_15_H_24_NO_2_ requires 250.1807.

##### ***N*-(3,4-Dimethoxyphenethyl)hexanamide (23)**^16^

To a solution of 3,4-methoxyphenylamine (0. 93 ml, 5.50 mmol) in TAME, with a Dean-Stark adapter attached, was added hexanoic acid (0.625 ml, 5.00 mmol) and B(OCH_2_CF_3_)_3_ (0.1 ml, 0.5 mmol). The reaction was heated at reflux for 18 h and the solvent removed *in vacuo*. The crude product was dissolved in water (10 ml) and MeOH (10 ml) and Amberlite^®^ IRA‑743 (0.5 g) was added. The suspension was stirred for 1 h, filtered and washed with EtOAc, and the solvent removed under reduced pressure. The crude product was added to water (20 ml) and the pH adjusted to 3 with HCl_(aq)_ (1 M). The aqueous layer was then extracted with EtOAc (3 x 20 ml) and the combined organic extracts washed with saturated NaHCO_3(aq)_ (3 x 20 ml), dried (Na_2_SO_4_) and the solvent removed *in vacuo* to yield the product as a white solid (1.35 g, 97%). Mp. 61-63 ^o^C (lit. 59-62 ^o^C);^16^ IR (neat) ν_max_/cm^-1^: 3313, 2949, 1635; ^1^H NMR (600 MHz; CDCl_3_) δ 6.80 (1H, d, *J* = 7.8 Hz, **5-H**), 6.73-6.71 (2H, m, **2‑H** & **6-H**), 5.48 (1H, br s, **NH**), 3.86 (3H, s, **OCH_3_**), 3.85 (3H, s, **OCH_3_**), 3.49 (2H, td, *J* = 7.0 & 5.8 Hz, **CH_2_NH**), 2.75 (2H, t, *J* = 6.9 Hz, **C*H*_2_CH_2_NH**), 2.11 (2H, t, *J* = 7.6 Hz, **NHCOC*H*_2_**), 1.59 (2H, pentet, *J* = 7.6 Hz, **COCH_2_C*H*_2_**), 1.27 (4H, m, **2 x CH_2_**), 0.88 (3H, t, *J* = 7.1 Hz, **CH_3_**); ^13^C NMR (151 MHz; CDCl_3_) δ 173.2, 149.2, 147.8, 131.6, 120.8, 112.0, 111.4, 56.0, 56.0, 40.7, 36.9, 35.4, 31.6, 25.6, 22.5 14.0; *m/z* [ES+] 302 ([M + Na]^+^, 30%), 280 ([M + H]^+^, 100%), 182 ([MH - C_6_H_11_O]^+^, 32%).

***N*-(3,5-Dimethoxyphenethyl)hexanamide (24)**

To a solution of 2-(3,5-dimethoxyphenyl)ethan-1-amine (0.50 g, 2.76 mmol) and hexanoic acid (0.27 ml, 2.50 mmol) in TAME (10 ml), with a Dean-Stark adaptor attached, was added B(OCH_2_CF_3_)_3_ (0.05 ml, 0.25 mmol). The reaction mixture was heated at reflux for 18 h and the solvent then removed *in vacuo*. The crude product was dissolved in HCl_(aq)_ (1 M, 20 ml), washed with EtOAc (3 x 20 ml), and the combined organic extracts washed with saturated NaHCO_3(aq)_ (3 x 20 ml), dried (Na_2_SO_4_) and the solvent removed under reduced pressure to afford the product as a yellow oil (624 mg, 89%). IR (neat) ν_max_/cm^-1^: 3290, 2931, 2867, 1594, 1540; ^1^H NMR (700 MHz; CDCl_3_) δ 6.33 (3H, s, **3 x Ar-H**), 5.45 (1H, br s, **NH**) 3.77 (6H, s, **2 x OCH_3_**), 3.50 (2H, q, *J*= 6.7 Hz, **C*H*_2_NH**), 2.74 (2H, t, *J* = 6.7 Hz, **C*H*_2_CH_2_NH**), 2.10 (2H, t, *J* = 7.6 Hz, **NHCOC*H*_2_**), 1.59 (2H, pent, *J* = 7.6 Hz, **COCH_2_C*H*_2_**), 1.33-1.23 (4H, m, **2 x** **CH_2_**), 0.87 (3H, t, *J* = 7.1 Hz, **CH_3_**); ^13^C NMR (176 MHz; CDCl_3_) δ 173.3, 161.1, 141.4, 106.9, 96.5, 55.4, 40.4, 37.0, 36.1, 31.6, 25.6, 22.5, 14.0; *m/z* [ES+] 302 ([M + Na]^+^, 15%); 280 ([M + H]^+^, 100%), 182 ([MH - C_6_H_11_O]^+^, 7%); *m/z* [HRMS, ES+] found [M + H]^+^ 280.1912, C_16_H_26_NO_3_ requires 280.1913.

**6-Methoxy-1-pentyl-3,4-dihydroisoquinoline (25)**

To a solution of *N*-(3-methoxyphenethyl)hexanamide (**22**, 570 mg, 2.29 mmol) in xylene (10 ml), under argon, was added POCl_3_ (1.00 ml, 10.7 mmol), dropwise. The reaction was warmed to 130 ^o^C and stirred for 18 h. The reaction was cooled to RT before quenching by the addition of water (3 ml) and was then stirred for 30 min. The solvent was removed under reduced pressure, water (10 ml) was added and the pH increased to 12 with NaOH_(aq)_ (1 M). The aqueous layer was then extracted with EtOAc (3 x 20 ml), the combined organic layers dried (Na_2_SO_4_) and the solvent removed *in vacuo* to yield the product as an orange oil (419 mg, 79%). IR (neat) ν_max_/cm^‑1^: 2931, 2856, 1505, 1568; ^1^H NMR (400 MHz; CDCl_3_) δ 7.42 (1H, d, *J* = 8.5 Hz, **8‑H**), 6.78 (1H, dd, *J* = 8.5 and 2.6 Hz, **7‑H**), 6.70 (1H, d, *J* = 2.6 Hz, **5-H**), 3.82 (3H, s, **OCH_3_**), 3.62 (2H, t, *J* = 7.4 Hz, **CH_2_N**), 2.68-2.63 (4H, m, **2 x CH_2_**), 1.63 (2H, pent, 7.6 Hz, **CCH_2_CH_2_**), 1.39-1.31 (4H, m, **2 x CH_2_**), 0.88 (3H, t, *J* = 7.0 Hz, **CH_3_**); ^13^C NMR (151 MHz; CDCl_3_) δ 167.4, 161.1, 140.3, 127.1, 122.7, 113.0, 112.0, 55.4, 46.8, 36.2, 32.0, 27.3, 27.0, 22.7, 14.2; *m/z* [ES+] 232 ([M + H]^+^, 100%); *m/z* [HRMS, ES+] found [M + H]^+^ 232.1701. C_15_H_22_NO requires 232.1702.

**6,7-Dimethoxy-1-pentyl-3,4-dihydroisoquinoline (26)**^16^

To a solution of *N*-(3,4-dimethoxyphenethyl)hexanamide (**23**, 1.00 g, 3.58 mmol) in xylene (10 ml), under argon, was added POCl_3_ (1.68 ml, 17.9 mmol), dropwise. The reaction was warmed to 130 ^o^C and stirred for 18 h. It was cooled to RT, quenched by the addition of water (3 ml) and then stirred at RT for 30 min. The solvent was removed under reduced pressure, water (20 ml) was added and the pH increased to 10 with NaOH_(aq)_ (1 M). The aqueous layer was then extracted with EtOAc (2 x 20 ml) and Et_2_O (2 x 20 ml) and the solvent removed from the combined organic layers under reduced pressure to yield the product as a pale brown solid (785 mg, 84%). Mp. 43-47 ^o^C (EtOAc); IR (neat) ν_max_/cm^-1^: 2927, 2821, 1629, 1603, 1573, 1509; ^1^H NMR (600 MHz; CDCl_3_) δ 7.00 (1H, s, **Ar‑H**) 6.69 (1H, s, **Ar-H**), 3.91 (3H, s, **OCH_3_**), 3.90 (3H, s, **OCH_3_**), 3.62 (2H, t, *J* = 7.7 Hz, **3-H_2_**), 2.67 (2H, t, *J* = 7.7 Hz, **4-H_2_**), 2.60 (2H, t, *J* = 7.6 Hz, **CCH_2_**), 1.65 (2H, pent, *J* = 7.6 Hz, **CCH_2_C*H*_2_**), 1.36 (4H, m, **2 x** **CH_2_**), 0.89 (3H, t, *J* = 7.1 Hz, **CH_3_**); ^13^C NMR (151 MHz; CDCl_3_) δ 168.0, 150.8, 147.5, 131.8, 122.1, 110.5, 109.0, 56.4, 56.1, 47.1, 36.2, 31.9, 27.0, 26.0, 22.6, 14.2; *m/z* [ES+] 262 ([M + H]^+^, 100%), 232 ([MH - OMe]^+^ 5%).

**6,8-Dimethoxy-1-pentyl-3,4-dihydroisoquinoline** (**27**)

To a solution of *N*-(3,5-dimethoxyphenethyl)hexanamide (**24**, 0.50 g, 1.79 mmol) in xylene (10 ml) under argon, was added POCl_3_ (0.83 ml, 8.90 mmol), dropwise. The reaction was heated at reflux for 18 h. The reaction was quenched by the dropwise addition of MeOH (5 ml) at RT and was stirred for 30 min. The solvent was then removed under reduced pressure, EtOAc (25 ml) was added and the organic layer washed with saturated NaHCO_3(aq)_ (3 x 25 ml). The organic phase was extracted with HCl_(aq)_ (0.5 M, 3 x 25 ml), the acidic layers were combined, and the pH was adjusted to 13 before extraction with EtOAc (3 x 25 ml). The combined organic layers were removed *in vacuo* to afford the product as a yellow oil (324 mg, 69%). IR (neat) ν_max_/cm^-1^: 2933, 2840, 1598, 1570; ^1^H NMR (700 MHz; CDCl_3_) δ 6.37 (1H, d, *J* = 2.3 Hz, **Ar-H**), 6.34 (1H, d, *J* = 2.3 Hz, **Ar-H**), 3.83 (6H, m, **2 x** **OCH_3_**), 3.46 (2H, t, *J* = 7.0 Hz, **3-H_2_**), 2.80 (2H, t, *J* = 7.8 Hz, **CCH_2_**), 2.54 (2H, t, *J* = 7.0 Hz, **4-H_2_**), 1.49 (2H, m, **CCH_2_C*H*_2_**), 1.32-1.29 (4H, m, **2 x CH_2_**), 0.87 (3H, t, *J*= 7.0 Hz, **CH_3_**); ^13^C NMR (176 MHz; CDCl_3_) δ 167.9, 161.7, 158.7, 143.3, 112.8, 104.2, 97.4, 55.5, 48.4, 40.2, 32.2, 28.4, 27.4, 22.7, 14.3; *m/z* [ES+] 262 ([M + H]^+^, 100%); *m/z* [HRMS, ES+] found [M + H]^+^ 262.1806, C_16_H_24_NO_3_ requires 262.1807.

**6-Methoxy-1-pentyl-1,2,3,4-tetrahydroisoquinoline.HCl (18).HCl**

To a solution of 6-methoxy-1-pentyl-3,4-dihydroisoquinoline (**25**, 46 mg, 0.20 mmol) in anhydrous MeOH (5 ml) under argon, was added NaBH_4_ (15 mg, 0.40 mmol). The reaction was stirred at RT for 18 h. The reaction was quenched by the dropwise addition of water (5 ml) and was stirred for 30 min. The solvent was removed under reduced pressure and EtOAc (20 ml) added which was washed with saturated NaHCO_3(aq)_ (3 x 20 ml). The organic phase was dried (Na_2_SO_4_) and the solvent removed under reduced pressure to afford the product as a colourless solid (39 mg, 84%). To prepare the product for biological characterisation a portion of the product was further purified by preparative HPLC (Method 4, Gradient A, Rt = 19.1 min). The purified product was dissolved in HCl_(aq)_ (0.01 M) and lyophilised to obtain the hydrochloride salt of the product as a white solid (12 mg). Mp. 173-175 ^o^C (H_2_O): IR (neat, ν_max_/cm^‑1^): 2741, 1610, 1594, 1247; ^1^H NMR (600 MHz; D_2_O) δ 7.21 (1H, d, *J* = 8.8 Hz, **8-H**), 6.89 (1H, dd, *J* = 8.8 and 2.8 Hz, **7-H**), 6.83 (1H, d, *J* = 2.8 Hz, **5-H**), 4.46 (1H, dd, *J* = 8.3, 5.0 Hz, **1-H**), 3.77 (3H, s, **OCH_3_**), 3.53 (dt, *J* = 12.5, 6.1 Hz, **3-*H*H**), 3.39-3.32 (1H, m, **3-H*H***), 3.13-2.98 (2H, m, **4-CH_2_**), 2.08-1.99 (1H, m, **CHC*H*H**), 1.92-1.82 (1H, m, **CHH*H***), 1.39 (2H, m, **CHCH_2_C*H*_2_**), 1.36-1.22 (4H, m, **2 x CH_2_**), 0.82 (3H, t, *J* = 7.1 Hz, **CH_3_**); ^13^C NMR (151 MHz; D_2_O) δ 158.9, 133.7, 128.6, 125.4, 114.1, 114.0, 67.2 (**dioxane standard**) 56.0, 55.8, 39.8, 33.7, 31.3, 25.5, 24.8, 22.3, 13.8; *m/z* [ES+] 234 ([M + H]^+^, 100%); *m/z* [HRMS, ES+] found [M + H]^+^ 234.1855, C_15_H_24_NO requires 234.1852.

**6,7-Dimethoxy-1-pentyl-1,2,3,4-tetrahydroisoquinoline.HCl (20.HCl)**^16^

To a solution of 6,7-dimethoxy-1-pentyl-3,4-dihydroisoquinoline (**26**, 250 mg, 0.96 mmol) in anhydrous MeOH (10 ml) under argon, was added NaBH_4_ (72 mg, 1.9 mmol). The reaction was stirred at RT for 18 h. The reaction was quenched by the dropwise addition of water (5 ml) and stirred for 30 min. The solvent was removed under reduced pressure and EtOAc (20 ml) added which was washed with saturated NaHCO_3(aq)_ (3 x 20 ml). The organic layer was dried (Na_2_SO_4_) and the solvent removed under reduced pressure to afford the product as a colourless solid (230 mg, 91%). To prepare the product for biological characterisation a portion of the product was further purified by preparative HPLC (Method 4, Gradient A, Rt = 21.60 min). The purified product was dissolved in HCl_(aq)_ (0.01 M) and lyophilised to obtain the hydrochloride salt as a white solid (25 mg). Mp. 182-185 ^o^C (H_2_O) (lit 185-188 ^o^C);^16^ IR (neat) ν_max_/cm^-1^: 3358, 2997, 1673, 1516; ^1^H NMR (700 MHz; D_2_O) δ 6.80 (1H, s, **5‑H**), 6.75 (1H, s, **8-H**), 4.40 (1H, dd, *J* = 8.5 and 4.8 Hz, **1‑H**), 3.50 (1H, app. dt, *J* = 12.4, 6.0 Hz, **3-*H*H**), 3.30 (1H, ddd, *J* = 12.8, 7.6, 5.7 Hz, **3‑H*H***), 3.00-2.87 (2H, m, **4-H_2_**), 1.98 (1H, m, **CHC*H*H**), 1.91-1.79 (1H, m, **CHCH*H***), 1.42-1.35 (2H, m, **CH_2_**), 1.33-1.22 (4H, m, **2 x CH_2_**), 0.81 (3H, t, *J* = 7.1 Hz, **CH_3_**); ^13^C NMR (151 MHz; D_2_O) δ 148.4, 147.7, 125.2, 124.9, 112.4, 110.1, 67.2 (**dioxane standard**) 56.4, 56.3, 55.8 39.8, 33.9, 31.4, 24.9, 24.9, 22.3, 13.8; *m/z* [ES+] 264 ([M + H]^+^, 100%); *m/z* [HRMS, ES+] found [M + H]^+^ 264.1957, C_16_H_26_NO_2_ requires 264.1964.

**6,8-Dimethoxy-1-pentyl-1,2,3,4-tetrahydroisoquinoline.HCl (21.HCl)**

To a solution of 6,8-dimethoxy-1-pentyl-3,4-dihydroisoquinoline (**28**, 50 mg, 0.2 mmol) in anhydrous MeOH (5 ml) under argon, was added NaBH_4_ (15 mg, 0.4 mmol). The reaction was stirred at RT for 18 h. It was quenched by the dropwise addition of water (5 ml) and stirred for 30 min. The pH of the reaction mixture was reduced to 10 by the addition of NaOH_(aq)_ (1 M), and the aqueous layer extracted with EtOAc (3 x 20 ml). The combined organic layers were dried (Na_2_SO_4_) and the solvent removed under reduced pressure to afford the product as a colourless solid (47 mg, 93%). To prepare the product for biological characterisation the product was further purified by preparative HPLC (Method 4, Gradient G, run time: 15 min, flow rate: 15 ml/min, Rt = 5.1 min). The solid was then dissolved in HCl_(aq)_ (0.5 M) and lyophilised to yield the hydrochloride salt as a white solid (17.7 mg). IR (neat) ν_max_/cm^‑1^: 2930, 2641, 1593, 1490; ^1^H NMR (700 MHz; D_2_O) δ 6.47 (1H, d, *J* = 2.3 Hz, **5-H**), 6.43 (1H, d, *J* = 2.3 Hz, **7-H**), 4.56 (1H, dd, *J*= 9.4 and 3.0 Hz, **1-H**), 3.78 (3H, s, **OCH_3_**), 3.77 (3H, s, **OCH_3_**), 3.45 (1H, m, **3-*H*H**), 3.35 (1H, m, **3‑H*H***) 3.05-2.95 (2H, m, **4-CH_2_**), 1.87-1.82 (1H, m, **CHC*H*H**), 1.75-1.69 (1H, m, **CHCH*H***), 1.43-1.36 (2H, m, **CH_2_**), 1.31-1.24 (4H, m, **2 x CH_2_**), 0.93 (3H, m, **CH_3_**); ^13^C NMR (176 MHz; D_2_O) δ 160.0, 157.6, 133.8, 115.0, 105.5, 97.9, 67.2 (**dioxane standard**), 56.2, 56.1, 51.7, 37.2, 32.2, 31.1, 25.4, 25.2, 22.3, 13.8; *m/z* [ES+] 264 ([M + H]^+^, 100%); *m/z* [HRMS, ES+] found [M + H]^+^ 264.1970. C_16_H_26_NO_3_ requires 264.1964.

***N*-(2-Hydroxy-2-phenylethyl)hexanamide (29)**

To a solution of hexanoic acid (0.625 ml, 5.00 mmol) and 2-amino-1-phenylethanol (754 mg, 5.50 mmol) in TAME, with a Dean-Stark adapter attached, was added B(OCH_2_CF_3_)_3_ (0.11 ml, 0.5 mmol). The reaction was heated at reflux for 18 h and the solvent removed *in vacuo*. Water (30 ml) was added to the crude product and the pH was adjusted to 3 with HCl_(aq)_ (0.5 M). The aqueous layer was extracted with Et_2_O (3 x 30 ml), and the combined organic layers washed with water (2 x 30 ml) and brine (30 ml). The solvent was removed from the organic layer under vacuum, water (20 ml) was added and the pH adjusted to 9 with NaOH_(aq)_ (1 M). The aqueous layer was then extracted with EtOAc (3 x 20 ml), dried (Na_2_SO_4_), and the solvent removed *in vacuo* to yield the product as a white solid (652 mg, 50%). Mp. 78-81 ^o^C (EtOAc); IR (neat) ν_max_/cm^-1^: 3384, 3287, 2924, 1609, 1560; ^1^H NMR (600 MHz; CDCl_3_) δ 7.37-7.33 (4H, m, **2-H** & **3-H**), 7.31-7.27 (1H, m, **4‑H**), 5.90 (1H, br s, **NH_2_**), 4.85 (1H, dd, *J* = 7.7 and 3.3 Hz, **C*H*OH**), 3.71 (1H, ddd, *J* = 14.2, 6.8 and 3.3 Hz, **C*H*HNH**), 3.36-3.31 (1H, ddd, *J* = 14.2, 7.7 and 5.2 Hz, **CH*H*NH**), 2.18 (2H, t, *J* = 7.4 Hz, **COCH_2_**), 1.64-1.59 (2H, q, *J* = 7.4 Hz, **COCH_2_C*H*_2_**), 1.34-1.25 (4H, m, **2 x CH_2_**), 0.90 (3H, t, *J* = 7.0 Hz, **CH_3_**); ^13^C NMR (151 MHz; CDCl_3_) δ 174.0, 141.9, 128.6, 127.9, 125.9, 74.0, 47.7, 36.7, 31.5, 25.5, 22.5, 12.0; *m/z* [ES+] 258 ([M + Na]^+^, 47%) 236 ([M + H]^+^, 70%), 218 ([M - OH]^+^, 100%), 120 ([M ‑ O_2_C_6_H_12_]^+^, 92%); *m/z* [HRMS, ES+] found [M + H]^+^ 236.1654. C_14_H_22_NO_2_ requires 236.1650.

**1-Pentylisoquinoline.HCl (28.HCl)**^18^

To a solution of *N*-(2-hydroxy-2-phenylethyl)hexanamide (**29**) (300 mg, 1.28 mmol) in xylene (15 ml) under anhydrous conditions and argon, was added POCl_3_ (1.20 ml, 12.8 mmol). The reaction mixture was stirred for 18 h and cooled to RT before any remaining POCl_3_ was quenched by addition of water (3 ml) and the reaction was stirred for 1 h. The solvent was then removed under reduced pressure, water (10 ml) added and the pH was increased to 14 by the addition of NaOH_(aq)_ (1 M). The aqueous layer was extracted with Et_2_O (4 x 20 ml), the organic layers were combined, dried (Na_2_SO_4)_ and the solvent was removed *in vacuo* to yield the product as a yellow oil (41 mg, 14%). To prepare the product for biological characterisation a portion of the product was further purified by preparative HPLC (Method 4, Gradient H, run time: 35 min, Rt = 8.1 min). The purified product was dissolved in HCl_(aq)_ (0.01 M) and lyophilised to obtain the hydrochloride salt of the product as a white solid (3.2 mg). IR (neat) ν_max_/cm^-1^): 3050, 2926, 1642, 1620, 1586, 1562, 1501; ^1^H NMR (600 MHz; CDCl_3_) δ 8.43 (1H, d, *J* = 5.7 Hz, **3-H**), 8.16 (1H, d, *J* = 8.2 Hz, **Ar-H**), 7.80 (1H, d, *J* = 8.2 Hz, **Ar-H**), 7.65 (1H, app. t, *J* = 7.6 Hz, **Ar-H**), 7.58 (1H, app.t, *J* = 7.6 Hz, **Ar-H**), 7.49 (1H, d, *J* = 5.7 Hz, **4-H**), 3.19 (2H, t, *J* = 8.0 Hz, **ArC*H*_2_**), 1.89-1.84 (2H, m, **ArCH_2_*C*H_2_**), 1.49-1.44 (2H, m, **CH_2_**), 1.43-1.37 (2H, m, **CH_2_**), 0.91 (3H, t, *J =* 7.1 Hz, **CH_3_**); ^13^C NMR (176 MHz; MeOD) δ 163.2, 140.3, 137.6, 132.3, 131.5, 129.6, 128.9, 127.6, 125.3, 32.8, 32.7, 31.0, 23.4, 14.2; *m/z* [ES+] 200 ([M + H]^+^, 100%); *m/z* [HRMS, ES+] found [M + H]^+^ 200.1439, C_14_H_18_N requires 200.1434.

**6-Methoxy-1-pentylisoquinoline.HCl (30.HCl)**

6-Methoxy-1-pentyl-3,4-dihydroisoquinoline (**25**, 400 mg, 1.72 mmol) was mixed with Pd/C (400 mg) in a microwave vial. The reaction mixture was heated to 150 ^o^C for 45 min in a microwave (Personal Chemistry Smith Creator Microwave Assisted Organic Synthesiser). The reaction mixture was dissolved in EtOAc and filtered through Celite®, washing with EtOAc. The solvent was removed under reduced pressure and the crude product purified twice by column chromatography (30% EtOAc in petroleum ether 40-60, then 10% EtOAc in petroleum ether 40-60) to afford the product as an orange solid (119 mg, 30%). To prepare the product for biological characterisation a portion of the product was purified by preparative HPLC (Method 4, Gradient E, run time: 35 min, Rt = 9.8 min). The purified product was dissolved in HCl_(aq)_ (0.01 M) and lyophilised to obtain the hydrochloride salt as a white solid (4.2 mg). IR (neat) ν_max_/cm^‑1^: 3273 br, 2922, 1626; ^1^H NMR (600 MHz; CD_3_OD) δ 8.52 (1H, d, *J* = 9.3 Hz, **8‑H**), 8.27 (1H, d, *J* = 6.7 Hz, **3-H**), 8.12 (1H, d, *J*= 6.7, **4-H**), 7.63 (1H, d, *J* = 2.5 Hz, **5-H**), 7.60 (1H, dd, *J* = 9.3 and 2.5 Hz, **7‑H**), 4.09 (3H, s, **OCH_3_**), 3.48 (2H, t, *J* = 8.0 Hz, **CCH_2_**), 1.89 (2H, m, **CH_2_**), 1.50-1.39 (4H, m, **2 x** **CH_2_**), 0.94 (3H, t, *J* = 7.2 Hz, **CH_3_**); ^13^C NMR (176 MHz; CD_3_OD) δ 167.2, 161.2, 143.4, 132.1, 130.8, 125.0, 123.5, 122.7, 107.5, 57.1, 32.7, 32.6, 31.07, 23.4, 14.2; *m/z* [ES+] 230 ([M + H]^+^, 100%); *m/z* [HRMS, ES+] found [M + H]^+^ 230.1536. C_15_H_20_NO requires 230.1539.

**6,7-Dimethoxy-1-pentylisoquinoline.HCl (31.HCl)**

####

6,7-Dimethoxy-1-pentyl-3,4-dihydroisoquinoline (**26**, 450 mg, 1.72 mmol) was mixed with Pd/C (300 mg) in sealed microwave vial and heated to 180 ^o^C for 36 h. The reaction mixture was dissolved in EtOAc and filtered through Celite®, washing with EtOAc. The solvent was removed under reduced pressure and the crude product purified by flash column chromatography (3% MeOH in CH_2_Cl_2_) to afford the product as a brown solid (121 mg, 27%). To prepare the product for biological characterisation a portion of the product was further purified by preparative HPLC (Method 4, Gradient D, run time: 50 min, Rt = 12.02 min). The purified product was dissolved in HCl_(aq)_ (0.01 M) and lyophilised to obtain the hydrochloride salt as a white solid (6.0 mg). IR (film) ν_max_/cm^‑1^: 3370 br, 2922, 1656, 1625, 1508; ^1^H NMR (600 MHz; D_2_O) δ 8.32 (1H, d, *J* = 5.6 Hz, **3-H**), 7.38 (1H, d, *J* = 5.6 Hz, **4-H**), 7.34 (1H, s, **8-H**), 7.06 (1H, s, **5-H**), 4.04 (3H, s, **OCH_3_**), 4.03 (3H, s, **OCH_3_**), 3.22 (2H, t, *J* = 7.7 Hz, **CCH_2_**), 1.86 (2H, pent, *J* = 7.7 Hz, **CCH_2_C*H*_2_**), 1.50-1.35 (4H, m, **2 x CH_2_**), 0.92 (3H, t, *J* = 7.0 Hz, **CH_3_**); ^13^C NMR (176 MHz; D_2_O) δ 157.4, 157.1, 152.4, 137.4, 129.3, 122.7, 122.2, 107.1, 105.3, 57.1, 56.8, 31.5, 31.3 29.0, 22.2, 13.7; *m/z* [ES+] 260 ([M + H]^+^, 100%); *m/z* [HRMS, ES+] found [M + H]^+^ 260.1643. C_16_H_22_NO_2_ requires 260.1651.

**6,8-Dimethoxy-1-pentylisoquinoline.HCl (32.HCl)**

6,8-Dimethoxy-1-pentyl-3,4-dihydroisoquinoline (**27**, 150 mg, 0.57 mmol), was mixed together with Pd/C (150 mg) in a microwave vial, exposed to the air. The reaction mixture was warmed to 180 ^o^C for 90 h. The reaction mixture was added to EtOAc before filtration through Celite®, washing with EtOAc. The solvent was removed under reduced pressure and the crude product purified by flash column chromatography (2–5% MeOH in CH_2_Cl_2_) to give the product as a yellow oil (50 mg, 34%). The product was dissolved in in HCl_(aq)_ (0.1 M) and lyophilised to afford the hydrochloride salt, which was purified by preparative HPLC (Method 4, Gradient A, run time: 20 min, flow rate: 15 ml/min, Rt = 16.0 min) to prepare the product for biological evaluation. The resulting solid was dissolved in HCl_(aq)_ (0.1 M) and lyophilised to obtain the hydrochloride salt as a yellow solid (9.2 mg). IR (neat) ν_max_/cm^-1^: 3368 br, 2930, 2678, br, 1612, 1456, 1396; ^1^H NMR (700 MHz; D_2_O) δ 7.91 (1H, d, *J* = 6.7 Hz, **3-H**), 7.70 (1H, d, *J* = 6.7 Hz, **4‑H**), 6.89 (1H, d, *J* = 2.2 Hz, **Ar-H**), 6.69 (1H, d, *J* = 2.2 Hz, **Ar-H**), 3.94 (3H, s, **OCH_3_**), 3.91 (3H, s, **OCH_3_**), 3.24 (2H, d, *J* = 8.0 Hz, **CHCH_2_**), 1.58 (2H, pent, *J* = 7.7 Hz, **CH_2_**), 1.32 (4H, m, **2 x** **CH_2_**), 0.84 (3H, *J* = 7.2 Hz, **CH_3_**); ^13^C NMR (176 MHz; D_2_O) 166.9, 161.4, 160.1, 143.2, 130.4, 122.3, 114.5, 102.5, 100.3, 67.2 (**dioxane standard**), 56.9, 56.8, 36.0, 31.7, 29.9, 22.2, 13.8; *m/z* [ES+] 260 ([M + H]^+^, 100%); [HRMS, ES+] found [M + H]^+^ 260.1660. C_16_H_22_NO_2_ requires 260.1651.

**1-Pentylisoquinolin-6-ol.HCl (33).HCl**

To a solution of 6-methoxy-1-pentylisoquinoline (**30**, 40.0 mg, 0.17 mmol) in anhydrous CH_2_Cl_2_ (3 ml) was added a solution of BBr_3_ in CH_2_Cl_2_ (3 ml, 1 M, 3.0 mmol). The reaction mixture was heated at reflux for 18 h, before a further addition of BBr_3_ in CH_2_Cl_2_ (3 ml, 1 M). The reaction mixture was then left to stir for 10 h. The reaction was quenched by the addition of MeOH (5 ml) and the solvent was removed under reduced pressure. The crude product was then repeatedly azeotroped with MeOH until no fumes were evolved upon addition of MeOH. The product was purified by preparative HPLC (Method 4, Gradient F, run time: 35 min, Rt = 8.13 min) to yield the product, which was dissolved in HCl_(aq)_ (0.1 M) and lyophilised to yield **33** as the hydrochloride salt (12.8 mg, 29%). IR (film) ν_max_/cm^‑1^): 2914, 2648, 1750, 1609; ^1^H NMR (600 MHz; CD_3_OD) δ 8.50 (1H, d, *J* = 9.3 Hz, **8-H**), 8.17 (1H, d, *J* = 6.7 Hz, **3-H**), 7.97 (1H, d, *J*= 6.7, **4-H**), 7.53 (1H, dd, *J* = 9.3 and 2.4 Hz, **7- H**), 7.39 (1H, d, *J* = 2.3 Hz, **5‑H**), 3.46 (2H, t, *J* = 7.8 Hz, **CCH_2_**), 1.88 (2H, pent, *J* = 7.8 Hz, **CH_2_**), 1.50-1.40 (4H, m, **2 x CH_2_**), 0.94 (3H, t, *J* = 7.2 Hz, **CH_3_**); ^13^C NMR (176 MHz; D_2_O) δ 163.5, 160.2, 141.8, 130.9, 130.1, 123.2, 122.2, 121.0, 110.0, 31.6, 31.2, 29.6, 22.2, 13.6 ; *m/z* [ES+] 216 ([M + H]^+^, 100%); *m/z* [HRMS, ES+] found [M + H]^+^ 216.1385. C_14_H_18_NO requires 216.1383.

**1-Pentylisoquinoline-6,7-diol.HCl (34.HCl)**^14^

To a solution of 6,7-dimethoxy-1-pentylisoquinoline (**31**, 100 mg, 0.39 mmol) in anhydrous CH_2_Cl_2_ (5 ml) at 0 ^o^C, was added a solution of BBr_3_ in CH_2_Cl_2_ (1.35 ml, 1 M, 1.35 mmol). The solution was stirred at RT for 18 h, was then quenched by the addition of MeOH (5 ml) and was stirred for 30 min. The solvent was removed under reduced pressure and the crude product azeotroped repeatedly with MeOH until no fumes were evolved upon the addition of MeOH. The product was then purified by preparative HPLC (Method 4, Gradient E, Rt = 8.51 min) to yield the product which was dissolved in HCl_(aq)_ (0.01 M) and lyophilised to yield **34** as the hydrochloride salt (9.3 mg, 9%). IR (film) ν_max_/cm^‑1^: 3203 br, 2930 br, 2773 br, 1727, br. 1633, 1609, 1523; ^1^H NMR (700 MHz; CD_3_OD) δ 8.05 (1H, d, *J* = 6.6 Hz, **3-H**), 7.91 (1H, d, *J* = 6.6 Hz, **4‑H**), 7.68 (1H, s, **8-H**), 7.41 (1H, s, **5-H**), 3.36 (2H, t, *J* = 7.8 Hz, **CCH_2_**), 1.87 (2H, pent, *J*= 7.8 Hz, **CH_2_**), 1.49-1.40 (4H, m, **2 x CH_2_**), 0.95 (3H, t, *J* = 7.2 Hz, **CH_3_**); ^13^C NMR (176 MHz; CD_3_OD) δ 157.9, 157.2, 152.3, 137.8, 128.8, 123.2 , 122.3, 110.7, 109.4, 32.7, 32.5, 30.5, 23.4, 14.2; *m/z* [ES+] 232 ([M + H]^+^, 100%); *m/z* [HRMS, ES+] found [M + H]^+^ 232.1331. C_14_H_18_NO_2_ requires 231.1332.

**1-Pentylisoquinoline-6,8-diol.HCl (35.HCl)**

To a solution of 6,8-dimethoxy-1-pentylisoquinoline (**32**, 18 mg, 70 µmol) in CH_2_Cl_2_ (2 ml) was added BBr_3_ in CH_2_Cl_2_ (1 M, 1.0 ml, 1.0 mmol). The reaction was stirred for 18 h at RT, before the further addition of BBr_3_ in CH_2_Cl_2_ (1 M, 0.5 ml, 0.5 mmol). The reaction mixture was stirred for a further 6 h at RT before being quenched by the dropwise addition of MeOH (5 ml). MeOH was subsequently added and evaporated until no further gas was observed upon addition of MeOH. The product was dissolved in HCl_(aq)_ (0.1 M) and lyophilised to yield the hydrochloride salt which was purified by preparative HPLC (Method 4, Gradient C, run time: 30 min, flow rate: 15 ml/min, Rt = 20.3 min). To prepare the product for biological characterisation the product was dissolved in HCl_(aq)_ (0.1 M) and lyophilised to yield **35**.HCl as a white solid (5.3 mg, 33%). IR (neat) ν_max_/cm^-1^: 3396, 3201, 3108 br, 2925, 2857, 1612, 1580; ^1^H NMR (700 MHz; D_2_O) δ 7.79 (1H, d, *J* = 6.8 Hz, **3-H**), 7.57 (1H, d, *J* = 6.8 Hz, **4‑H**), 6.71 (1H, s, **Ar-H**), 6.58 (1H, s, **Ar-H**), 3.35 (2H, t, *J* = 7.8 Hz, **CCH_2_**), 1.66 (2H, pent, *J* = 7.8 Hz, **CH_2_**), 1.39-1.24 (4H, m, **2 x** **CH_2_**), 0.83 (3H, t, *J* = 7.2 Hz, **CH_3_**); ^13^C NMR (176 MHz; D_2_O) δ 164.6, 161.1, 160.8, 142.6, 129.4, 121.7, 113.1, 105.4, 103.2, 67.2 (**dioxane standard**), 35.6, 31.5, 29.7, 22.1, 13.7; *m/z* [ES+] 232 ([M + H]^+^, 100%); *m/z* [HRMS, ES+] found [M + H]^+^ 232.1331. C_14_H_18_NO_2_ requires 232.1332.

**References**

1. Yang, S. H., Song, C. H., Van, H. T. M., Park, E., Khadka, D. B., Gong, E. Y., Lee, K. & Cho, W. J. SAR based design of nicotinamides as a novel class of androgen receptor antagonists for prostate cancer. *J. Med. Chem.* **56**, 3414–3418 (2013).

2. Lamming, E. D. Synthesis of N-heterocycles as anti-asthma drugs and compounds with antimycobacterial properties. (University College London, 2015).

3. Smith, K., El-Hiti, G. & Alshammari, M. Directed Lithiation of N′-[2-(4-Methoxyphenyl)ethyl]-N,N-dimethylurea and tert-Butyl [2-(4-Methoxyphenyl)ethyl]carbamate. *Synthesis (Stuttg).* **46**, 394–402 (2013).

4. Kinney, W. A., McDonnell, M. E., Zhong, H. M., Liu, C., Yang, L., Ling, W., Qian, T., Chen, Y., Cai, Z., Petkanas, D. & Brenneman, D. E. Discovery of KLS-13019, a Cannabidiol-Derived Neuroprotective Agent, with Improved Potency, Safety, and Permeability. *ACS Med. Chem. Lett.* **7**, 424–428 (2016).

5. Tahirovic, Y. A., Geballe, M., Gruszecka-Kowalik, E., Myers, S. J., Lyuboslavsky, P., Le, P., French, A., Irier, H., Choi, W., Easterling, K., Yuan, H., Wilson, L. J., Kotloski, R., McNamara, J. O., Dingledine, R., Liotta, D. C., Traynelis, S. F. & Snyder, J. P. Enantiomeric Propanolamines as selective N-Methyl-d-aspartate 2B Receptor Antagonists. *J. Med. Chem.* **51**, 5506–5521 (2008).

6. Liang, J. T., Liu, J., Shireman, B. T., Tran, V., Deng, X. & Mani, N. S. A Practical Synthesis of Regioisomeric 6- and 7-Methoxytetrahydro-3-benzazepines. *Org. Process Res. Dev.* **14**, 380–385 (2010).

7. DeRuiter, J., Clark, C. R. & Noggle, F. T. Gas Chromatographic--Mass Spectrometric and High-Performance Liquid Chromatographic Analyses of the Bromination Products of the Regioisomeric Dimethoxyphenethylamines: Differentiation of Nexus from Five Positional Isomers. *J. Chromatogr. Sci.* **36**, 23–28 (1998).

8. Wang, B., Qin, L., Neumann, K. D., Uppaluri, S., Cerny, R. L. & DiMagno, S. G. Improved Arene Fluorination Methodology for I(III) Salts. *Org. Lett.* **12**, 3352–3355 (2010).

9. Guzman, J. D., Pesnot, T., Barrera, D. A., Davies, H. M., McMahon, E., Evangelopoulos, D., Mortazavi, P. N., Munshi, T., Maitra, A., Lamming, E. D., Angell, R., Gershater, M. C., Redmond, J. M., Needham, D., Ward, J. M., Cuca, L. E., Hailes, H. C. & Bhakta, S. Tetrahydroisoquinolines affect the whole-cell phenotype of Mycobacterium tuberculosis by inhibiting the ATP-dependent MurE ligase. *J. Antimicrob. Chemother.* **70**, 1691–1703 (2015).

10. Wong, M. & de Jesus, O. T. Synthesis and enzymatic decarboxylation of 6-bromo-L-dopa, a potential brain tracer for L-dopa. *J. Label. Compd. Radiopharm.* **24**, 1373–1380 (1987).

11. Ruff, B. M., Bräse, S. & O’Connor, S. E. Biocatalytic production of tetrahydroisoquinolines. *Tetrahedron Lett.* **53**, 1071–1074 (2012).

12. Bailey, P. D., Morgan, K. M., Smith, D. I. & Vernon, J. M. Spiro cyclisations of N-acyliminium ions involving an aromatic π-nucleophile. *Tetrahedron* **59**, 3369–3378 (2003).

13. Pesnot, T., Gershater, M. C., Ward, J. M. & Hailes, H. C. The Catalytic Potential of Coptis japonica NCS2 Revealed - Development and Utilisation of a Fluorescamine-Based Assay. *Adv. Synth. Catal.* **354**, 2997–3008 (2012).

14. Iwasa, K., Moriyasu, M., Tachibana, Y., Kim, H.-S., Wataya, Y., Wiegrebe, W., Bastow, K. F., Cosentino, L. M., Kozuka, M. & Lee, K.-H. Simple Isoquinoline and Benzylisoquinoline Alkaloids as Potential Antimicrobial, Antimalarial, Cytotoxic, and Anti-HIV Agents. *Bioorg. Med. Chem.* **9**, 2871–2884 (2001).

15. Maresh, J. J., Crowe, S. O., Ralko, A. A., Aparece, M. D., Murphy, C. M., Krzeszowiec, M. & Mullowney, M. W. Facile one-pot synthesis of tetrahydroisoquinolines from amino acids via hypochlorite-mediated decarboxylation and Pictet–Spengler condensation. *Tetrahedron Lett.* **55**, 5047–5051 (2014).

16. Craig, P. N., Nabenhauer, F. P., Williams, P. M., Macko, E. & Toner, J. Tetrahydroisoquinolines. I. 1-Alkyl-6,7-dihydroxy-1,2,3,4-tetrahydroisoquinolines 1. *J. Am. Chem. Soc.* **74**, 1316–1317 (1952).

17. Nishihachijo, M., Hirai, Y., Kawano, S., Nishiyama, A., Minami, H., Katayama, T., Yasohara, Y., Sato, F. & Kumagai, H. Asymmetric synthesis of tetrahydroisoquinolines by enzymatic Pictet-Spengler reaction. *Biosci. Biotechnol. Biochem.* **78**, 701–707 (2014).

18. Tang, R. J., Kang, L. & Yang, L. Metal-Free Oxidative Decarbonylative Coupling of Aliphatic Aldehydes with Azaarenes: Successful Minisci-Type Alkylation of Various Heterocycles. *Adv. Synth. Catal.* **357**, 2055–2060 (2015).

**NMR Spectra of the Compounds Tested**

**1-Ethyl-1,2,3,4-tetrahydroisoquinoline-6,7-diol (1a)**

**1-Ethyl-1,2,3,4- tetrahydroisoquinoline-7,8-diol (1b)**

**1-Propyl-1,2,3,4-tetrahydroisoquinoline-6,7-diol (2a)**

**1-Propyl- 1,2,3,4-tetrahydroisoquinoline-7,8-diol (2b)**

**1-Butyl-1,2,3,4-tetrahydroisoquinoline-6,7-diol (3a)**

**1-Butyl-1,2,3,4- tetrahydroisoquinoline-7,8-diol (3b)**

**1-Pentyl-1,2,3,4-tetrahydroisoquinoline-6,7-diol (4a)**

**1-Pentyl-1,2,3,4- tetrahydroisoquinoline-7,8-diol (4b)**

**1-Pentyl-1,2,3,4-tetrahydroisoquinoline-6,7-diol hydrochloride (4a.HCl)**

**1-Hexyl-1,2,3,4-tetrahydroisoquinoline-6,7-diol (5a)**

**1-Hexyl-1,2,3,4- tetrahydroisoquinoline-7,8-diol (5b)**

**1-Heptyl-1,2,3,4-tetrahydroisoquinoline-6,7-diol (6a)**

**1-Heptyl-1,2,3,4- tetrahydroisoquinoline-7,8-diol (6b)**

**1-Octyl-1,2,3,4-tetrahydroisoquinoline-6,7-diol (7a)**

**1-Octyl-1,2,3,4- tetrahydroisoquinoline-7,8-diol (7b)**

**1-Cyclopentyl-1,2,3,4-tetrahydroisoquinoline-6,7-diol (8a)**

**1- Cyclopentyl-1,2,3,4-tetrahydroisoquinoline-7,8-diol (8b)**

**1-(3-Methylbenzyl)-1,2,3,4-tetrahydroisoquinoline-6,7-diol (9a)**

**1-(3- Methylbenzyl)-1,2,3,4-tetrahydroisoquinoline-7,8-diol (9b)**

**1-(Benzo[*d*][1,3]dioxol-5-ylmethyl)-1,2,3,4-tetrahydroisoquinoline-6,7-diol (10a)**

**1-(Benzo[*d*][1,3]dioxol-5-ylmethyl)-1,2,3,4-tetrahydroisoquinoline-7,8-diol (10b)**

**1-Pentyl-1,2,3,4-tetrahydroisoquinolin-6-ol (11a)**

**1-Pentyl-1,2,3,4- tetrahydroisoquinolin-8-ol (11b)**

**1-Pentyl-1,2,3,4-tetrahydroisoquinolin-6-ol.HCl** (**11a.HCl**)

**(1*S*)-1-Pentyl-1,2,3,4-tetrahydroisoquinoline-6,7-diol.HCl ((1*S*)-4a.HCl)**^17^

**(1*S*)-1-Pentyl-1,2,3,4-tetrahydroisoquinolin-6-ol.HCl ((1*S*)-11a,HCl)**

**1-Pentyl-1,2,3,4-tetrahydroisoquinoline-6,8-diol.HCl (12.HCl)**

**5-Bromo-1-pentyl-1,2,3,4-tetrahydroisoquinolin-8-ol (13)**

**5-Bromo-1-pentyl-1,2,3,4-tetrahydroisoquinoline-7,8-diol (14)**

**5-Bromo-1-pentyl-1,2,3,4-tetrahydroisoquinoline-6,8-diol (15)**

**1-(Benzo[*d*][1,3]dioxol-4-yl)-5-bromo-1,2,3,4-tetrahydroisoquinolin-8-ol (36)**

**1-Pentyl-1,2,3,4-tetrahydroisoquinolin-7-ol (16)**

**6-Methoxy-1-pentyl-1,2,3,4-tetrahydroisoquinoline.HCl (18)**

**6,7-Dimethoxy-1-pentyl-1,2,3,4-tetrahydroisoquinoline.HCl (20)**

**6,8-Dimethoxy-1-pentyl-1,2,3,4-tetrahydroisoquinoline.HCl (21)**

**1-Pentylisoquinoline.HCl (28)^18^**

**6-Methoxy-1-pentylisoquinoline.HCl (30)**

**6,7-Dimethoxy-1-pentylisoquinoline.HCl (31)**

**6,8-Dimethoxy-1-pentylisoquinoline.HCl (32)**

**1-Pentylisoquinolin-6-ol.HCl (33)**

**1-Pentylisoquinoline-6,7-diol.HCl (34)**^14^

**1-Pentylisoquinoline-6,8-diol.HCl (35)**
